# Novel Aminopyrimidine-2,4-diones, 2-Thiopyrimidine-4-ones, and 6-Arylpteridines as Dual-Target Inhibitors of BRD4/PLK1: Design, Synthesis, Cytotoxicity, and Computational Studies

**DOI:** 10.3390/ph16091303

**Published:** 2023-09-15

**Authors:** Samar El-Kalyoubi, Samiha A. El-Sebaey, Sherin M. Elfeky, Hanan A. AL-Ghulikah, Mona S. El-Zoghbi

**Affiliations:** 1 Department of Pharmaceutical Organic Chemistry, Faculty of Pharmacy, Port Said University, Port Said 42511, Egypt; 2 Department of Pharmaceutical Organic Chemistry, Faculty of Pharmacy (Girls), Al-Azhar University, Youssef Abbas Street, Cairo 11754, Egypt; 3 Department of Pharmaceutical Organic Chemistry, Faculty of Pharmacy, Mansoura University, Mansoura 355516, Egypt; sherin-el-feky@mans.edu.eg; 4 Department of Chemistry, College of Science, Princess Nourah bint Abdulrahman University, P.O. Box 84428, Riyadh 11671, Saudi Arabia; haalghulikah@pnu.edu.sa; 5 Department of Pharmaceutical Chemistry, Faculty of Pharmacy, Menoufia University, Menoufia, Gamal Abd Al-Nasir Street, Shibin-Elkom 32511, Egypt; mona.said@phrm.menofia.edu.eg

**Keywords:** pyrimidines, pteridines, BRD4 inhibitors, PLK1 inhibitors, cytotoxicity, computational studies, gene expression

## Abstract

Structural-based drug design and solvent-free synthesis were combined to obtain three novel series of 5-arylethylidene-aminopyrimidine-2,4-diones (**4**, **5a**–**c**, **6a**,**b**), 5-arylethylidene-amino-2-thiopyrimidine-4-ones (**7**,**8**), and 6-arylpteridines (**9**,**10**) as dual BRD4 and PLK1 inhibitors. MTT assays of synthesized compounds against breast (MDA-MB-231), colorectal (HT-29), and renal (U-937) cancer cells showed excellent-to-good cytotoxic activity, compared to Methotrexate; MDA-MB-231 were the most sensitive cancer cells. The most active compounds were tested against normal Vero cells. Compounds **4** and **7** significantly inhibited BRD4 and PLK1, with IC_50_ values of 0.029, 0.042 µM, and 0.094, 0.02 µM, respectively, which are nearly comparable to volasertib (IC_50_ = 0.017 and 0.025 µM). Compound **7** triggered apoptosis and halted cell growth at the G2/M phase, similarly to volasertib. It also upregulated the BAX and caspase-3 markers while downregulating the Bcl-2 gene. Finally, active compounds fitted the volasertib binding site at BRD4 and PLK1 and showed ideal drug-like properties and pharmacokinetics, making them promising anticancer candidates.

## 1. Introduction

Cancer is a serious public health concern with global implications, ranking as one of the top two leading causes of mortality before the age of 70 in many countries [1,2]. While surgical removal remains a common treatment option for various malignancies, there are instances where this approach may not be feasible due to the cancer’s location, severity, and degree of invasiveness. As an alternative, non-surgical methods, including chemotherapy, targeted treatment, immunotherapy, and radiation, can be used to treat various malignancies [3,4]. Despite all of these options, treating cancer remains a huge issue for clinicians and researchers due to the complexity of the disease arising from the unrestrained proliferation and division of aberrant cells within the body, which is triggered by abnormal regulation of multiple signaling pathways. Among these include the dysregulation of many kinases and the overexpression of anti-apoptotic proteins, such as Bcl-2 [5,6]. Some of the available cancer medications are now linked to drug resistance and recurrence, demanding the development of innovative therapeutic strategies, such as conjunction medicines, which target several signaling pathways [7].

Dual inhibitors are an important domain in the search for new cancer therapies with the potential to overcome drug resistance and improve therapeutic outcomes for cancer patients via selectively targeting multiple pathways [8]. Several drug combinations utilizing kinase inhibitors and epigenetic regulatory molecules were proved to exhibit greater efficacy in both preclinical cancer models and clinical trials compared to single-drug therapy [9].

Bromodomain-containing protein 4 (BRD4) is a protein with an epigenetic reader domain that belongs to the bromodomain and extra-terminal (BET) family of proteins. These proteins are essential in regulating gene expression for cell proliferation (e.g., c-Myc, Aurora B) and survival (e.g., Bcl-2) by binding with acetylated lysine residues on histones and recruiting transcriptional machinery [10,11]. BRD4 is important for a variety of cellular functions involving cell cycle progression, DNA damage response, and inflammation, which has been associated with the development of several diseases, including cancer and inflammatory disorders [12]. The overexpression of BRD4 has been demonstrated in various kinds of cancer, such as breast, lung, prostate, and hematological malignancies [13]. In addition to its regulation of gene expression, recent research has proved that BRD4 is also involved in other cellular functions that play a role in cancer progression. One instance is the regulation of DNA damage response pathways through the interaction of BRD4 with essential proteins involved in DNA repair [14]. As a result, the identification of potential novel BRD4 dual-target inhibitors could be a fruitful approach to treating cancer.

On the other hand, polo-like kinase 1 (PLK1), a serine/threonine kinase, is essential in regulating the cell cycle, especially in mitosis [15]. Dysregulation of PLK1 has been linked to the emergence of many cancers, including breast, lung, pancreatic, prostate, and ovarian cancers, as well as non-Hodgkin’s lymphomas [16]. Studies have shown that PLK1 inhibition causes cell cycle arrest, mainly at the G2/M phase, with a distinctive polo “ring” of chromosomes [17]. These inhibitors target the ATP-binding site of PLK1 and prevent its activity, leading to mitotic arrest and cell death [18]. Further, PLK1 is involved in DNA replication in addition to its role in cell cycle regulation [19]. The targeting of PLK1 is regarded as a very promising strategy for cancer treatment, as several inhibitors of this protein have demonstrated promising outcomes in clinical trials [20].

It is noteworthy that both BRD4 and PLK1 play important roles in mitosis [17], as well as the finding that inhibition of BRD4 and PLK1 can sensitize cancer cells to DNA-damaging agents such as radiation and chemotherapy drugs [21,22]. The simultaneous inhibition of BRD4 and PLK1 enzymes by a single small molecule has shown a synergistic effect in the treatment of several types of cancers by increasing apoptosis and decreasing cancer cell proliferation with reduced toxicity [23]. This is attributed to their ability to disrupt the cell cycle, which causes mitotic arrest, leading to cell death via their kinase inhibitory activity, alongside their impact on the expression of oncogenes and tumor suppressors via their epigenetic inhibitory activity [24]. Preclinical studies have shown promising results for dual BRD4 and PLK1 inhibitors in treating a variety of cancer types. In animal models, these medications have been shown to cause cell death and inhibit tumor growth. Human clinical trials are currently being conducted to assess both the safety and effectiveness of dual BRD4 and PLK1 inhibitors [25]. Overall, targeting both BRD4 and PLK1 represents a promising strategy for developing novel anticancer therapies, because this dual inhibitor can inhibit multiple processes involving cancer growth and survival.

**Figure 1:**
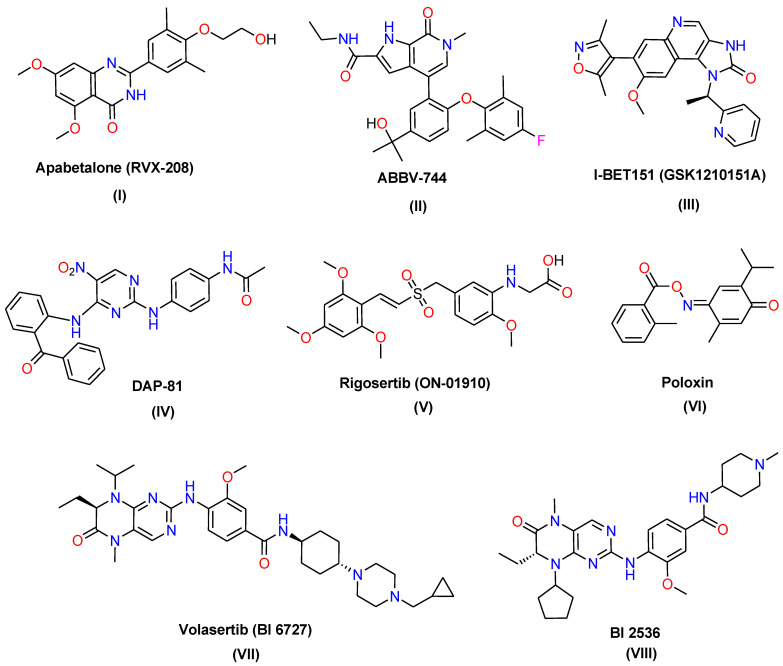
Some reported inhibitors targeting BRD4 and PLK1.

Structurally, diverse cores of BRD4 inhibitors have exhibited potent anticancer activity against various types of cancers in both preclinical studies and clinical trials. These cores encompass quinazoline (Apabetalone or RVX-208 in phase II) (**1**) [26], pyrrolopyridine (ABBV-744 in phase I) (**II**) [27], and imidazoquinoline (I-BET151 or GSK1210151A) (**III**) [28]. On the other hand, inhibitors of PLK1 showed strong inhibitory effects against different cancer types both in vitro and in vivo, as well as in clinical trials. Some of these inhibitors include DAP-81, diaminopyrimidine (**IV**) [29], Rigosertib (ON-01910 in phase III), benzyl styryl sulfone (**V**) [30], and Poloxin, benzoyl oxime cyclohexadiene (**VI**) [31]. Volasertib (BI 6727 in phase III) (**VII**) [32,33] and BI-2536 in phase II (**VIII**) [34] have been identified as potent dual inhibitors of BRD4 and PLK1. Both are experimental drugs with a tetrahydropteridine core (Figure 1).

Pyrimidines are essential building blocks of DNA and RNA, and their inhibition can lead to the disruption of DNA synthesis and cell division, resulting in cell death. Several pyrimidine derivatives have been developed as anticancer agents, including fluoropyrimidines (5-fluorouracil, capecitabine), cytarabine, gemcitabine, and pemetrexed. Such drugs are widely used in the treatment of different cancer types, including colorectal, breast, pancreatic, and lung cancers [35,36]. Furthermore, Schiff bases have been reported in several studies as anticancer agents against a variety of cancer cell lines, such as lung, breast, prostate, and colon cancer. The mechanism of action of Schiff bases involves the induction of apoptosis in cancer cells, inhibition of cell proliferation, and disruption of DNA synthesis. Additionally, it has been demonstrated that Schiff bases have synergistic effects when combined with other chemotherapeutic drugs like cisplatin and doxorubicin. This implies that Schiff bases have potential as adjuvant therapy in cancer treatment [37,38]. Moreover, bromine-based compounds have shown promising results as potential anticancer agents due to their ability to interact with cancer cells and induce cell death. Several studies have shown that brominated compounds exhibit potent anticancer activity via various action mechanisms involving cell proliferation inhibition, apoptosis induction, and angiogenesis inhibition. For example, brominated indoles have been found to inhibit the growth of various cancer cells by inducing apoptosis and inhibiting angiogenesis [39]. In addition, 5-bromo-2′-deoxyuridine (BrdU) is a commonly used anticancer agent that inhibits DNA synthesis by incorporating it into DNA during replication. This leads to cell cycle arrest and ultimately apoptosis in cancer cells [40]. Additionally, bromopyruvic acid (BPA) exhibited broad anticancer activity via inducing DNA breaks [41].

Based on the information previously mentioned, our research endeavor aimed to synthesize dual inhibitors of BRD4 and PLK1, comprising 5,6-aminouracils/thiouracils attached to different hydrophobic moieties via an imino linker comprising bromine groups, while keeping features known to be important in BRD4 and PLK1. Furthermore, our objectives were extended to encompass the synthesis of pteridine derivatives, since pteridine-based compounds have shown promising anticancer results against various cancer types in preclinical studies, as well as in early clinical trials for the treatment of hematological malignancies as dual inhibitors of BRD4 and PLK1 [32,33,34].

### Rationale Strategy for Designing the New Targets

**Figure 2:**
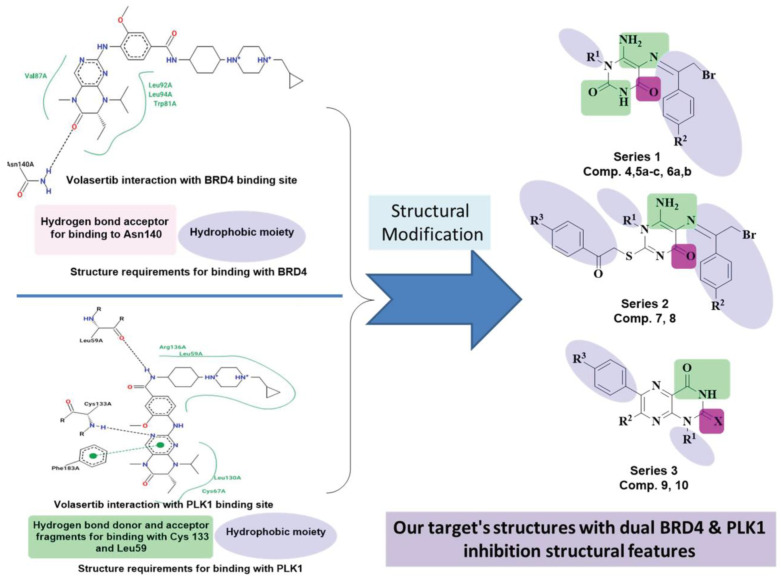
Design rationale and work goal.

It was reported that volasertib can bind at the BRD4 binding site, forming a hydrogen bond interaction with the key amino acid Asn140 through its carbonyl group. The rest of the interactions were hydrophobic in nature through its amino pyrimidine moiety [42]. On the other hand, volasertib binds to PLK1, forming two H-bond interactions with Cys133 and Leu59, and hydrophobic interactions [43]. To fulfill the aforementioned reported binding interactions, structure-based design and structural modifications of known active cores were used to acquire three series of 5-aryl ethylidene aminopyrimidine-2,4-diones, 5-aryl ethylidene amino-2-substitutedthiopyrimidine-4-ones, and 6-aryl pteridines as dual BRD4/PLK1 inhibitors. Firstly, the amino pyrimidine scaffold was maintained in Series 1 and 2, whereas the pteridine backbone was preserved in the third series for hydrogen bonding with the essential amino acids Cys133 and Leu59 in the PLK1 binding site as well as hydrophobic interactions with the protein binding sites. Secondly, we considered keeping the carbonyl group in the three series, which is essential for hydrogen bonding interactions with the key amino acid Asn140 in the BRD4 pocket. Thirdly, the aliphatic hydrophobic chain was modified through introducing a substituted/unsubstituted aryl bromo ethylidene side chain in order to improve hydrophobic interactions at the binding sites. Finally, isopropyl and benzamide fragments were replaced with other fragments, such as benzyl and 2-chlorobenzyl, to study their effect on the binding affinity, where a halo benzyl moiety was reported to improve the inhibitory activity [34]. All of the new series were evaluated for cytotoxicity against breast cancer cells (MDA-MB-231), colorectal adenocarcinoma cells (HT-29), and renal cancer cells (U-937). The most active cytotoxic agents were assessed to study their inhibitory activity on BRD4 and PLK1 enzymes, followed by detection of cell cycle analysis and apoptosis induction, in addition to apoptotic and anti-apoptotic markers (caspase-3, BAX, and Bcl2). Finally, computational (in silico*)* studies (molecular docking simulation, drug-likeness, and ADME profiles studies) were performed to study the mode of binding at the active site of BRD4/PLK1 in order to establish their possibility as potential anticancer candidates (Figure 2).

## 2. Results and Discussion

### 2.1. Chemistry

**Scheme 1:**
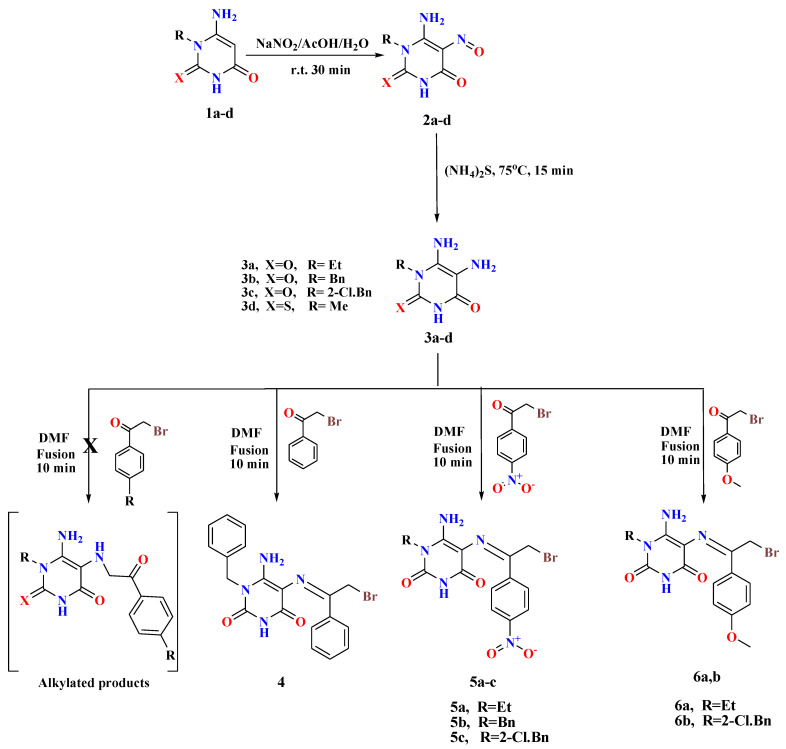
Synthetic pathway of compounds **4**, **5a**–**c**, and **6a**,**b**.

**Scheme 2:**
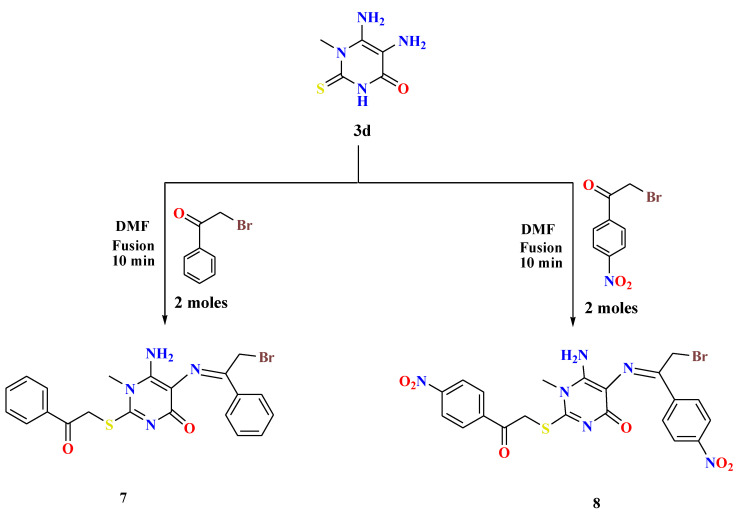
Synthetic route of compounds **7** and **8**.

**Scheme 3:**
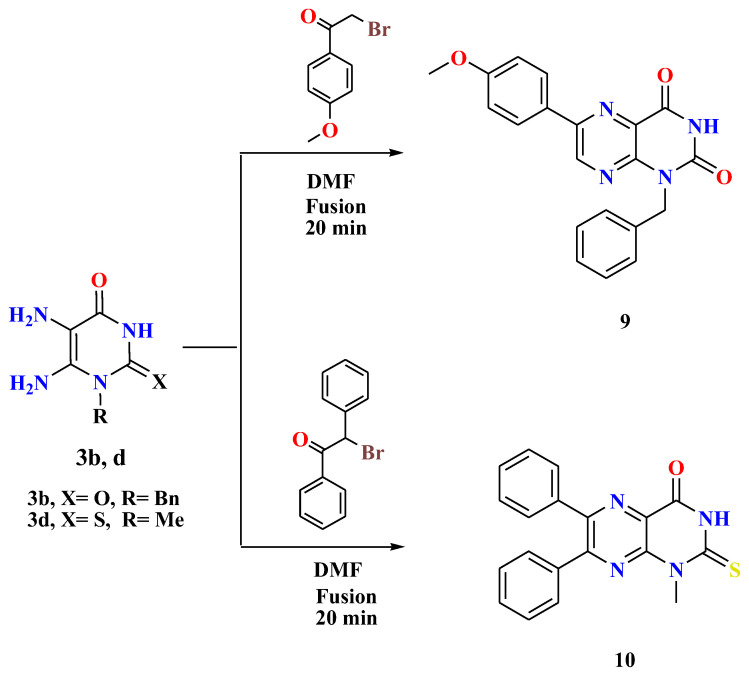
Synthetic pathway of pteridines **9** and **10**.

5,6-Diaminouracils/thiouracils play an important role in the synthesis of valuable heterocycles with high medicinal activity. Herein, 5,6-diaminouracils/thiouracils were used as a backbone to synthesize various imine compounds, **4**, **5a**–**c**, **6a**,**b**, **7**, and **8**, as illustrated in Scheme 1 and Scheme 2. Then, pteridine compounds **9** and **10**, which have important applications in both medicine and biotechnology, were synthesized using a simple and efficient method, as displayed in Scheme 3. The starting materials used, 5,6-diaminouracils/thiouracils **3a**–**d**, were constructed using our previously reported procedures [44,45,46], which involved the nitrosation of 6-aminouracil **1a**–**d** in a stirred solution of sodium nitrite in glacial acetic acid at room temperature, yielding **2a**–**d**. Finally, ammonium sulfide was used as a reducing agent to reduce the latter compounds, which provided our target backbone.

The reaction of a nucleophilic amino group with phenacyl bromide may proceed either through a condensation reaction, yielding an imine product, or an alkylation reaction via the S_N_2 mechanism, yielding an alkylating product. The reaction pathway is determined by the reaction condition; the S_N_2 reaction preferentially proceeds in the basic condition, neutralizing the departing HBr, whereas the condensation pathway preferentially proceeds in the thermal condition without using the base. Based on the literature, the reaction of compounds containing primary amine or 1,2-diamine functionalities with phenacyl halides is typically carried out at reflux temperature in the presence of a base such as triethylamine or potassium acetate, either in a protic solvent, such as ethanol and water [47,48], or in an aprotic solvent, such as *N,N*-dimethylformamide [49,50]. Such reaction conditions initially resulted in the alkylation of the amino group, via S_N_2, which can then undergo further cyclocondensation [47,48,49,50]. In the current study, we used the fusion technique in the presence of a few drops of *N,N*-dimethylformamide for a short time period without using any base catalyst, and this technique led to the formation of imine compounds, where the condensation reaction took place first rather than the alkylation. Accordingly, 6-amino-1-alkyl-5-((2-bromo-1-arylethylidene)amino)pyrimidine-2,4(1*H*,3*H*)-diones **4**, **5a**–**c** and **6a**,**b** were obtained through the fusion of 5,6-diaminouracil derivatives **3a**–**c** with various *α*-bromoacetophenones, including phenacyl bromide, *p*-nitrophenacyl bromide, and *p*-methoxyphenacyl bromide, in the presence of a few drops of DMF for a short time (10 min only), as depicted in Scheme 1. The obtained compounds were fully verified by spectral data and elemental analysis. The ^1^H NMR spectra of compounds **4**, **5a**–**c**, and **6a**,**b** revealed a D_2_O exchangeable singlet signal attributable to uracil C6-NH_2_ in the range between *δ* 7.76 and 8.94 ppm, in contrast to their precursor, confirming the occurrence of condensation. They also showed a second D_2_O exchangeable singlet due to uracil endocyclic NH, around *δ* 10.52–12.04 ppm. Additionally, singlet signals assigned for active methylene protons attached to bromide groups were observed in the *δ* 5.17–5.61 ppm range. These signals were further confirmed by the appearance of their carbon signals in ^13^C NMR spectra at *δ* 61.58–66.64 ppm. In addition, the ^13^C NMR spectra showed signals corresponding to two amidic carbonyl carbons, with no signal referring to ketonic carbonyl carbon regarding phenacyl bromide derivatives, indicating its involvement in the condensation reaction and, thus, confirming the proposed structures. Furthermore, the mass spectra of the synthesized compounds revealed molecular weights that were consistent with the proposed structures, as well as confirming the presence of bromine atoms via the presence of (M**^+.^**+2) peaks in a ratio of 1:1, whereas compounds **5c** and **6b** containing both chlorine and bromine atoms were confirmed by the presence of (M**^+.^**+4) peaks.

To synthesize the rationalized S-alkylated pyrimidin-4-one derivatives **7** and **8**, as well as to chemically confirm the formation of the former synthesized compounds **4**, **5a**–**c**, and **6a**,**b**, the substrate, 5,6-diamino-1-methylthiouracil **3d**, was reacted with double the amount of phenacyl bromide and *p*-nitrophenacyl bromide in the presence of a few drops of DMF under fusion conditions for 10 min (Scheme 2). It was assumed that the initial condensation reaction involving the nucleophilic attack of uracil C5-NH_2_ on the electrophilic carbonyl functionality of phenacyl bromide derivatives was followed by the alkylation of the thiol group by losing the hydrogen bromide molecule, affording the target 6-amino-5-((2-bromo-1-arylethylidene)amino)-1-methyl-2-((2-oxo-2-arylethyl)thio)pyrimidin-4(1*H*)-ones **7** and **8**. The proposed structure of these compounds was completely supported by the spectroscopic data and elemental analyses. The signals of C5-NH_2_ and endocyclic NH vanished from the ^1^H NMR spectra of compounds **7** and **8**, leaving only one singlet signal that was exchanged by D_2_O due to uracil C6-NH_2_ protons. Additionally, two singlet signals in the ranges of *δ* 5.63–5.68 ppm and *δ* 5.04–5.10 ppm, assigned to S-CH_2_ and CH_2_-Br, respectively, were observed. Also, the ^13^C NMR spectra support the existence of two carbon signals corresponding to the two methylene groups, in addition to new ketonic carbonyl carbon signals that occur at their expected frequency. The integration of aromatic protons and their numbers in the ^1^H NMR and ^13^C NMR spectra further confirmed the proposed structures. Furthermore, the mass spectra of compounds **7** and **8** revealed the exact molecular weight of those compounds (M**^+.^**) as well as (M**^+.^**+2) peaks in a ratio of about 1:1, verifying the presence of the bromine atom and, consequently, the proposed chemical structures.

On the other hand, pteridines are heterocyclic compounds with fused pyrimidine and pyrazine ring systems that serve as a building block for many biological molecules, including folate and flavin cofactors, in addition to acting as enzyme inhibitors [51]. Since they are an important area of research in medicinal chemistry and drug discovery, our attempt was expanded to synthesize various 1-alkyl-6-arylpteridines **9** and **10** using a simple and efficient technique (Scheme 3). The rationalized compounds were prepared through the fusion of 5,6-diaminouracil/thiouracil **3b**,**d** and appropriate phenacyl bromides, including phenacyl bromide (2-bromoacetophenone bromide) and 2-bromo-2-phenylacetophenone, in the presence of a few drops of DMF for 20 min. It was noted that increasing reaction time, which was about double that of the previous compounds, led to cyclocondensation via the loss of hydrogen bromide. The mechanism was assumed to involve the initial formation of imine intermediates, followed by S_N_2 reaction and intramolecular cyclization via the elimination of HBr and subsequent oxidation to produce lumazine derivative **9** (pteridine with carbonyl groups at both the C-2 and C-4 positions) and 2-thioxolumazine derivative **10** in good yields. The elucidation of the structures of compounds **9** and **10** was achieved based on their microanalyses and spectral data. Their ^1^H NMR spectra lacked the characteristic signals associated with the uracil C5-NH_2_ and C6-NH_2_ protons, indicating their involvement in the reaction that produced the pteridine derivatives. They also showed an increase in the number of aromatic protons consistent with their structures. Additionally, the ^1^H NMR spectrum of compound **9** displayed a singlet signal corresponding to pyrazine H at *δ* 9.13 ppm, which was also detected in ^13^C NMR at *δ* 148.24 ppm. Mass spectra of compounds **9** and **10** displayed molecular ion peaks at *m*/*z* 360 and 346, consistent with their accurately calculated masses, whereas their base peaks appeared at *m*/*z* 301 and 307, respectively. It is noteworthy that compound **10** was first synthesized in 1974 [52] through a different method, which included the reaction of 4,5-diamino-3-methyl-6-oxo-2-thioxotetrahydropyrimidine with benzil in a mixture of ethanol and water at reflux temperature for an hour, affording 1-methyl-6,7-diphenyl-2-thioxo-2,3-dihydropteridin-4(1*H*)-one in a 55% yield, with a melting point of 287 °C, which is comparable to the melting point we obtained (286–287 °C). The fusion technique used here is considered to be environmentally friendly and sustainable for the production of various pteridines, due to the use of readily available starting materials, nearly solvent-free conditions, short-time reactions, and good yields. Overall, the reaction of 5,6-diaminouracil with phenacyl bromide derivatives can produce a variety of products depending on the reaction conditions. Temperature and reaction time can have a significant impact on the outcome of this reaction and should be carefully considered when designing synthetic routes.

### 2.2. Biological Evaluation

#### 2.2.1. In vitro Cytotoxicity Screening

All newly synthetic compounds were tested for cytotoxicity using a six-dose MTT colorimetric assay on three cancer cell lines; namely, breast cancer cells (MDA-MB-231), colorectal adenocarcinoma cells (HT-29) and renal cancer cells (U-937). The choice of the cell lines is based on the literature’s discovery [53,54] that these cells have been shown to be sensitive to both BRD4 and PLK1 inhibitors. The cytotoxicity of the tested compounds against the selected cancer cell lines was compared to Methotrexate as a reference drug, and the results are shown in Table 1 as a mean of IC_50_ values. The cytotoxicity results showed excellent-to-good cytotoxic activity against the three cancer cell lines. The MDA-MB-231 breast cancer cell line was found to be the most sensitive to the cytotoxic activity of the synthetic targets, followed by colorectal adenocarcinoma cells (HT-29). However, renal cancer cells (U-937) were discovered to be the least sensitive.

#### 2.2.2. Structure–Activity Relationship

**Figure 3:**
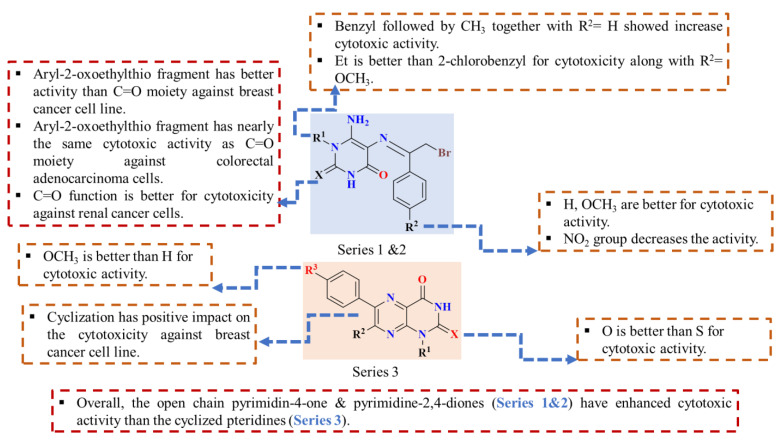
Structure–Activity Relationship of the newly synthesized compounds.

Structurally, the new targets are divided into three series: 5-aryl ethylidene aminopyrimidine-2,4-diones, 5-aryl ethylidene amino-2-substituted thiopyrimidine-4-ones, and 6-ary-l pteridines. Compounds of the first series were found to be the most effective cytotoxic agents. In particular, compounds **4**, **6a**, and **6b** with (R^2^ = H, OCH_3_) showed higher cytotoxicity than Methotrexate. The introduction of an electron-withdrawing group, such as a nitro group on the substituted aryl moiety as in compounds **5a**–**c**, decreased the cytotoxic activity, especially against renal cancer cells. On the other hand, the incorporation of the aryl-2-oxoethylthio fragment at C-2 of the pyrimidine scaffold, as in the second series, showed excellent cytotoxicity against the MDA-MB-231 cell line but revealed no remarkable change in the cytotoxic activity against HT-29 and U-937 cell lines. Consequently, compound **7** (with R^2^ and R^3^ = H) showed strong cytotoxic activity with IC_50_ = 0.4, 0.79, and 1.85 µM against MDA-MB-231, HT-29, and U-937 cell lines, which is nearly more potent or equipotent to Methotrexate with IC_50_ = 2.79, 0.99, and 1.22 µM against MDA-MB-231, HT-29, and U-937 cell lines, respectively. Additionally, the introduction of two nitro groups at the *para* position of the aryl moieties in compound **8** revealed a negative impact on the anticancer activity. Moreover, cyclization of the pyrimidine backbone into the pteridine moiety in Series 3, having a methoxy group at the *para* position of the aryl moiety, as in compound **9**, showed higher cytotoxic activity, only against the MDA-MB-231 cell line (IC_50_ = 2.07 µM), than the Methotrexate (IC_50_ = 2.79 µM), which reflects the importance of the presence of the electron-donating methoxy group. This finding was evidenced by decreasing the cytotoxicity of compound **10** upon replacing the 4-methoxy phenyl moiety with an unsubstituted phenyl ring (Figure 3).

#### 2.2.3. Selectivity Indices

The most active compounds in the MTT cytotoxicity assay were chosen to estimate the in vitro cytotoxicity against normal cells (Vero cells) followed by the detection of selectivity indices for each towards the selected cancer cells, breast cancer cells (MDA-MB-231), colorectal adenocarcinoma cells (HT-29), and renal cancer cells (U-937). In comparison to Methotrexate, the results showed that the majority of the selected derivatives exerted promising selectivity toward the three tumor cells (Table 2). Particularly, the most active compound **7** showed higher selectivity about three-fold (9.8) than Methotrexate (3.64) against breast cancer cells (MDA-MB-231), and one-half (5.0) the selectivity of Methotrexate (10.26) against colorectal adenocarcinoma cells (HT-29), while showing moderate selectivity (2.12) against renal cancer cells (U-937). Moreover, compound **6a** showed selective toxicity (3.27) toward breast cancer cells (MDA-MB-231) that was nearly equivalent to that of Methotrexate (3.64). On the other hand, compound **6b** revealed the least selectivity toward the tested cancer cells.

#### 2.2.4. BRD4 Inhibitory Activity Assay

**Figure 4:**
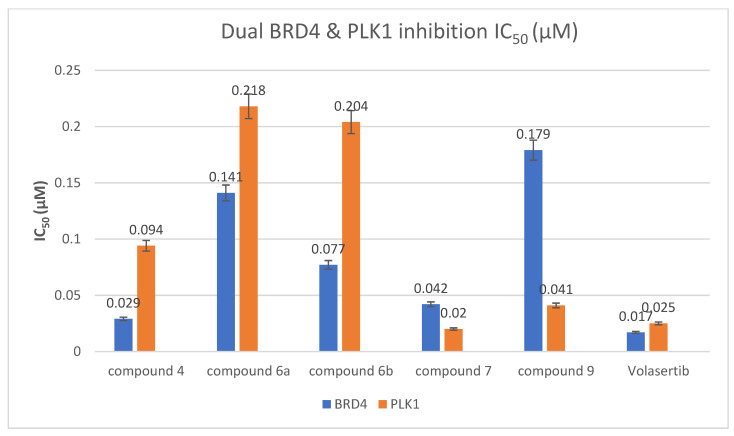
IC_50_ values of compounds **4**, **6a**, **6b**, **7**, and **9**, as well as volasertib, against BRD4/PLK1 enzymes.

‘Epigenetic reader’ BRD4 controls chromatin structure and gene expression by identifying and interacting with acetylated lysine in histones. Because it encourages the expression of the tumor genes, BRD4 has emerged as a therapeutic target for cancer [11]. Hence, the most active compounds in the cytotoxicity assay were estimated to determine their inhibitory activity to BRD4 using volasertib as the reference drug. As presented in Table 3 and Figure 4, compound **4** bearing the unsubstituted phenyl moiety exhibited superior inhibitory activity with an IC_50_ value of 0.029 µM. However, compounds **6a** and **6b** with 4-methoxy phenyl ring showed lower inhibitory activity with IC_50_ values of 0.141 and 0.077 µM, respectively. Moreover, compound **7**, having two unsubstituted phenyl moieties, showed good inhibitory activity with an IC_50_ value of 0.042 µM, which was nearly one-third the inhibitory activity of volasertib with an IC_50_ value of 0.017 µM. On the other hand, lumazine derivative **9** had the least inhibitory action, implying that open-chain pyrimidin-4-one and pyrimidine-2,4-diones were more active than the cyclic pteridine scaffold.

#### 2.2.5. PLK1 Inhibitory Activity Assay

PLK1 is a serine/threonine kinase that plays an important role in multiple phases of the cell cycle [15]. The simultaneous inhibition of PLK1 and BRD4 can result in significant antiproliferative activity. Consequently, the combination of BRD4 and PLK1 inhibitors demonstrated synergistic antitumor effects [55]. As a result, the strongest cytotoxic agents **4**, **6a**, **6b**, **7**, and **9** were studied further for PLK1 enzyme inhibition activity with the goal to identify dual BRD4/PLK1 synergistic targets and compare the results with volasertib as a reference drug. Compound **7**, bearing two unsubstituted phenyl moieties, presented the highest inhibitory activity, being equipotent to volasertib, with an IC_50_ value of 0.02 µM. Additionally, compounds **4** and **9** demonstrated significant inhibition rates (0.094 and 0.041 µM, respectively), which were nearly one-fourth and one-half the activity of volasertib (0.025 µM). The least effective inhibitory compounds were **6a** and **6b** (Table 3 and Figure 4). These outcomes suggested that compounds **4** and **7** might exert their action via inhibition of BRD4/PLK1 enzymes, making them promising candidates for anticancer hybrids. Meanwhile, the pteridine derivative **9** demonstrated considerable anticancer efficacy by inhibiting the PLK1 enzyme solely.

#### 2.2.6. Cell Cycle and Apoptosis Screening

**Figure 5:**
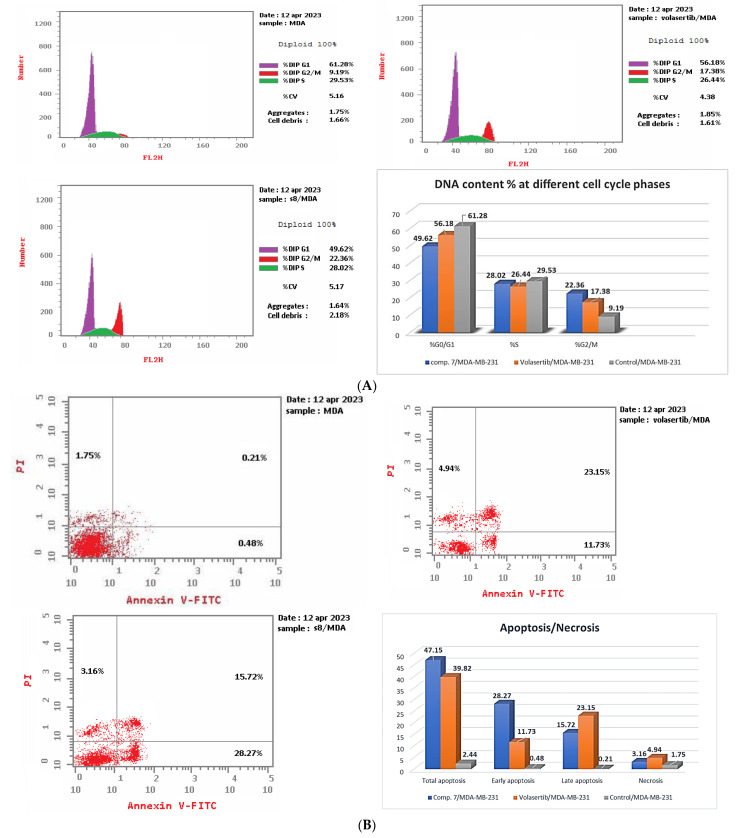
(**A**) Cell cycle analysis of compound **7** (on the down left) and volasertib (on the up right) on MDA-MB-231 cells compared with control cells (on the up left) and graphical representation of the results (on the down right). (**B**) Apoptotic detection of compound **7** (on the down left) and volasertib (on the up right) on MDA-MB-231 cells compared with control cells (on the up left) and graphical representation of the results (on the down right).

Both BRD4 and PLK1 were crucial for the cell cycle; however, inhibiting BRD4 had the potential to stop the cell cycle in the G0/G1 phase, whereas inhibiting PLK1 always resulted in G2/M phase arrest [17,56]. In this study, the most active cytotoxic agent with excellent inhibitory activity of both BRD4/PLK1 enzymes, **7**, was further assayed to determine its mechanism of action, and the results of cell cycle and apoptotic analysis were compared to volasertib as a reference drug. Treatment of MDA-MB-231 cells with compound **7** at its IC_50_ concentration and volasertib showed cell growth arrest at the G2/M phase associated with a strong elevation of the cell count in this stage (from 9.19% to 22.36% for compound **7** and from 9.19% to 17.38% for volasertib). Moreover, there was a slight decrease in the cell percent at the S phase (from 29.53% to 28.02%) as well as a decrease in the cell count at G0/G1 phase (from 61.28% to 49.62%) (Figure 5A). Furthermore, using flow cytometry, the apoptosis induction of MDA-MB-231 cells treated with compound **7** (0.4 µM) and volasertib, and then stained with annexin V/PI, was identified. The results are indicated as a dot plot graph with four quadrant images (Figure 5B). The outcomes showed that compound **7** and volasertib caused remarkable elevation in the late apoptotic stage by about 74.8- and 110.2-fold, respectively, when compared to non-treated control MDA-MB-231 cells. Additionally, both compounds showed a clear increase in early and total apoptosis by 58.8- and 24.4-fold in the early apoptotic stage and 19.3- and 16.3-fold in total apoptosis. However, both compounds also raised the necrotic percent by 1.8- and 2.8-fold, respectively, compared to non-treated cells. Overall, these findings implied that compound **7** could exert anticancer activity by producing cell cycle arrest at the G2/M phase and inducing apoptosis.

#### 2.2.7. Estimation of Apoptotic and Anti-Apoptotic Gene Markers

Since the most effective dual BRD4 and PLK1 inhibitor **7** caused cell cycle arrest at the G2/M stage and apoptosis, its impact on apoptotic genes like BAX and caspase-3 (key executioner of apoptosis), as well as anti-apoptotic genes like Bcl-2 (B-cell lymphoma protein 2), was assessed to provide important proof that **7** stimulated apoptosis. The BIORAD iScript^TM^ One-Step RT-PCR kit with the SYBR^®^ Green system was used in the assessment and the results were compared with the volasertib reference drug. After treating MDA-MB-231 cells with **7** and volasertib for 48 hours, the levels of these genes were estimated, and the outcomes were recorded in Table 4. The tabulated results showed that the pro-apoptotic BAX gene was upregulated, with 7.3-fold more expression than in untreated cells and higher than in the reference drug. As well, there was an apparent increase in caspase-3, showing 3.9-fold more expression than in control cells. On the other hand, the anti-apoptotic marker Bcl-2 is concurrently downregulated and shows 0.30-fold lower than the control. As a result, it was suggested that compound **7** stimulated apoptosis via a variety of mechanisms, including DNA damage and inhibition of mitotic progression through inhibition of mitotic spindle assembly.

#### 2.2.8. Computational Studies

##### Molecular Docking Simulation

**Figure 6:**
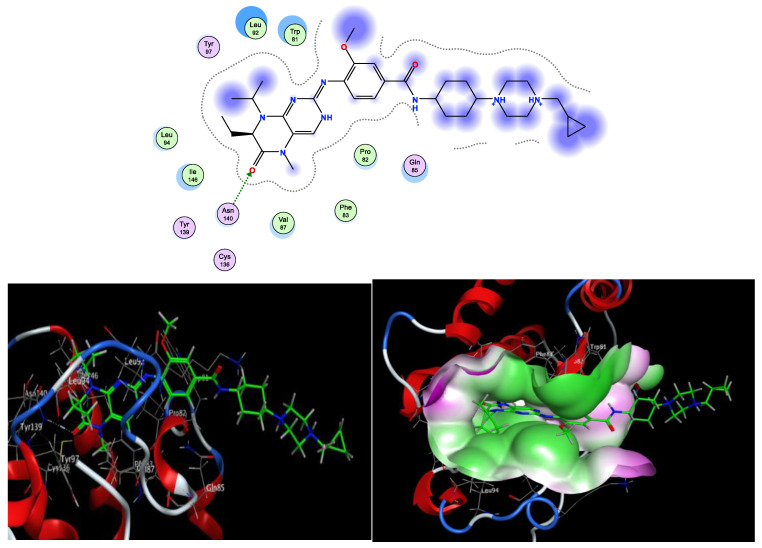
Two- and three-dimensional representations of volasertib (green) at the binding site of BRD4 (PDB ID:5V67).

BRD4 is composed of two binding sites: an Acetyllysine (KAC) binding site and an ATP binding site [57]. Volasertib (BI 6727) is a potent antitumor agent that shows the ability to inhibit BRD4. Volasertib occupies a binding pocket, which is located between the KAC and ATP binding site, forming interaction with the key amino acid Asn140 through its carbonyl group. The rest of the interactions were hydrophobic in nature, with Leu92 and Trp91, as well as Pro82 and Val87, through its pyrimidine-amine moiety. Additionally, volasertib’s benzamide moiety plays a key role in its additional hydrophobic exposure [42]. Figure 6 shows 2D and 3D representations of volasertib at the binding site of BRD4 (PDB ID:5V67) [42].

**Figure 7:**
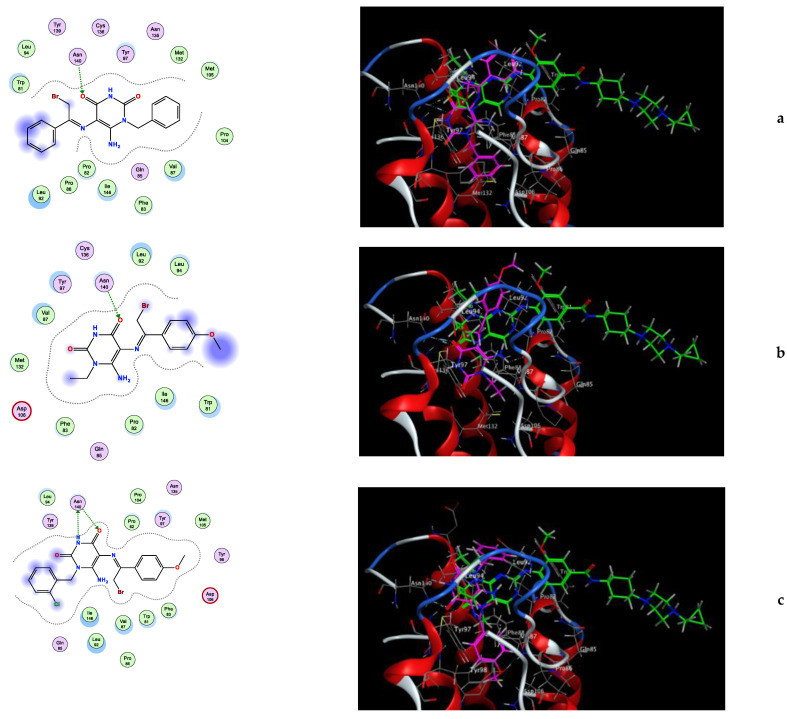

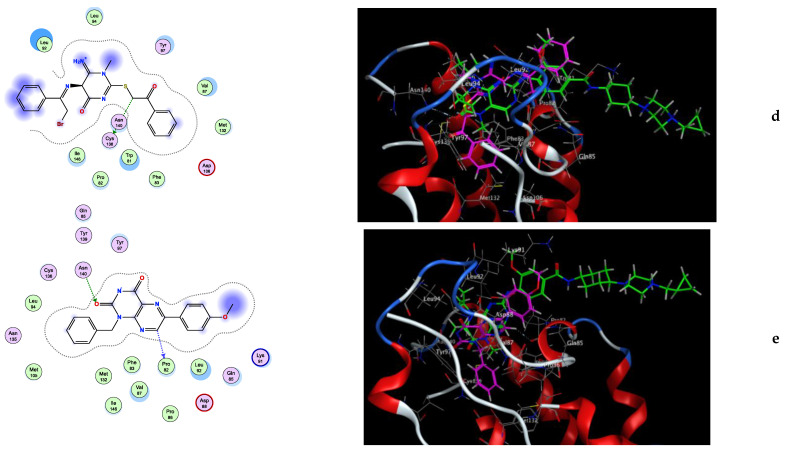
Two-dimensional representations and alignment of compounds **4**, **6a**, **6b**, **7**, and **9** (purple) with volasertib (green) (**a**) **4**, (**b**) **6a**, (**c**) **6b**, (**d**) **7**, and (**e**) **9** at the binding site of BRD4 (PDB ID:5V67).

When docked into the active site of BRD4, compounds **4**, **6a**, **6b**, **7**, and **9** were able to bind in a similar mode to the native ligand. Compound **4** formed an H-bond interaction with the key amino acid Asn140. The pyrimidine-dione was embedded between the two key amino acids Tyr97 and Pro82, forming a hydrophobic interaction. The unsubstituted phenyl ethylidene-amino moiety at position-5 of the pyrimidine ring was buried deep into the pocket between Acetyllysine and ATP binding sites. Compound **7** lacked the H-bond interaction with Asn140 that was compensated by the H-bond interaction with Cys136 as well as hydrophobic interactions with Asn140, Tyr97, and Pro82. The phenyl ethylidene-amino moiety at position-5 of the pyrimidine ring of compound **7** was oriented towards the pocket, unlike that of compounds **6a** and **6b**. Although forming the key H-bond interaction with Asn140, **6a** and **6b** formed only a limited number of hydrophobic interactions. This could be attributed to the presence of bulky 4-methoxy substitution at the phenyl ring of phenyl ethylidene-amino moiety, causing the molecules to acquire opposite orientations in the pocket, limiting their hydrophobic exposure. Compound **9** with a pteridine core showed H-bond interactions with Asn140 and Pro82 with a limited number of hydrophobic interactions. Table 5 shows docking scores and amino acids involved in the interactions for compounds **4**, **6a**, **6b**, **7**, and **9** with the binding site of BRD4 (PDB ID:5V67) compared to the reference ligand volasertib. Figure 7 shows the 2D representations and alignment of compounds **4**, **6a**, **6b**, **7**, and **9** with volasertib at the binding site of BRD4 (PDB ID:5V67).

**Figure 8:**
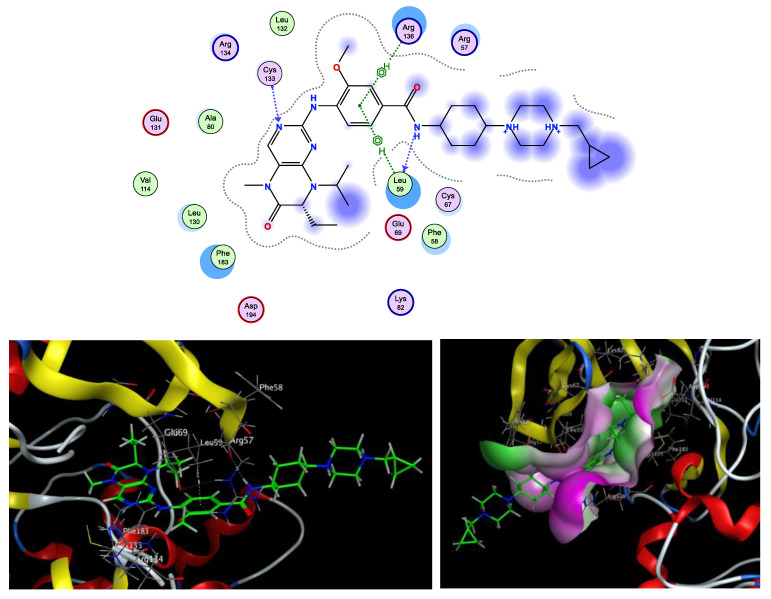
Two- and three-dimensional representations of volasertib (green) at the binding site of PLK1 (PDB ID: 3FC2).

Volasertib can bind to PLK1 at the hinge region of its kinase domain, which is located between amino and carboxylic terminal lobes. Volasertib interacted with the binding site/pocket through two main moieties: a tetrahydropteridin moiety that formed an H-bond interaction with Cys133 at its *N*-3 and an additional hydrophobic interaction with the key amino acid Phe183. The benzamide moiety, on the other hand, formed an H-bond interaction with Leu59 as well as additional Arene–H interactions with Arg136 and Leu59, causing the benzamide moiety to locate itself at the ATP binding pocket of the enzyme [43]. Figure 8 shows volasertib at the binding site of PLK1 (PDB ID: 3FC2) [43].

**Figure 9:**
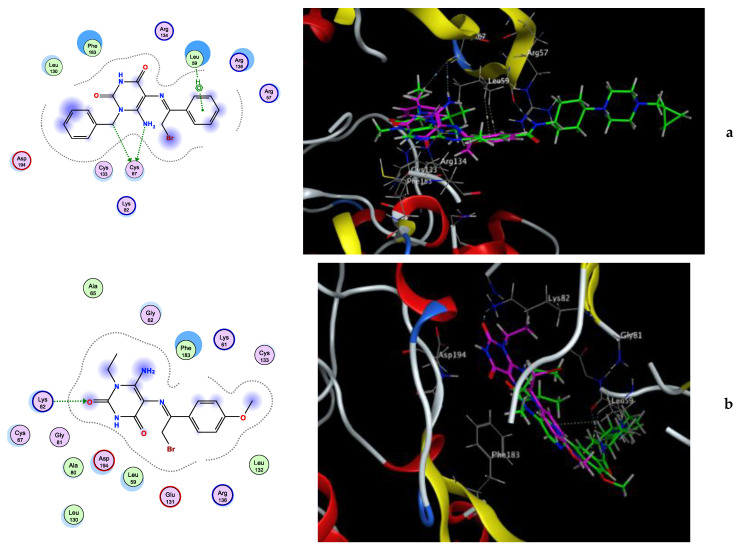

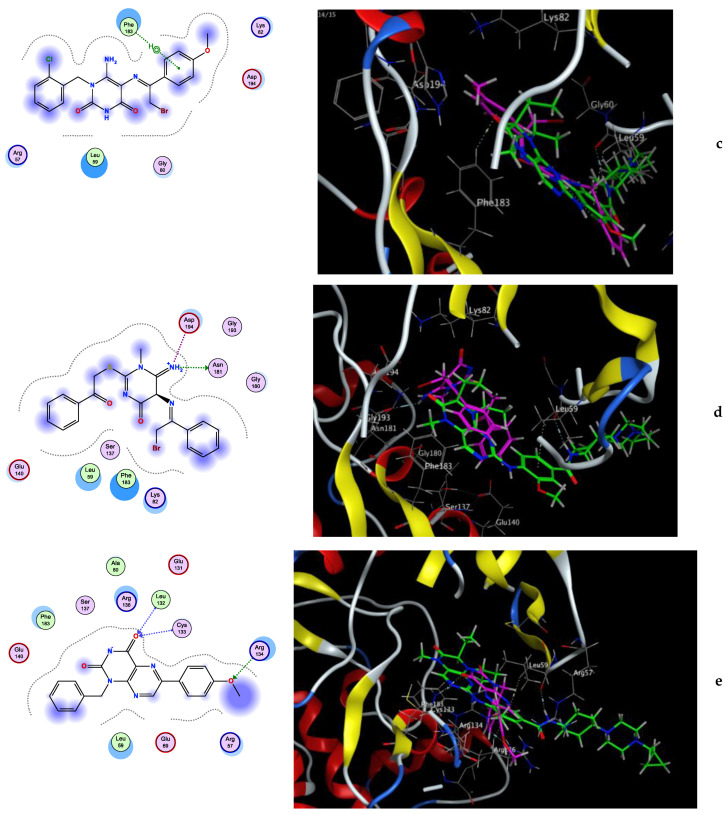
Two-dimensional representations and alignment of compounds **4**, **6a**, **6b**, **7**, and **9** (purple) with volasertib (green) (**a**) **4**, (**b**) **6a**, (**c**) **6b**, (**d**) **7**, and (**e**) **9** at the binding site of PLK1 (PDB ID: 3FC2).

When docked into the active site of PLK1, compounds **4**, **6a**, **6b**, **7**, and **9** showed a similar mode of interaction to the native ligand volasertib. Compound **4** formed a bifurcated H-bond interaction with Cys67 and an Arene-H interaction with Leu59, while it formed hydrophobic interactions with Cys133, Leu59, Phe183, and Cys67. On the other hand, compound **6a** was capable of forming an H-bond interaction with Lys82 and several hydrophobic interactions with Cys133. Compound **6b** lacked the H-bond interaction but formed a large number of hydrophobic interactions with Leu59, Arg57, Phe183, Asp194, and Lys82, together with an Arene-H interaction with Phe183. Compound **7** formed H-bond interactions with Asn181, as well as hydrophobic interactions with Lys82, Leu59, Asp194, Glu140, and Glu180. Also, it maintained the hydrophobic interaction with Phe183, similar to the native ligand, where it π-π stacked with its phenyl ethylidene-amino at Phe183. Compound **9** formed H-bond interactions with the key amino acid Cys133 and other amino acids Leu132 and Arg134. Additionally, it formed hydrophobic interactions with the key amino acids Phe183, Arg134, Arg57, Arg136, and Leu59. Table 6 shows docking scores and amino acids involved in the interactions for compounds **4**, **6a**, **6b**, **7**, and **9** with the binding site of PLK1 (PDB ID: 3FC2) compared to the reference ligand volasertib. Figure 9 shows 2D representations and alignment of compounds **4**, **6a**, **6b**, **7**, and **9** at the binding site of PLK1 (PDB ID: 3FC2).

Compound **7** showed a docking score of −7.89 kcal/mol comparable to that of volasertib (−7.93 kcal/mol) when docked into the active site of BRD4. Structurally, the presence of an unsubstituted aryl bromo ethylidene side chain while maintaining volasertib’s amino pyrimidine without a pteridine core bearing carbonyl group enables compound **7** to form a large number of hydrophobic interactions with key amino acids. Although compound **7** lacked H-bond interaction with Asn140, the interaction was compensated by H-bond interaction with the key amino acid Cys136 through the benzyl moiety. As for PLK1, compound **7** was also capable of binding at the active site in a similar mode and comparable binding energy (−7.93 kcal/mol) to volasertib (−8.79 kcal/mol). The aminopyrimidine core was responsible for H-bond interaction with Asn181, while both benzyl and unsubstituted aryl bromo ethylidene side chains were responsible for hydrophobic interactions with key amino acids at the binding site.

##### The Predicted Drug-Likeness and ADME Properties

The most promising compounds, **4**, **6a**, **6b**, **7**, and **9**, were subjected to the predicted (in silico) bioinformatics study to forecast their physicochemical characteristics and pharmacokinetics and whether they can be oral bioavailable drug candidates in comparison to volasertib. Drug-likeness and ADME properties of compounds were studied using the SwissADME online tool [58]. All of the test compounds revealed excellent physicochemical characteristics and good oral bioavailability in terms of their size (MW between 150 and 500 g/mol), lipophilicity (Log P between −0.7 and +5.0), solubility (Log S not greater than 6), polarity (TPSA between 20 and 140 Å), and flexibility (maximum 10 rotatable bonds). Compounds were found to range from being moderately soluble to soluble, in contrast to the reference volasertib, which is poorly soluble using the applied method [59]. Additionally, all examined compounds obeyed Lipinski’s rule of five [60], which include: M.wt. ≤ 500 g/mol, log P ≤ 5, TPSA ≤ 140 Å, HBA ≤ 10, and HBD ≤ 5 [61]. This outperforms the reference drug volasertib, as shown in Table 7. The molecular weights of compounds ranged from 360.37 to 477.74 g/mol, whereas the volasertib molecular weight exceeded the ideal range (618.81 g/mol). As for the water/octanol partition coefficient, measured as iLog P, all compounds showed an acceptable partition coefficient of orally bioavailable drug candidates <5 [62]. As well, compounds showed TPSA in ideal ranges, where their TPSA values ranged from 89.87 to 115.64 Å^2^. In terms of the number of H-bond acceptor (3-5) and donor (1-2) groups, all compounds followed Lipinski’s rule. Moreover, compounds displayed an acceptable number of rotatable bonds ranging from 4 to 7 bonds, in contrast to volasertib, which showed 11 rotatable bonds. Table 7 depicts the physicochemical properties and drug-likeness of compounds **4**, **6a**, **6b**, **7**, and **9**, compared to volasertib. Furthermore, the ADME descriptors study showed that compounds could be absorbed through the gut wall, where the white of the egg model [63] demonstrates high GI absorption for all compounds. The study revealed that the compounds could not be passively transported through the blood–brain barrier, as displayed by the yolk of the egg model [63], indicating no side effects to the CNS. Finally, compounds had no inhibitory activity toward the liver metabolizing enzyme Cytochrome P450 (CYP2D6), indicating no side effects to the liver. Table 8 compares the findings from the ADME study for compounds **4**, **6a**, **6b**, **7**, and **9** to those from volasertib. Overall, all of the rule principles that could indicate drug-likeness for the investigated compounds **4**, **6a**, **6b**, **7**, and **9** possessed excellent values, which suggests that those substances might fulfill the cell membrane permeability and bioavailability requirements.

## 3. Materials and Methods

### 3.1. Chemistry

Melting points (°C) of all synthesized compounds were determined using a Stuart digital melting point apparatus (SMP 30). Pre-coated (0.25 mm) silica gel plates (Merck 60 F254, Darmstadt, Germany) were used to monitor the reactions, and a UV lamp (254 nm) was used to visualize the spots. The elution systems used were chloroform: methanol (9:1) and ethyl acetate: toluene (1:1). On a Bruker NMR spectrometer (*δ* ppm), NMR spectra were determined in (DMSO) at ^1^H NMR (400 MHz) and ^13^C NMR (100 MHz), using TMS as an internal standard. The Regional Center for Mycology and Biotechnology (RCMB), Al-Azhar University, Egypt, performed mass spectra on the direct inlet part of the mass analyzer in a Thermo Scientific GCMS model ISQ. All chemicals and reagents used were provided by Aldrich Chemicals Co., USA, as well as commercial sources. The starting material **1**–**3** was prepared in accordance with the previously described procedure [46,64,65].

General procedure for the synthesis of 6-amino-1-alkyl-5-((2-bromo-1-arylethylidene) amino)pyrimidine-2,4(1*H*,3*H*)-diones **4**, **5a**–**c**, and **6a**,**b**

An equimolar mixture of 5,6-diaminouracils **3a**–**c** (1.3 mmol) and appropriate phenacyl bromides (1.3 mmol) was heated under fusion in the presence of drops of DMF (0.6 mL) for 10 min. After cooling the reaction mixture, the formed residue was washed with methanol, collected by filtration, and recrystallized from DMF to afford the desired compounds **4**, **5a**–**c**, and **6a**,**b** in good yields.

(*E*)-6-Amino-1-benzyl-5-((2-bromo-1-phenylethylidene)amino)pyrimidine-2,4(1*H*,3*H*)-dione (**4**)
Buff solid, Yield: 56%; m.p. 221–222 °C; ^1^H NMR (400 MHz, DMSO-*d*_6_) *δ* 12.04 (s, 1H, NH uracil), 8.13 (s, 2H, NH_2_), 8.05–8.03 (m, 2H, ArH), 7.48–7.46 (m, 3H, ArH), 7.36 (t, *J* = 7.3 Hz, 2H, ArH), 7.30–7.24 (m, 3H, ArH), 5.50 (s, 2H, CH_2_-ph), 5.23 (s, 2H, CH_2_-Br). ^13^C NMR (100 MHz, DMSO-*d*_6_) *δ* 160.03, 158.99, 154.16, 148.17, 135.84, 134.39, 130.35, 129.27, 128.47, 128.41, 128.36, 127.66, 127.32, 127.16, 126.47, 126.20, 96.87, 65.42, 45.16. MS: *m*/*z* (rel. int. %) = 415 (M^+**.**^+2, 29), 413 (M**^+.^**, 37), 493 (63), 492 (85), 339 (74), 310 (72), 249 (73), 204 (75), 176 (100), 91 (32), 71 (39). Anal. Calcd (%) for C_19_H_17_BrN_4_O_2_ (413.28): C, 55.22; H, 4.15; N, 13.56. Found: C, 55.43; H, 4.26; N, 13.49.
(*E*)-6-Amino-5-((2-bromo-1-(4-nitrophenyl)ethylidene)amino)-1-ethylpyrimidine-2,4(1*H*,3*H*)-dione (**5a**)
Orange solid, Yield: 78%; m.p. 216–218 °C; ^1^H NMR (400 MHz, DMSO-*d*_6_) *δ* 12.04 (s, 1H, NH uracil), 8.57 (s, 2H, NH_2_), 8.33–8.27 (m, 4H, ArH), 5.58 (s, 2H, CH_2_-Br), 3.99 (q, *J* = 7.0 Hz, 2H, CH_2_), 1.17 (t, *J* = 7.0 Hz, 3H, CH_3_). ^13^C NMR (100 MHz, DMSO-*d*_6_) *δ* 162.32, 154.47, 149.45, 148.24, 147.03, 139.73, 127.60, 123.58, 97.57, 66.10, 35.79, 12.17. MS: *m*/*z* (rel. int. %) = 398 (M**^+.^**+2, 45), 396 (M**^+.^**, 51), 378 (27), 376 (25), 360 (83), 358 (77), 355 (72), 351 (100), 302 (98), 290 (50), 288 (68). Anal. Calcd (%) for C_14_H_14_BrN_5_O_4_ (396.20): C, 42.44; H, 3.56; N, 17.68. Found: C, 42.63; H, 3.70; N, 17.92.
(*E*)-6-Amino-1-benzyl-5-((2-bromo-1-(4-nitrophenyl)ethylidene)amino)pyrimidine-2,4(1*H*,3*H*)-dione (**5b**)
Canary yellow solid, Yield: 74%; m.p. 266–268 °C; ^1^H NMR (400 MHz, DMSO-*d*_6_) *δ* 12.11 (s, 1H, NH uracil), 8.46 (d, *J* = 9.0 Hz, 1H, ArH), 8.39 (d, *J* = 9.0 Hz, 1H, ArH), 8.29–8.27 (m, 2H, ArH), 7.93 (s, 2H, NH_2_), 7.45 (d, *J* = 7.3 Hz, 1H, ArH), 7.35–7.32 (m, 2H, ArH), 7.27–7.22 (m, 2H, ArH), 5.47 (s, 2H, CH_2_-ph), 5.21 (s, 2H, CH_2_-Br). ^13^C NMR (100 MHz, DMSO-*d*_6_) *δ* 165.08, 157.17, 156.53, 153.30, 148.63, 140.71, 137.69, 129.26, 129.23, 128.91, 128.78, 128.73, 127.66, 127.50, 126.68, 124.54, 85.49, 62.95, 42.85. MS: *m*/*z* (rel. int. %) = 460 (M**^+.^**+2, 11), 458 (M**^+.^**+14), 429 (39), 397 (36), 254 (30), 232 (35), 230 (37), 98 (65), 96 (100). Anal. Calcd (%) for C_19_H_16_BrN_5_O_4_ (458.27): C, 49.80; H, 3.52; N, 15.28. Found: C, 50.07; H, 3.68; N, 15.43.
(*E*)-6-Amino-5-((2-bromo-1-(4-nitrophenyl)ethylidene)amino)-1-(2-chlorobenzyl) pyrimidine-2,4(1*H*,3*H*)-dione (**5c**)
Orange solid, Yield: 71%; m.p. 235–236 °C; ^1^H NMR (400 MHz, DMSO-*d*_6_) *δ* 10.52 (s, 1H, NH uracil, exchangeable), 8.31-8.25 (m, 4H, ArH), 8.02 (s, 2H, NH_2_, exchangeable), 7.53 (dd, *J* = 7.5, 1.6 Hz, 1H, ArH), 7.33–7.29 (m, 2H, ArH), 6.85–6.81 (m, 1H, ArH), 5.50 (s, 2H, CH_2_-Ar), 5.17 (s, 2H, CH_2_-Br). ^13^C NMR (100 MHz, DMSO-*d*_6_) *δ* 163.07, 157.02, 151.76, 148.54, 147.53, 143.83, 133.95, 132.33, 131.79, 130.30, 130.17, 129.68, 128.22, 127.81, 125.95, 124.45, 76.85, 61.58, 46.68. MS: *m*/*z* (rel. int. %) = 496 (M**^+.^**+4, 7), 494 (M**^+.^**+2, 23), 492 (M**^+.^**, 15), 365 (36), 364 (100), 362 (45), 57 (56). Anal. Calcd (%) for C_19_H_15_BrClN_5_O_4_ (492.71): C, 46.32; H, 3.07; N, 14.21. Found: C, 46.56; H, 3.29; N, 14.42.
(*E*)-6-Amino-5-((2-bromo-1-(4-methoxyphenyl)ethylidene)amino)-1-ethylpyrimidine-2,4(1*H*,3*H*)-dione (**6a**)
Yellow solid, Yield: 55%; m.p. 244–246 °C;^1^H NMR (400 MHz, DMSO-*d*_6_) *δ* 11.82 (s, 1H, NH uracil), 8.94 (s, 2H, NH_2_), 8.06 (d, *J* = 8.9 Hz, 2H, ArH), 7.05 (d, *J* = 8.9 Hz, 2H, ArH), 5.61 (s, 2H, CH_2_-Br), 4.01 (q, *J* = 7.0 Hz, 2H, CH_2_), 3.84 (s, 3H, O-CH_3_), 1.19 (t, *J* = 7.0 Hz, 3H, CH_3_). ^13^C NMR (100 MHz, DMSO-*d*_6_) *δ* 161.85, 161.54, 154.80, 150.28, 147.38, 128.62, 126.21, 114.01, 97.28, 66.64, 55.49, 38.45, 12.01. MS: *m*/*z* (rel. int. %) = 383 (M**^+.^**+2, 14), 381 (M**^+.^**, 20), 359 (56), 346 (94), 315 (71), 314 (100), 277 (60), 76 (86). Anal. Calcd (%) for C_15_H_17_BrN_4_O_3_ (381.23): C, 47.26; H, 4.49; N, 14.70. Found: C, 47.43; H, 4.57; N, 14.86.
(*E*)-6-Amino-5-((2-bromo-1-(4-methoxyphenyl)ethylidene)amino)-1-(2-chlorobenzyl) pyrimidine-2,4(1*H*,3*H*)-dione (**6b**)
Buff solid, Yield: 68%; m.p. 232–234 °C; ^1^H NMR (400 MHz, DMSO-*d*_6_) *δ* 10.53 (s, 1H, NH uracil), 8.00 (d, *J* = 8.9 Hz, 2H, ArH), 7.76 (s, 2H, NH_2_), 7.54–7.51 (m, 1H, ArH), 7.35-7.28 (m, 2H, ArH), 7.00 (d, *J* = 9.0 Hz, 2H, ArH), 6.87–6.84 (m, 1H, ArH), 5.39 (s, 2H, CH_2_-Ar), 5.17 (s, 2H, CH_2_-Br), 3.82 (s, 3H, CH_3_). ^13^C NMR (100 MHz, DMSO-*d*_6_) *δ* 162.06, 160.97, 155.91, 153.90, 150.28, 147.35, 136.33, 131.36, 129.28, 128.58, 127.83, 127.33, 126.61, 125.31, 114.69, 113.71, 97.08, 64.94, 55.27, 44.21. MS: *m*/*z* (rel. int. %) = 481 (M**^+.^**+4, 34), 479 (M**^+.^**+2, 65), 477 (M**^+.^**, 52), 475 (33), 463 (52), 420 (83), 412 (35), 410 (29), 373 (48), 364 (48), 362 (32), 360 (32), 358 (48), 347 (51), 308 (35), 306 (30), 304 (29), 250 (45), 235 (50), 222 (66), 203 (71), 193 (53), 184 (50), 166 (52), 160 (86), 159 (100), 158 (83). Anal. Calcd (%) for C_20_H_18_BrClN_4_O_3_ (477.74): C, 50.28; H, 3.80; N, 11.73. Found: C, 50.44; H, 3.75; N, 11.94.
General procedures for the synthesis of 6-amino-5-((2-bromo-1-arylethylidene)amino)-1-methyl-2-((2-oxo-2-arylethyl)thio)pyrimidin-4(1*H*)-ones (**7** and **8**)
5,6-Diamino-1-methylthiouracil **3d** (1.2 mmol) was fused with double the amount of appropriate phenacyl bromides (2.4 mmol) in the presence of drops of DMF (1 mL) for 10 min. After cooling the reaction mixture, the formed residue was washed with methanol, collected by filtration, and recrystallized from DMF to afford the desired compounds **7** and **8** in interesting yields.
(*E*)-6-Amino-5-((2-bromo-1-phenylethylidene)amino)-1-methyl-2-((2-oxo-2-phenylethyl) thio)pyrimidin-4(1*H*)-one (**7**)
Yellow solid, Yield: 56%; m.p. 229–230 °C;^1^H NMR (400 MHz, DMSO-*d*_6_) *δ* 8.99 (s, 2H, NH_2_), 8.13 (d, *J* = 8.6 Hz, 2H, ArH), 8.08 (d, *J* = 8.6 Hz, 2H, ArH), 7.73 (t, *J* = 7.4 Hz, 1H, ArH), 7.63–7.57 (m, 3H, ArH), 7.52 (t, *J* = 7.4 Hz, 2H, ArH), 5.63 (s, 2H, S-CH_2_), 5.04 (s, 2H, CH_2_-Br), 3.80 (s, 3H, CH_3_). ^13^C NMR (100 MHz, DMSO-*d*_6_) *δ* 191.76, 176.83, 161.84, 156.16, 154.66, 137.85, 135.51, 133.94, 133.19, 132.12, 131.70, 129.32, 128.93, 128.60, 128.41, 127.84, 127.34, 105.40, 66.44, 36.46, 34.48. MS: *m*/*z* (rel. int. %) = 473 (M**^+.^**+2, 27), 471 (M**^+.^**, 30), 345 (40), 342 (92), 291 (39), 273 (38), 235 (100), 145 (39). Anal. Calcd (%) for C_21_H_19_BrN_4_O_2_S (471.37): C, 53.51; H, 4.06; N, 11.89. Found: C, 53.75; H, 4.28; N, 12.11.
(*E*)-6-Amino-5-((2-bromo-1-(4-nitrophenyl)ethylidene)amino)-1-methyl-2-((2-(4-nitro phenyl)-2-oxoethyl)thio)pyrimidin-4(1*H*)-one (**8**)
Brown solid, Yield: 69%; m.p. 214-216 °C; ^1^H NMR (400 MHz, DMSO-*d*_6_) *δ* 8.42–8.37 (m, 5H, ArH), 8.34 (s, 2H, NH_2_), 8.32–8.30 (m, 3H, ArH), 5.68 (s, 2H, S-CH_2_), 5.10 (s, 2H, CH_2_-Br), 3.80 (s, 3H, CH_3_). MS: *m*/*z* (rel. int. %) = 563 (M**^+.^**+2, 23), 561 (M**^+.^**, 28), 501 (46), 593 (54), 463 (79), 457 (47), 455 (45), 407 (93), 400 (98), 397 (85), 395 (86), 390 (97), 377 (85), 212 (52), 211 (100). Anal. Calcd (%) for C_21_H_17_BrN_6_O_6_ S(561.37): C, 44.93; H, 3.05; N, 14.97. Found: C, 45.17; H, 3.24; N, 15.13.
General procedures for the synthesis of 1-alkyl-6-arylpteridines **9** and **10**
A mixture of 5,6-diaminouracil/thiouracil **3b**,**d** (1.2 mmol) and the appropriate phenacyl bromides (1.2 mmol) was heated under fusion in the presence of drops of DMF (0.7 mL) for 20 min. By cooling the reaction mixture, washing the formed residue with methanol, collecting it by filtration, and recrystallizing it from DMF, the desired compounds **9** and **10** were produced in high yields.
1-Benzyl-6-(4-methoxyphenyl)pteridine-2,4(1*H*,3*H*)-dione (**9**)
Orange solid, Yield: 65%; m.p. > 300 °C; ^1^H NMR (400 MHz, DMSO-*d*_6_) *δ* 11.97 (s, 1H, NH uracil), 9.13 (s, 1H, ArH), 8.18 (d, *J* = 8.9 Hz, 2H, ArH), 7.41 (d, *J* = 7.6 Hz, 2H, ArH), 7.30 (t, *J* = 7.6 Hz, 2H, ArH), 7.26–7.21 (m, 1H, ArH), 7.11 (d, *J* = 8.9 Hz, 2H, ArH), 5.41 (s, 2H, CH_2_), 3.84 (s, 3H, O-CH_3_). ^13^C NMR (100 MHz, DMSO-*d*_6_) *δ* 162.08, 159.82, 152.96, 150.35, 148.24, 136.26, 129.47, 128.37, 127.29, 127.09, 126.80, 114.76, 55.53, 44.07. MS: *m*/*z* (rel. int. %) = 360 (M**^+.^**, 68), 348 (56), 347 (57), 341 (80), 309 (53), 308 (57), 301 (100), 298 (46), 272 (82), 256 (88), 77 (58), 46 (79). Anal. Calcd (%) for C_20_H_16_N_4_O_3_ (360.37): C, 66.66; H, 4.48; N, 15.55. Found: C, 66.85; H, 4.60; N, 15.79.
1-Methyl-6,7-diphenyl-2-thioxo-2,3-dihydropteridin-4(1*H*)-one (**10**)
Yellow solid, Yield: 69%; m.p. 286–287 °C; ^1^H NMR (400 MHz, DMSO-*d*_6_) *δ* 12.49 (s, 1H, NH uracil), 8.15 (d, *J* = 7.9 Hz, 2H, ArH), 7.56–7.49 (m, 4H, ArH), 7.45–7.37 (m, 2H, ArH), 7.22–6.91 (m, 2H, ArH), 3.85 (s, 3H, CH_3_). ^13^C NMR (100 MHz, DMSO-*d*_6_) *δ* 173.79, 156.63, 152.40, 150.33, 150.18, 130.57, 129.03, 128.32, 126.53, 111.96, 35.06. MS: *m*/*z* (rel. int. %) = 346 (M**^+.^**, 63), 339 (50), 307 (100), 247 (73), 117 (41), 55 (54). Anal. Calcd (%) for C_19_H_14_N_4_OS (346.41): C, 65.88; H, 4.07; N, 16.17. Found: C, 65.71; H, 4.29; N, 16.40.

### 3.2. Biological Evaluation

#### 3.2.1. In Vitro Cytotoxicity Screening

Using a six-dose MTT colorimetric assay [44,66], all new synthetic compounds were tested for cytotoxicity against three cancer cell lines: MDA-MB-231 breast cancer cells, HT-29 colorectal adenocarcinoma cells, and U-937 renal cancer cells. Additionally, the normal Vero cell line was used to test the most potent compounds **4**, **6a**, **6b**, and **7**. These cell lines were supplied by ATCC (American Type Culture Collection) via the Holding company for biological products and vaccines (VACSERA, Cairo, Egypt). The reference control used was Methotrexate. Detailed descriptions of the MTT colorimetric assay’s protocol can be found in the Supplementary file.

#### 3.2.2. In Vitro BRD4 and PLK1 Enzymes Inhibition Assays

The promising compounds with high cytotoxic activity, **4**, **6a**, **6b**, **7**, and **9**, were screened for their BRD4 and PLK1 inhibitory activity, with volasertib acting as the reference drug. The BRD4 and PLK1 inhibitory assays were carried out in vitro in accordance with the manufacturer’s instructions; BRD4 was assessed using AlphaLISA Bromodomain Assay Protocol [67], and PLK1 kinase was tested using the ADP-Glo™ Kinase Assay Protocol [68], as detailed in the Supplementary file.

#### 3.2.3. Cell Cycle and Apoptosis Screening

The influence of compound **7** and volasertib, the reference drug, on cell cycle phases and its apoptotic potency were investigated using an Ab-139418 propidium iodide flow cytometry kit [69] and an Annexin-V assay and biparametric cytofluorimetric analysis [70], respectively, as instructed by the manufacturer. IC_50_s of both **7** and volasertib were introduced into MDA-MB-231 cells and then incubated for 48 h. An Epics XL-MCL™ Flow Cytometer and Flowing software 2012 were used to count and analyze stained cells. The Supplementary file contains detailed descriptions of the flow cytometric analysis of cell-cycle distribution and cellular apoptosis.

#### 3.2.4. Estimation of Apoptotic and Anti-Apoptotic Gene Markers

The impact of compound **7**, which demonstrated promising dual BRD4 and PLK1 inhibition, on apoptotic genes such as BAX and caspase-3, as well as anti-apoptotic genes such as Bcl-2, was studied using the BIORAD iScript^TM^ One-Step RT-PCR kit with SYBR^®^ Green. MDA-MB-231 cells were cultured before being treated for 48 h with compound **7** and the reference volasertib drug. Fluorometric analysis was used to gauge the level of these genes’ expression. The procedure for the used kit was carried out in accordance with the manufacturer’s instructions [71], which are detailed in the Supplementary file.

### 3.3. Computational Studies

#### 3.3.1. Molecular Docking Simulation

Molecular docking simulation was conducted using the Molecular Operating Environment (MOE) software 2009 following the literature [72]. First, ligands were built using the MOE builder, 3D protonated, partial charges assigned, and then energy-minimized using MMFF94x force field. The protein targets were obtained from the RCSB-Protein Data Bank, and volasertib at the binding site of both BRD4 (PDB ID:5V67) [42] and PLK1 (PDB ID: 3FC2) [43] was used. The proteins were prepared using standard preparation protocol 3D-protonation and automatic correction for atom types and connections followed by and potentially fixed. Finally, the alpha site finder of MOE was used to define active sites, and dummy atoms were generated as alpha spheres to represent polar and hydrophobic components of the active sites of receptors 5V67 and 3FC2. An induced fit docking protocol of MOE was used. Re-docking of the native ligand against the active sites of 5V67 and 3FC2 was executed as standard validation protocol and the cut off of root-mean-square de*via*tion (RMSD) values were <2.0 Å. The interaction of ligands at the active sites was studied while allowing bond rotation and using the triangle matcher approach and London dG scoring function as a 1st scoring function. Five poses of thirty were retained after using the GBVI/WSA dG forcefield-based scoring function as a 2nd scoring function. Poses that had higher S-value and a lower RMSD were recorded.

#### 3.3.2. The Predicted Drug-Likeness and ADME Properties

Computational bioinformatic studies (in silico) were performed on the most promising compounds **4**, **6a**, **6b**, **7**, and **9** to predict their physicochemical properties such as size, lipophilicity, solubility, polarity, and flexibility, as well as pharmacokinetics involving GIT absorption, distribution, and their metabolism by the liver metabolizing enzyme Cytochrome P450. In addition, whether they could be orally bioavailable candidates (following Lipinski’s rule) in comparison to volasertib. SwissADME online software (www.SwissADME.ch/ accessed on 2 May 2023) [58] was used to analyze the compounds’ drug-likeness and ADME properties.

## 4. Conclusions

Based on the structure-based design, novel three series of 5-arylethylidene-aminopyrimidine-2,4-diones **4**, **5a**–**c**, **6a**,**b**, 5-arylethylidene-amino-2-thiopyrimidine-4-ones **7**, **8,** and 6-aryl pteridines **9**, **10** were designed and synthesized as potential dual BRD4 and PLK1 inhibitors. The rationalized compounds were tested for cytotoxicity against breast cancer cells (MDA-MB-231), colorectal adenocarcinoma cells (HT-29), and renal cancer cells (U-937) in comparison to Methotrexate. The cytotoxicity results showed excellent to good cytotoxic activity against three cancer cell lines, where the MDA-MB-231 breast cancer cell line was the most sensitive. The most active compounds **4**, **6a**, **6b**, **7**, and **9** were further estimated to determine their BRD4 and PLK1 inhibitory activity using Volasertib as a reference drug. Compounds **4** and **7** exhibited promising inhibitory activity against both BRD4 and PLK1, with IC_50_ values of 0.029, 0.042 µM and 0.094, 0.02 µM, respectively, which were nearly comparable to Volasertib (IC_50_ = 0.017 and 0.025 µM). Such compounds were chosen to assess their in-vitro cytotoxicity against normal Vero cells, which showed high to moderate selectivity against cancer cell lines. Compound **7** demonstrated high selectivity against breast MDA-MB-231 cancer cell line. Further, compound **7** was found to arrest cell growth at the G2/M phase in a similar mode to Volasertib and caused a 74.8-fold increase in the late apoptotic stage, indicating apoptosis induction. As a result, the impact of compound **7** on apoptotic genes BAX and caspase-3, as well as the anti-apoptotic gene Bcl-2, were studied. Compound **7** stimulated apoptosis via a variety of mechanisms, including an increase in the markers of BAX and caspase-3 and a decrease in the Bcl-2 gene, making it a promising candidate for anticancer drug combination therapy. Furthermore, the computational study of compounds **4**, **6a**, **6b**, **7**, and **9** revealed similar modes of interactions to the native ligand Volasertib when docked into the active sites of both BRD4 and PLK1; additionally, such compounds exerted excellent drug-likeness and pharmacokinetics that outperformed Volasertib.

## Figures and Tables

**Table 1:** IC_50_ of the tested compounds against MDA-MB-231, HT-29, and U-937 cell lines.

Compounds No.	IC_50_ Value (µM) ^a^						
	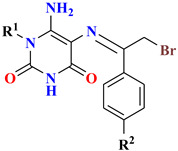						
	R^1^	R^2^	R^3^	X	MDA-MB-231	HT-29	U-937
**4**	Benzyl	H	-	-	0.78 ± 0.01	0.76 ± 0	0.95 ± 0
**5a**	Et-	-NO_2_	-	-	3.24 ± 0.03	3.09 ± 0.01	6.08 ± 0.11
**5b**	Benzyl	-NO_2_	-	-	2.37 ± 0.06	3.66 ± 0.05	6.29 ± 0.26
**5c**	2-Cl-benzyl	-NO_2_	-	-	3.28 ± 0.04	2.24 ± 0.04	6.17 ± 0.15
**6a**	Et-	-OCH_3_	-	-	0.63 ± 0.01	0.75 ± 0.01	0.97 ± 0.01
**6b**	2-Cl-benzyl	-OCH_3_	-	-	1 ± 0.02	1.69 ± 0.01	1.12 ± 0.02
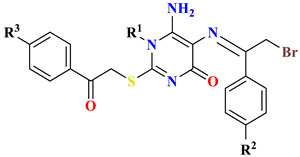							
**7**	-CH_3_	H	H	-	0.4 ± 0.01	0.79 ± 0.01	1.85 ± 0.04
**8**	-CH_3_	-NO_2_	-NO_2_	-	2.31 ± 0.06	6.48 ± 0.08	7.77 ± 0.04
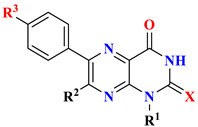							
**9**	Benzyl	H	-OCH_3_	O	2.07 ± 0.03	4.11 ± 0.05	4.42 ± 0.18
**10**	-CH_3_	phenyl	H	S	3.4 ± 0.02	5.63 ± 0.13	8.37 ± 0.23
Methotrexate					2.79 ± 0.06	0.99 ± 0.02	1.22 ± 0.02

^a^ IC_50_ (µM): data presented as the mean of three independent experiments ± SD.

**Table 2:** In vitro cytotoxicity against normal cell and selectivity indices of the most active compounds toward breast cancer cells (MDA-MB-231), colorectal adenocarcinoma cells (HT-29), and renal cancer cells (U-937).

Compounds No.	In Vitro Cytotoxicity against Normal Cells IC_50_ Value (µM) *	Selectivity Indices (SI) **		
	Vero Cells	MDA-MB-231	HT-29	U-937
**4**	1.73 ± 0.03	2.22	2.28	1.82
**6a**	2.06 ± 0.03	3.27	2.75	2.12
**6b**	1.95 ± 0.05	1.95	1.15	1.74
**7**	3.92 ± 0.08	9.8	5.00	2.12
Methotrexate	10.16 ± 0.3	3.64	10.26	8.33

* IC_50_ was detected as the mean of three independent experiments ± SD; ** SI = IC_50_ normal cells/IC_50_ cancer cells; SI: ≥5 (highly selective), >2 (moderately selective), and <2 (non-selective).

**Table 3:** BRD4 and PLK1 inhibition assay of compounds **4**, **6a**, **6b**, **7**, and **9** compared to volasertib reference drug.

Compounds No.	BRD4 * IC_50_ (µM) ± SD	PLK1 * IC_50_ (µM) ± SD
**4**	0.029 ± 0.001	0.094 ± 0.004
**6a**	0.141 ± 0.006	0.218 ± 0.01
**6b**	0.077 ± 0.003	0.204 ± 0.009
**7**	0.042 ± 0.002	0.02 ± 0.001
**9**	0.179 ± 0.008	0.041 ± 0.001
Volasertib	0.017 ± 0.001	0.025 ± 0.001

* IC_50_ was calculated as the mean of three independent experiments ± SD.

**Table 4:** Gene expression of apoptotic and anti-apoptotic genes in MDA-MB-231 cells after treatment with compound **7** and volasertib.

Compound No./Tested Cancer Cell	Apoptotic Genes		Anti-Apoptotic Genes
	BAX	Caspase-3	Bcl-2
7/MDA-MB-231	7.325	3.904	0.301
Volasertib/MDA-MB-231	5.332	5.531	0.351
Control/MDA-MB-231	1	1	1

**Table 5:** Docking scores and amino acids involved in the interactions for compounds **4**, **6a**, **6b**, **7**, and **9** with the binding site of BRD4 (PDB ID:5V67) compared to the reference ligand volasertib.

Compounds	Docking Score (kcal/mol)	Amino Acids H-Bond (Bond Length Å)	Amino Acids Hydrophobic Interactions
**4**	−7.45	Asn140 (2.12)	Tyr97, Val87, Pro82, Leu92, Ile146, Trp81
**6a**	−6.35	Asn140 (2.57)	Tyr97, Val87, Ile146, Trp81, Leu92, Leu94
**6b**	−6.99	Asn140 (2.10, 2.12)	Trp81, Leu94, Ile146, Leu92, Tyr97
**7**	−7.89	Cys136 (3.00)	Asn140, Trp81, Pro82, Ile146, Leu92, Leu94, Try97, Val87
**9**	−5.48	Asn140 (2.08), Pro82 (2.04)	Val87, Leu92, Tyr97, Cys136
Volasertib	−7.93	Asn140 (2.95)	Val87, Pro82, Gln85, Tyr97, Leu92, Trp81, Ile146, Leu94

**Table 6:** Docking scores and amino acids involved in interactions for compounds **4**, **6a**, **6b**, **7**, and **9** with the binding site of PLK1 (PDB ID: 3FC2) compared to the reference ligand volasertib.

Compounds	Docking Score (kcal/mol)	Amino Acids H-Bond (Bond Length Å)	Amino Acids Hydrophobic Interactions
**4**	−6.88	Cys67 (3.11,3.12)	Cys133, Leu59, Phe183, Cys67
**6a**	−5.94	Lys82 (2.20)	Phe183, Leu59, Cys67, Lys82, Leu130, Asp194
**6b**	−6.09	-	Leu59, Arg57, Phe183, Asp194, Lys82
**7**	−7.93	Asn181 (2.26)	Lys82, Phe183, Leu59, Asp194, Glu140, Glu180
**9**	−7.45	Arg134 (2.07), Cys133 (2.14), Leu132 (2.28)	Arg134, Leu59, Arg57, Phe183, Arg136
Volasertib	−8.79	Cys133 (1.86), Leu59 (2.08)	Phe183, Phe59, Leu59, Cys67, Arg136, Arg57

**Table 7:** The predicted physicochemical characteristics and drug-likeness for compounds **4**, **6a**, **6b**, **7**, and **9**, compared to volasertib.

Cpds No.	^a^ M.W. (g/mol)	^b^ iLog P_o/w_	^c^ Log S	^d^ TPSA (Å)	^e^ HBA	^f^ HBD	^g^ NRB	Lipinski Violations
**4**	413.27	2.40	−4.36 **	93.24	3	2	5	0
**6a**	381.22	2.39	−3.36 *	102.47	4	2	5	0
**6b**	477.74	3.23	−5.18 **	102.47	4	2	6	0
**7**	471.37	3.09	−5.99 **	115.64	4	1	7	0
**9**	360.37	2.52	−3.80 *	89.87	5	1	4	0
Volasertib	618.81	5.84	−6.55 ***	106.17	7	2	11	2

^a^ MW, molecular weight; ^b^ Log *P*_o/w_, partition coefficient octanol/water; ^c^ Log S, Aqueous solubility (* soluble, ** moderately soluble, *** poorly soluble); ^d^ TPSA, topological polar surface area; ^e^ HBA, number of H-bond acceptors; ^f^ HBD, number of H-bond donors; ^g^ NRB, number of rotatable bonds.

**Table 8:** The predicted ADME study results for compounds **4**, **6a**, **6b**, **7**, and **9** compared to volasertib.

Cpds No.	BBB Permeant	GI Absorption	Cytochrome P450 (CYP2D6 Inhibitor)
**4**	No	High	No
**6a**	No	High	No
**6b**	No	High	No
**7**	No	High	No
**9**	No	High	No
Volasertib	No	High	No

## Data Availability

Data is contained within the article and supplementary material.

## Supplementary Materials

The following supporting information can be downloaded at: https://www.mdpi.com/article/10.3390/ph16091303/s1, Figures S1–S10: Spectral data; S25–S56: Viability/cytotoxicity Lab Report of **4**, **5a**–**c**, **6a,b**, **7**, **8**, **9**, and **10**, as well as Methotrexate against breast (MDA-MB-231), colorectal (HT-29), and renal (U-937) cancer cells; S57–S62: Cytotoxicity Lab Report of **4**, **6a**, **6b**, and **7** as well as Methotrexate against normal Vero cells; S63: BRD4 Lab Report; S64: PLK1 Lab Report; S65: Gene Expression of **7** (BAX, Caspase-3, and Bcl2).
