# Supplementary material for: Novel Aminopyrimidine-2,4-diones, 2-Thiopyrimidine-4-ones, and 6-Arylpteridines as Dual-Target Inhibitors of BRD4/PLK1: Design, Synthesis, Cytotoxicity, and Computational Studies
Source: Pharmaceuticals (Basel). 2023 Sep 15;16(9):1303. doi: 10.3390/ph16091303 (PMC10535864; doi:10.3390/ph16091303)
Supplement: Supplementary file 1 [file pharmaceuticals-16-01303-s001.zip › pharmaceuticals-2583500-SI.pdf]

Supporting information

Novel Aminopyrimidine-2,4-Diones, 2-Thiopyrimidine-4-ones, and 6-Arylpteridines as Dual-Target Inhibitors of BRD4/PLK1: Design, Synthesis, Cytotoxicity, and Computational Studies

Samar El-Kalyoubi [1,*], Samiha A. El-Sebaey [2,*], Sherin M. Elfeky [3], Hanan A. AL-Ghulikah [4] and Mona S. El-Zoghbi [5]

[1] Department of Pharmaceutical Organic Chemistry, Faculty of Pharmacy, Port Said University, Port Said 42511, Egypt
[2] Department of Pharmaceutical Organic Chemistry, Faculty of Pharmacy (Girls), Al-Azhar University, Youssef Abbas Street, Cairo 11754, Egypt
[3] Department of Pharmaceutical Organic Chemistry, Faculty of Pharmacy, Mansoura University, Mansoura 355516, Egypt; sherin-el-feky@mans.edu.eg
[4] Department of Chemistry, College of Science, Princess Nourah bint Abdulrahman University, P.O. Box 84428, Riyadh 11671, Saudi Arabia; haalghulikah@pnu.edu.sa
[5] Department of Pharmaceutical Chemistry, Faculty of Pharmacy, Menoufia University, Menoufia, Gamal Abd Al-Nasir Street, Shibin-Elkom 32511, Egypt; mona.said@phrm.menofia.edu.eg
* Correspondence: s.elkalyoubi@pharm.psu.edu.eg (S.E.-K.); samiha.ali85@azhar.edu.eg (S.A.E.-S.)

Table of Contents

	Figure No.	Page No.
Biological Evaluation	-	S3-S5
^{1}H NMR, ^{13}C NMR and Mass spectra of ***(E)-6-Amino-1-benzyl-5-((2-bromo-1-phenylethylidene)amino)pyrimidine-2,4(1H,3H)-dione (4)***	**Figure S1**	S6, S7
^{1}H NMR, ^{13}C NMR and Mass spectra of ***(E)-6-Amino-5-((2-bromo-1-(4-nitrophenyl)ethylidene)amino)-1-ethylpyrimidine-2,4(1H,3H)-dione (5a)***	**Figure S2**	S8, S9
^{1}H NMR, ^{13}C NMR and Mass spectra of ***(E)-6-Amino-1-benzyl-5-((2-bromo-1-(4-nitrophenyl)ethylidene)amino)pyrimidine-2,4(1H,3H)-dione (5b)***	**Figure S3**	S10, S11
^{1}H NMR, ^{13}C NMR and Mass spectra of ***(E)-6-Amino-5-((2-bromo-1-(4-nitrophenyl)ethylidene)amino)-1-(2-chlorobenzyl)pyrimidine-2,4(1H,3H)-dione (5c)***	**Figure S4**	S12, S13
^{1}H NMR, ^{13}C NMR and Mass spectra of ***(E)-6-Amino-5-((2-bromo-1-(4-methoxyphenyl)ethylidene)amino)-1-ethylpyrimidine-2,4(1H,3H)-dione (6a)***	**Figure S5**	S14, S15
^{1}H NMR, ^{13}C NMR and Mass spectra of ***(E)-6-Amino-5-((2-bromo-1-(4-methoxy phenyl)ethylidene)amino)-1-(2-chlorobenzyl)pyrimidine-2,4(1H,3H)-dione (6b)***	**Figure S6**	S16, S17
^{1}H NMR, ^{13}C NMR and Mass spectra of ***(E)-6-Amino-5-((2-bromo-1-phenyl ethylidene)amino)-1-methyl-2-((2-oxo-2-phenylethyl) thio)pyrimidin-4(1H)-one (7)***	**Figure S7**	S18, S19
^{1}H NMR, ^{13}C NMR and Mass spectra of ***(E)-6-Amino-5-((2-bromo-1-(4-nitropheny ethylidene)amino)-1-methyl-2-((2-(4-nitro phenyl)-2-oxoethyl)thio)pyrimidin-4(1H)-one (8)***	**Figure S8**	S20
^{1}H NMR, ^{13}C NMR and Mass spectra of ***1-Benzyl-6-(4-methoxyphenyl)pteridine-2,4(1H,3H)-dione (9)***	**Figure S9**	S21, S22
^{1}H NMR, ^{13}C NMR and Mass spectra of ***1-Methyl-6,7-diphenyl-2-thioxo-2,3-dihydropteridin-4(1H)-one (10)***	**Figure S10**	S23, S24
Viability/cytotoxicity Lab Report of **4, 5a-c, 6a,b, 7, 8, 9,** and **10**, as well as Methotrexate against breast (MDA-MB-231), colorectal (HT-29), and renal (U-937) cancer cells	**-**	S25-S56
Cytotoxicity Lab Report of **4, 6a, 6b,** and **7** as well as Methotrexate against normal Vero cells	**-**	S57-S62
BRD4 Lab Report	**-**	S63
PLK1 Lab Report	**-**	S64
Gene Expression of **7** (BAX, Caspase-3, and Bcl2)		S65

1. Biological evaluation

1.1. *In-vitro* cytotoxicity screening

Using a six-dose MTT colorimetric assay [44, 66], all new synthetic compounds were tested for cytotoxicity against three cancer cell lines: MDA-MB-231 breast cancer cells, HT-29 colorectal adenocarcinoma cells, and U-937 renal cancer cells. Additionally, the normal Vero cell line was used to test the most potent compounds **4**, **6a**, **6b**, and **7**. The cell lines were supplied by ATCC (American Type Culture Collection). The reference control used was Methotrexate. The cytotoxic activities of the examined compounds were quantified by inoculating a 96-well tissue culture plate with 1 X 10^5 cells / mL (100 uL/well) and incubating it at 37°C for 24 hours to develop a complete monolayer sheet. After forming a confluent sheet of cells, the growth medium was decanted from 96-well microtiter plates, and the cell monolayer was washed twice with wash media. Two-fold dilutions of tested samples were made in RPMI medium with 2% serum (maintenance medium), and 0.1 mL of each dilution was tested in different wells, with three wells serving as controls and receiving only maintenance medium. Following that, the plate was incubated at 37°C and examined. Cells were investigated for any physical signs of toxicity, such as partial or complete monolayer loss, rounding, shrinkage, or cell granulation. MTT solution was prepared (5mg/ml in PBS) (BIO BASIC CANADA INC), and 20 uL MTT solution was added to each well. After 5 minutes on a shaking table at 150 rpm to thoroughly mix the MTT into the media, it was incubated (37°C, 5% CO_2) for 4 hours to allow the MTT to be metabolized. Formazan (MTT metabolic product) was resuspended in 200 uL DMSO and shaken at 150 rpm for 5 minutes to thoroughly mix the formazan into the solvent. The optical density was measured at 560 nm, and the background was subtracted at 620 nm. Finally, the IC_{50} of the test compound in comparison to the reference was calculated using the GraphPad Prism software.

1.2. *In vitro* BRD4 and PLK1 Enzymes inhibition assay

The promising compounds with high cytotoxic activity, **4, 6a, 6b, 7,** and **9**, have been screened for their BRD4 and PLK1 inhibitory activity, with Volasertib acting as the reference drug. The BRD4 and PLK1 inhibitory assays were carried out *in vitro* in accordance with the manufacturer's instructions; BRD4 was assessed using AlphaLISA Bromodomain Assay Protocol [67], and PLK1 kinase was tested using the ADP-Glo™ Kinase Assay Protocol [68], as shown below.

1.2.1. BRD4 inhibitory activity assay

The master mixture was prepared as follows: N wells × (2.5 µL 3x BRD Homogeneous Assay Buffer 1 + 1 µL BET Bromodomain Ligand + 1.5 µL H_2O). BRD4 (BD2) should be thawed on ice. Simply, the protein-containing tube was spined to recover the full content of the tube after the first thaw. Both proteins should be aliquoted into single-use aliquots. Immediately, any remaining undiluted protein should be stored in aliquots at -80°C. BRD4 (BD2) was diluted in 1x BRD Homogeneous Assay Buffer 1 at 4 ng/µL. The diluted proteins were kept on ice until they were used. 5 µl of the master mixture is added to each well designated for the “Positive Control”, “Test Inhibitor”, and “Blank”. For the “Substrate Control”, 2.5 µL 3x BRD Homogeneous Assay Buffer 1 + 1 µL Non-acetylated Ligand 1 + 1.5 µL H_2O is added. Each “Test Inhibitor” well received 2.5 µL of inhibitor solution, while the “Positive

Control", "Substrate Control", and "Blank" wells received 2.5 μL of the same solution without inhibitor (inhibitor buffer). Then, the "Blank" well is filled with 2.5 μL of 1x BRD Homogeneous Assay Buffer 1. After that, GSH Acceptor beads (PerkinElmer #AL109C) are diluted 250-fold with 1x BRD Homogeneous Detection Buffer 1. Next, 10 μL per well is added, the plate is shaken, and it is incubated at room temperature for 30 minutes. Streptavidin-conjugated donor beads (PE #6760002S) are diluted 250-fold with 1x BRD Homogeneous Detection Buffer 1. Then, 10 μL is added per well and incubated at room temperature for further 15 – 30 minutes [67]. The Alpha counts were read using the EnVision® multimode plate reader with the standard Alpha detection settings.

1.2.2. PLK1 inhibitory activity assay

The kinase reaction was performed with active human recombinant PLK1 and combined with the substrate dephosphorylated casein (positive control) using 1 X reaction buffer (40 mM Tris-HCl pH 7.4, 20 mM $MgCl_2$, 0.1 mg/mL BSA, 1 mM DTT) and 20 μM ATP. The reaction was incubated at 30°C for 45 minutes. The amount of ATP transferred was calculated using Kinase-Glo reagent from Promega according to the manufacturer's instruction [68]. A plate-reading luminometer is used to measure the luminescence.

1.3. Cell cycle and apoptosis screening

The influence of compound **7** and Volasertib, the reference drug, on cell cycle phases and its apoptotic potency were investigated using an Ab-139418 propidium iodide flow cytometry kit [69] and an Annexin-V assay and biparametric cytofluorimetric analysis [70], respectively, as instructed by the manufacturer. IC_{50}s of both **7** and Volasertib were introduced into MDA-MB-231 cells and then incubated for 48 hours. An Epics XL-MCL™ Flow Cytometer and Flowing software were used to count and analyze stained cells. The Supplementary file contains detailed descriptions of the flow cytometric analysis of cell-cycle distribution and cellular apoptosis.

1.3.1. Cell cycle analysis

Compound **7** and Volasertib were applied to the MDA-MB-231 cell culture for 48 h at its IC_{50} concentration. Briefly, a six-well plate containing MDA-MB-231 breast cancer cells was seeded with 1 X 10^5 cells per well and then incubated for 24 hours. IC_{50}s of compound **7** and Volasertib or 0.1% DMSO was applied to the cells for 24 hours. Following that, cells were gathered and fixed in ice-cold 70% ethanol at 4 °C for 12 hours. The cells were then rinsed with cold Phosphate Buffer Saline (PBS) and incubated for 30 minutes at 37°C in 0.5 mL of PBS after the ethanol was removed. Propidium iodide was used to stain the cells for 30 min while they were in the dark. DNA content was determined using a flow cytometer [69].

1.3.2. Apoptosis screening

The MDA-MB-231 cell culture was treated with IC_{50}s of compound **7** and Volasertib for 48 h. Each well of a 6-well plate received 1 X 10^5 conc. breast cancer (MDA-MB-231) cells that were then incubated for 24 hours. The cells were then exposed for 24 h to IC_{50}s of compound **7** and Volasertib or (0.1%) DMSO before being harvested, washed with PBS, and stained for 15 min at room temperature in the dark with annexin V-FITC and PI in binding buffer (10

μM HEPES, 140 μM NaCl, and 2.5 μM $CaCl_2$ at pH 7.4). The flow cytometer was then used to examine the cells [70].

1.4. Estimation of apoptotic and anti-apoptotic gene markers

The impact of compound **7**, which demonstrated promising dual BRD4 and PLK1 inhibition, on apoptotic genes such as Bax and caspase-3, as well as anti-apoptotic genes such as Bcl-2, was studied using the BIORAD iScript™ One-Step RT-PCR kit with SYBR® Green. MDA-MB-231 cells were cultured before being treated for 48 hours with compound **7** and the reference Volasertib drug. Fluorometric analysis was used to gauge the level of these genes' expression. The procedure for the used kit was carried out in accordance with the manufacturer's instructions [71], which are detailed as follows:

1.4.1. RNA isolation and reverse transcription

m RNA isolation is carried out using an RNeasy extraction kit, up to 1 x 10^7 cells, depending on the cell line. Cells are disrupted in RNeasy Lysis Buffer (RLT buffer), homogenized, then ethanol is added to the lysate, creating conditions that promote selective binding of RNA to the RNeasy membrane. The sample is then applied to the RNeasy Mini spin column, and total RNA binds to the membrane, contaminants are efficiently was headway, and high-quality RNA is eluted in RNase-free water.

1.4.2. Master mix preparation

All the following reagents were mixed together to give a total volume (50 μL). 2X SYBR® Green RT-PCR reaction mixture (25 μL), forward primer (10 μM) (1.5 μL), reverse primer (10 μM) (1.5 μL), nuclease-free H2O (11 μL), RNA template (1 pg to 100 ng total RNA) (10 μL) and iScript reverse transcriptase for One-Step RT-PCR (1 μL).

1.4.3. Amplification protocol

Incubate complete reaction mixture in a real-time thermal detection system (Rotorgene) as follows: cDNA synthesis: 10 min at 50 °C, iScript reverse transcriptase inactivation: 5 min at 95 °C, polymerase chain reaction (PCR) cycling and detection (30–45 cycles): 10 s at 95 °C and 30 s at 55 °C to 60 °C (data collection step) and melt curve analysis: 1 min at 95 °C and 1 min at 55 °C and 10 s at 55 °C (80 cycles, increasing each by 0.5 °C each cycle).

Figure S1: ^{1}H NMR, ^{13}C NMR and Mass spectra of **(*E*)-6-Amino-1-benzyl-5-((2-bromo-1-phenylethylidene)amino)pyrimidine-2,4(1*H*,3*H*)-dione (4)**

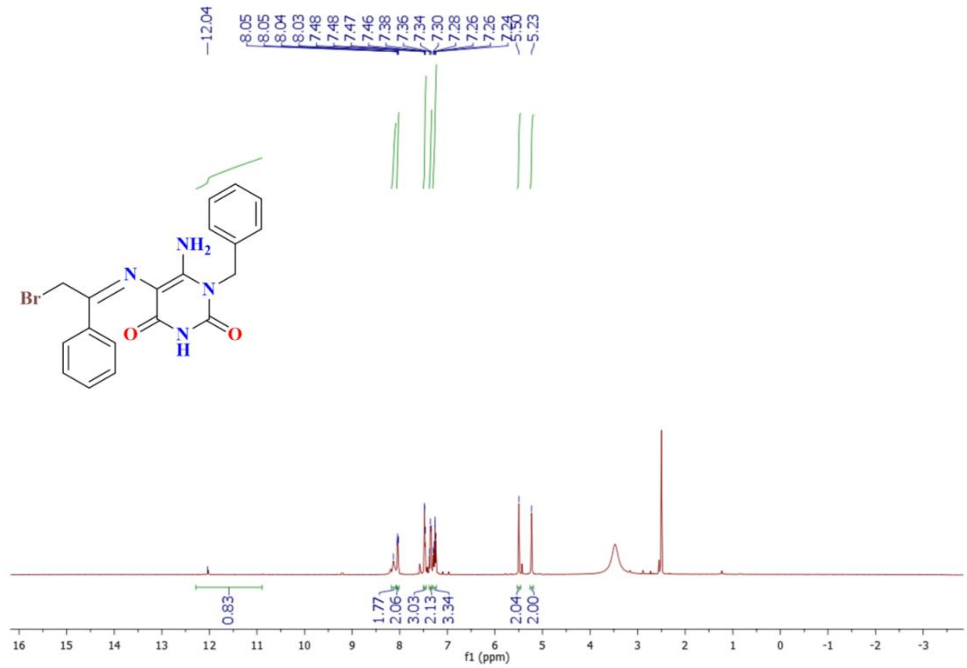

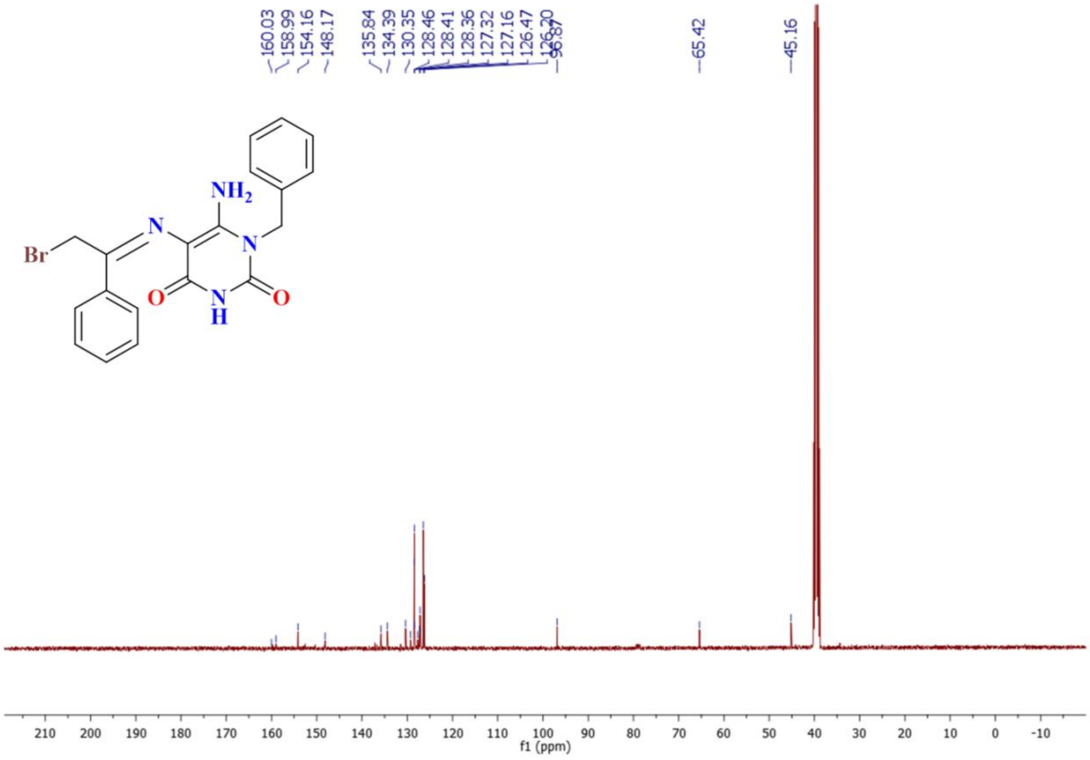

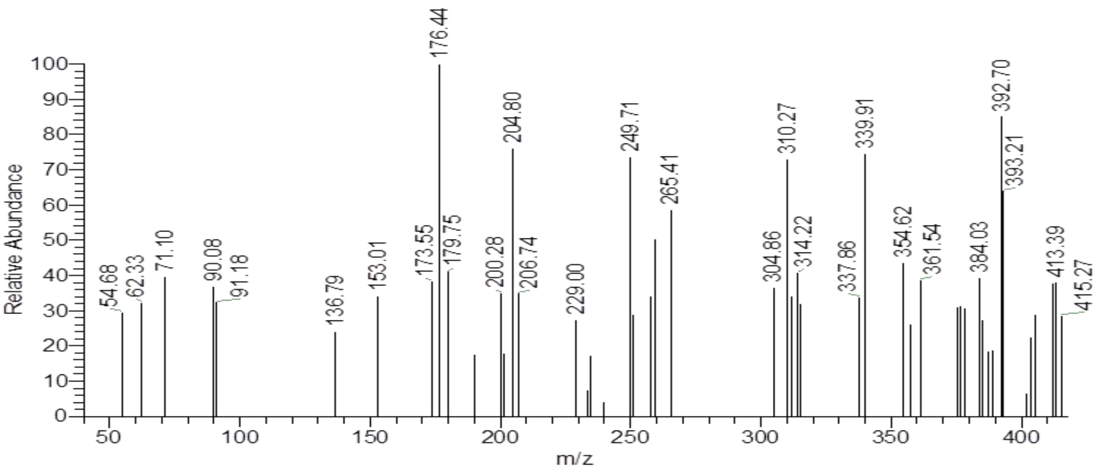

Relative Abundance
m/z
176.44
204.80
249.71
265.41
310.27
339.91
392.70
393.21
54.68
62.33
71.10
90.08
91.18
136.79
153.01
173.55
179.75
200.28
206.74
229.00
304.86
314.22
337.86
354.62
361.54
384.03
413.39
415.27

Figure S2: ^{1}H NMR, ^{13}C NMR and Mass spectra of **(*E*)-6-Amino-5-((2-bromo-1-(4-nitrophenyl)ethylidene)amino)-1-ethylpyrimidine-2,4(1*H*,3*H*)-dione (5a)**

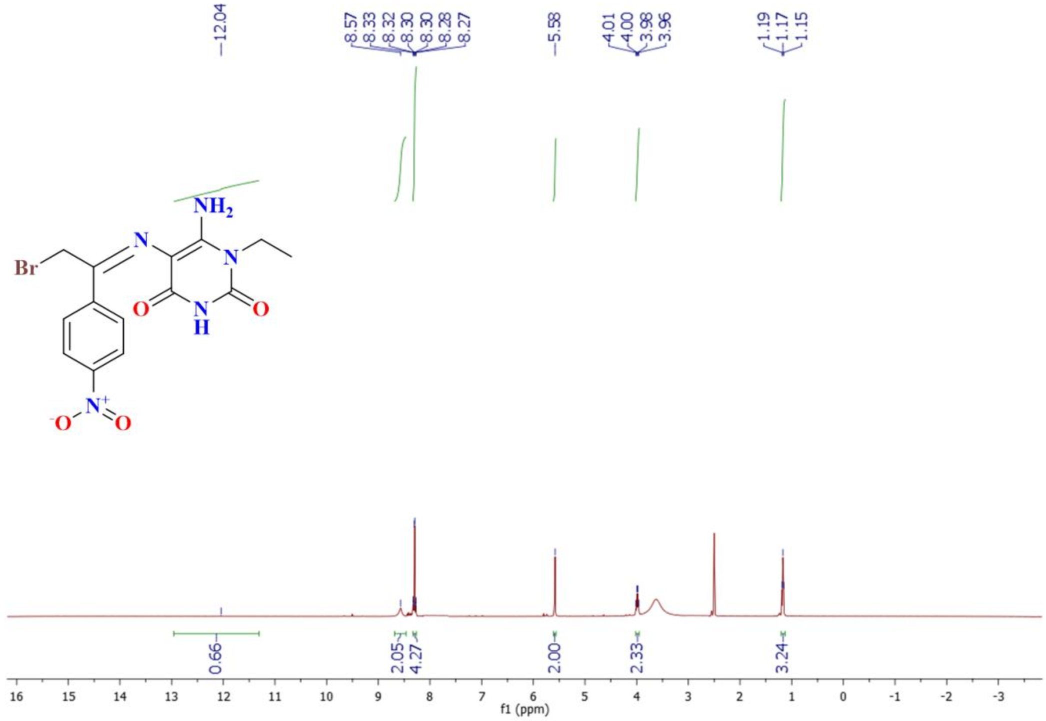

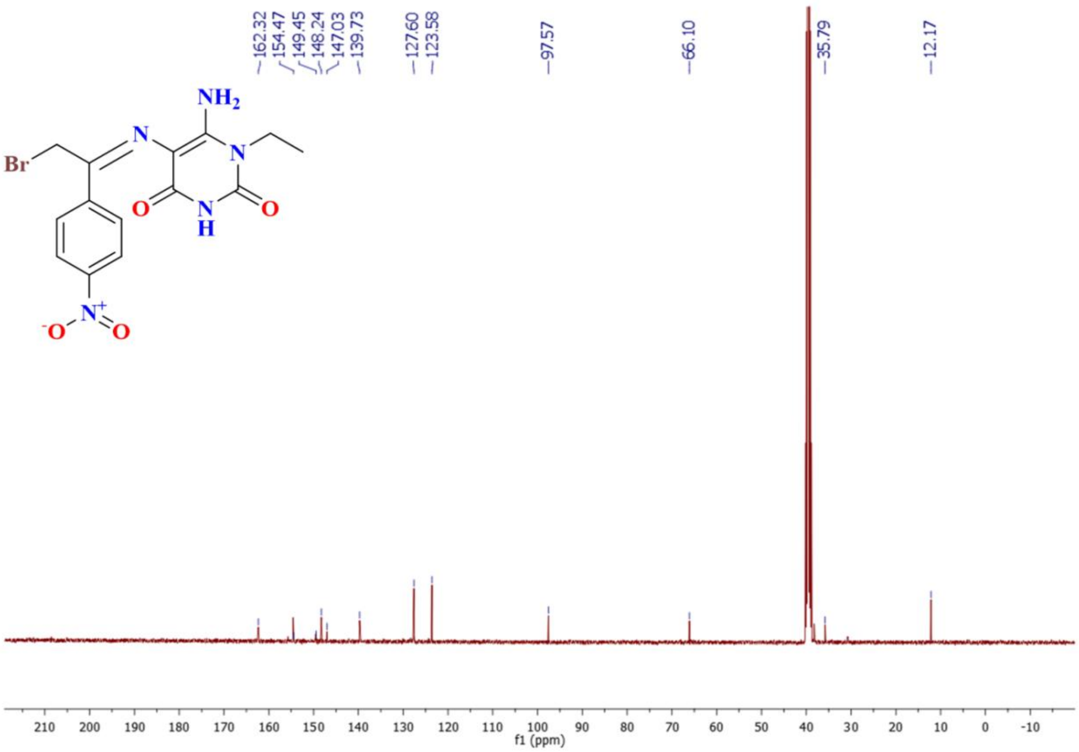

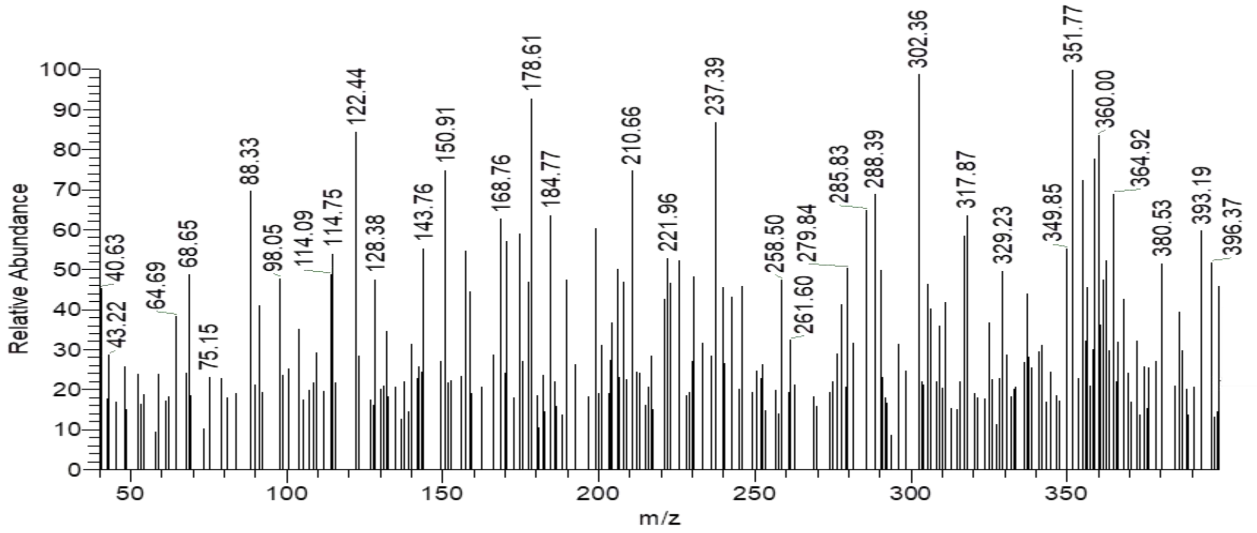
Relative Abundance
m/z
40.63
43.22
64.69
68.65
75.15
88.33
98.05
114.09
114.75
122.44
128.38
143.76
150.91
168.76
178.61
184.77
210.66
221.96
237.39
258.50
261.60
279.84
285.83
288.39
302.36
317.87
329.23
349.85
351.77
360.00
364.92
380.53
393.19
396.37

Figure S3: ^{1}H NMR, ^{13}C NMR and Mass spectra of **(*E*)-6-Amino-1-benzyl-5-((2-bromo-1-(4-nitrophenyl)ethylidene)amino)pyrimidine-2,4(1*H*,3*H*)-dione (5b)**

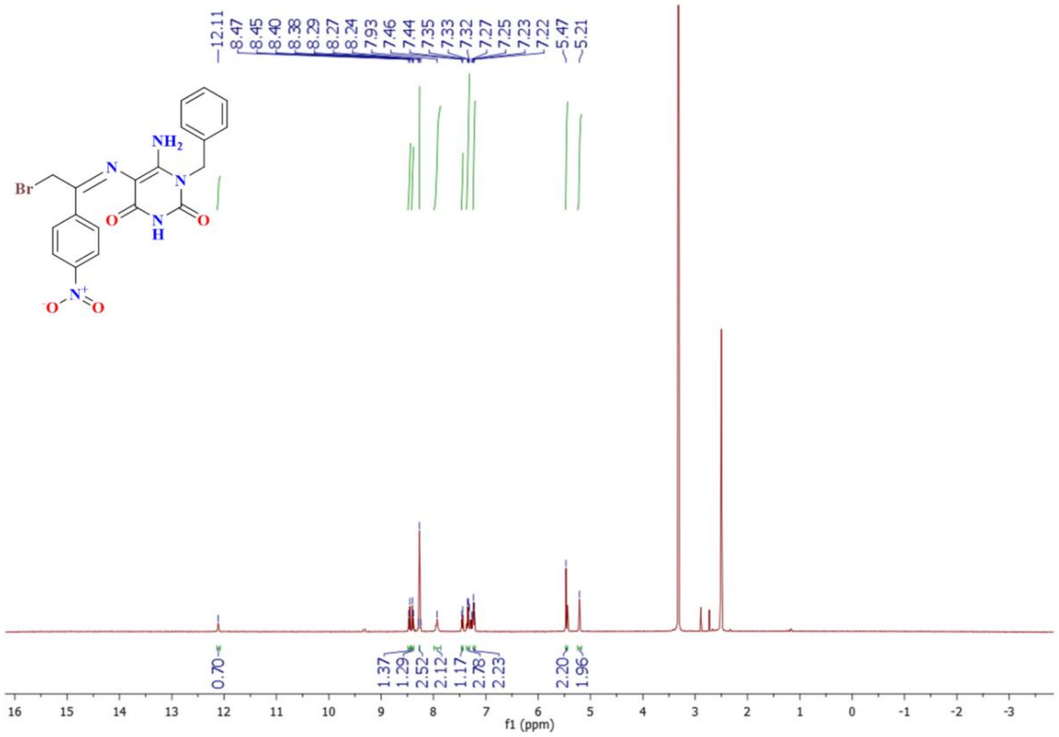

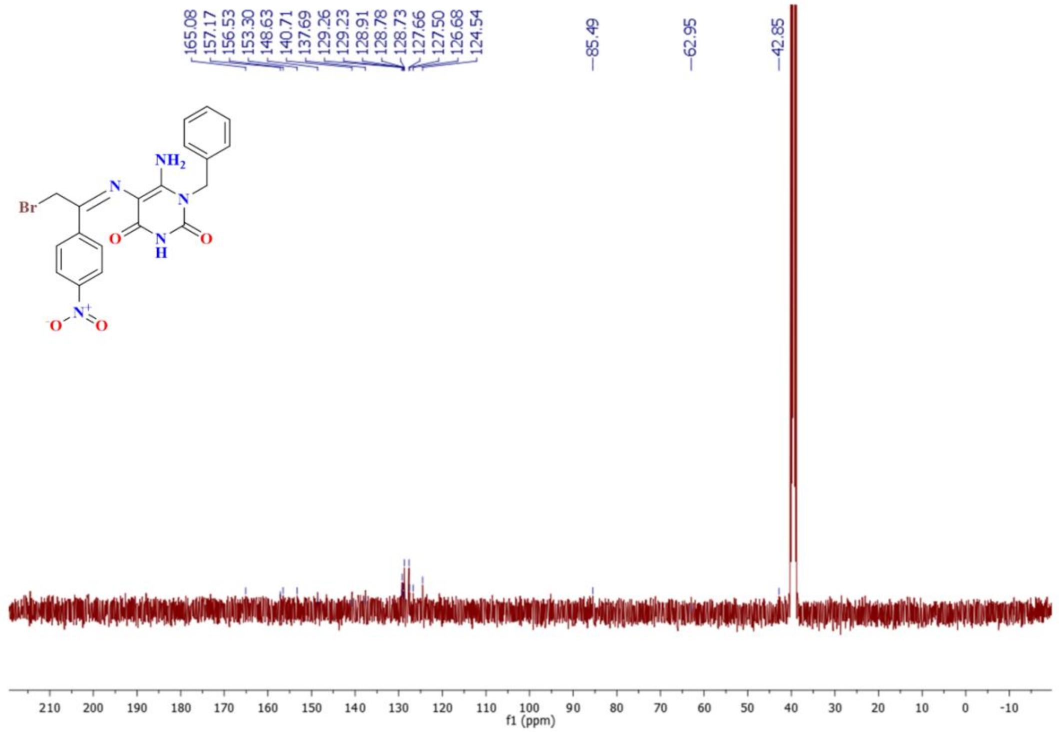

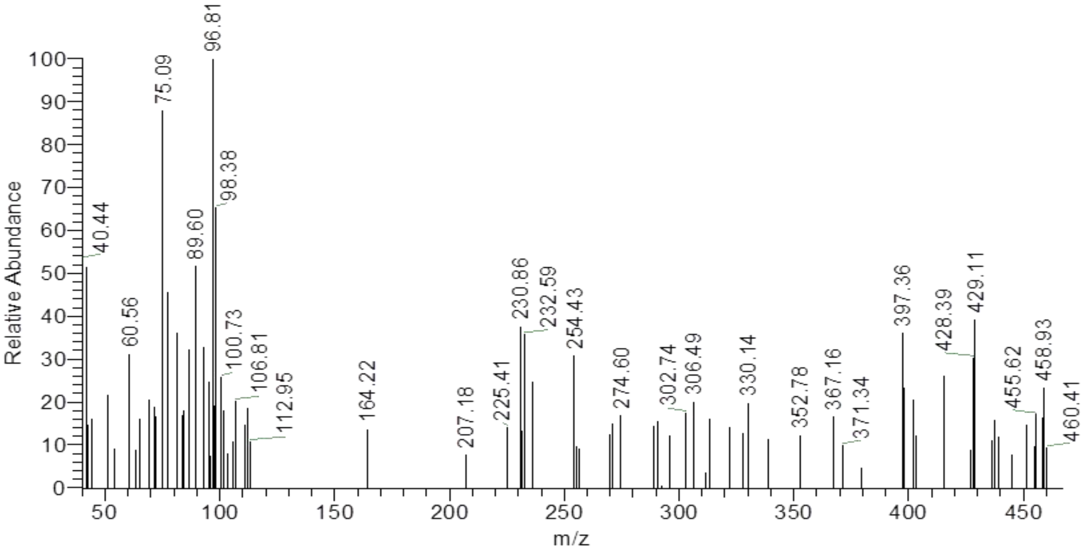

Relative Abundance
m/z
96.81
75.09
98.38
40.44
89.60
60.56
100.73
106.81
112.95
164.22
207.18
225.41
230.86
232.59
254.43
274.60
302.74
306.49
330.14
352.78
367.16
371.34
397.36
428.39
429.11
455.62
458.93
460.41

Figure S4: ^{1}H NMR, ^{13}C NMR and Mass spectra of **(*E*)-6-Amino-5-((2-bromo-1-(4-nitrophenyl)ethylidene)amino)-1-(2-chlorobenzyl)pyrimidine-2,4(1*H*,3*H*)-dione (5c)**

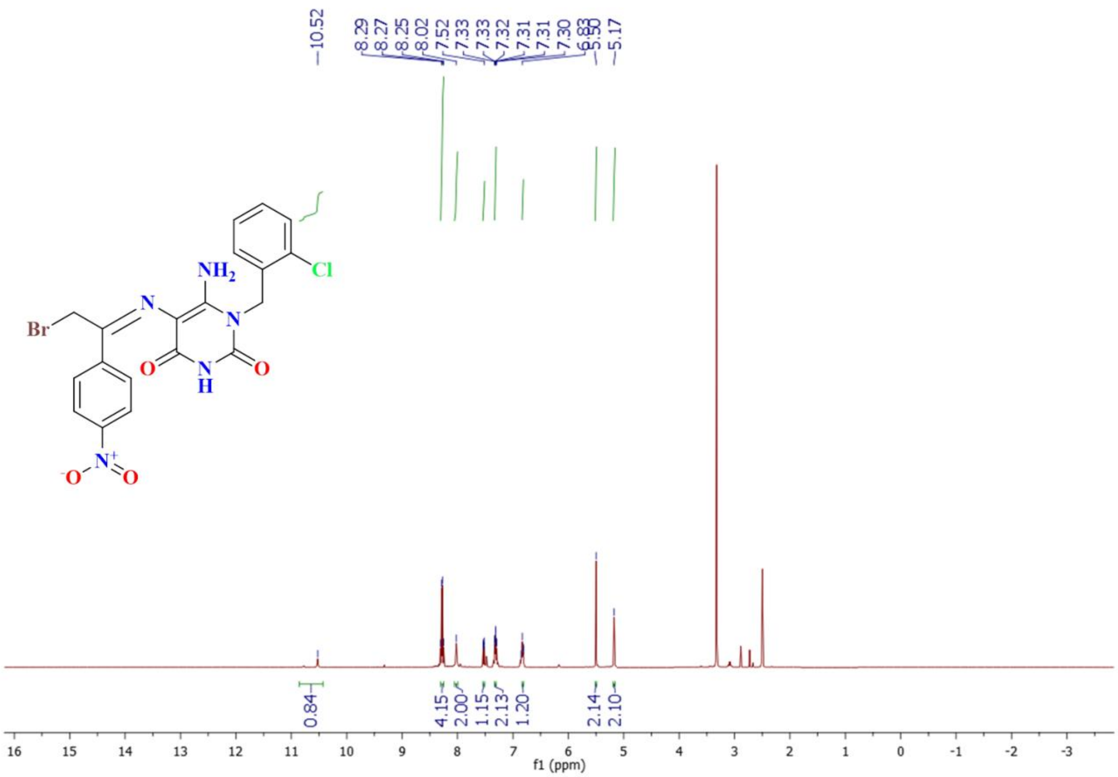

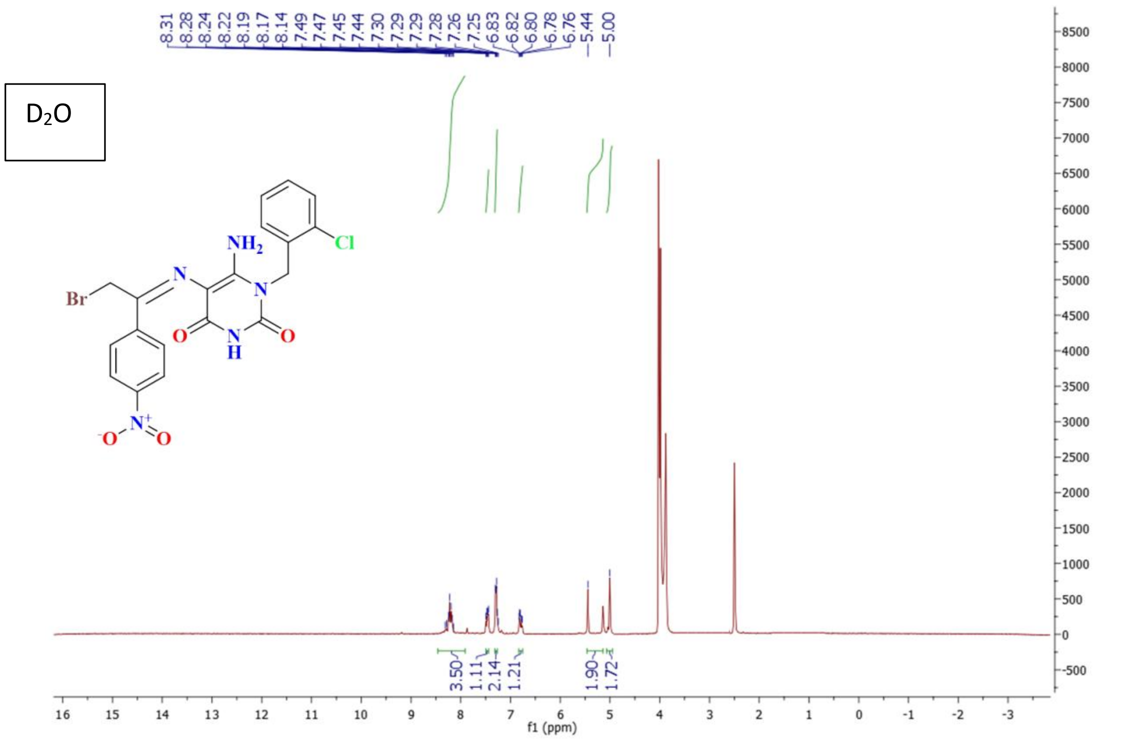

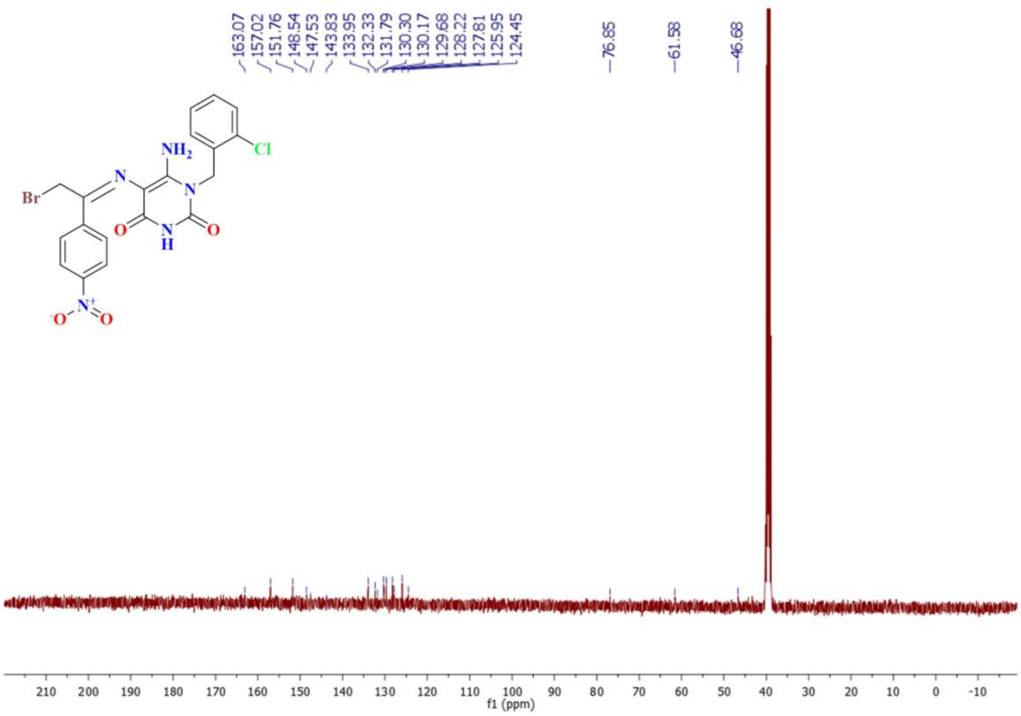
163.07
157.02
151.76
148.54
147.53
143.83
133.95
132.33
131.79
130.30
130.17
129.68
128.22
127.81
125.95
124.45
76.85
61.58
46.68
NH2
Cl
N
Br
O
N
H
O
N+
-O
O
f1 (ppm)

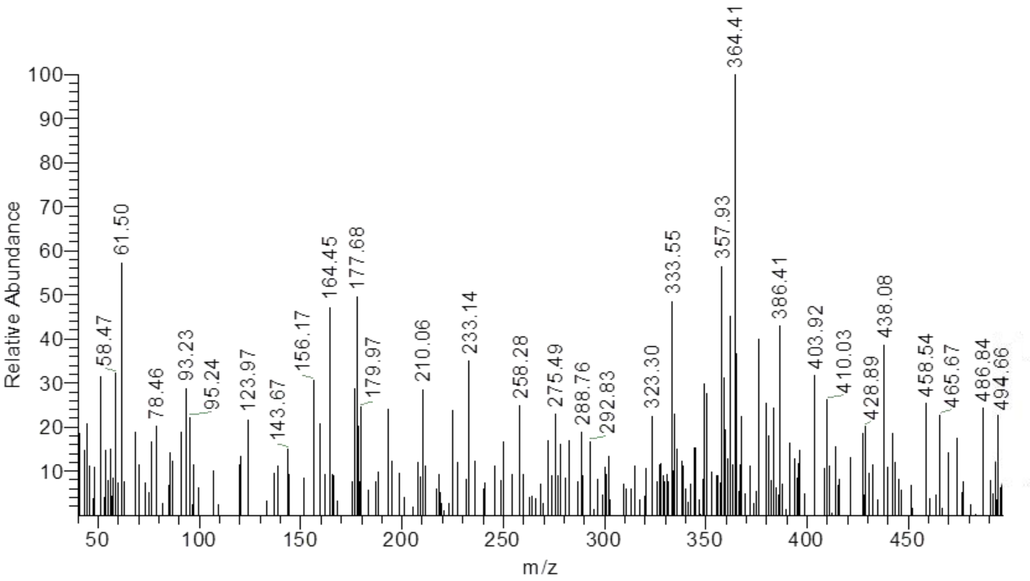
Relative Abundance
m/z
364.41
357.93
333.55
386.41
61.50
164.45
177.68
438.08
403.92
233.14
58.47
93.23
156.17
210.06
458.54
410.03
486.84
494.66
123.97
258.28
275.49
95.24
179.97
78.46
288.76
323.30
465.67
428.89
143.67
292.83

Figure S5: ^{1}H NMR, ^{13}C NMR and Mass spectra of ***(E)*-6-Amino-5-((2-bromo-1-(4-methoxyphenyl)ethylidene)amino)-1-ethylpyrimidine-2,4(1*H*,3*H*)-dione (6a)**

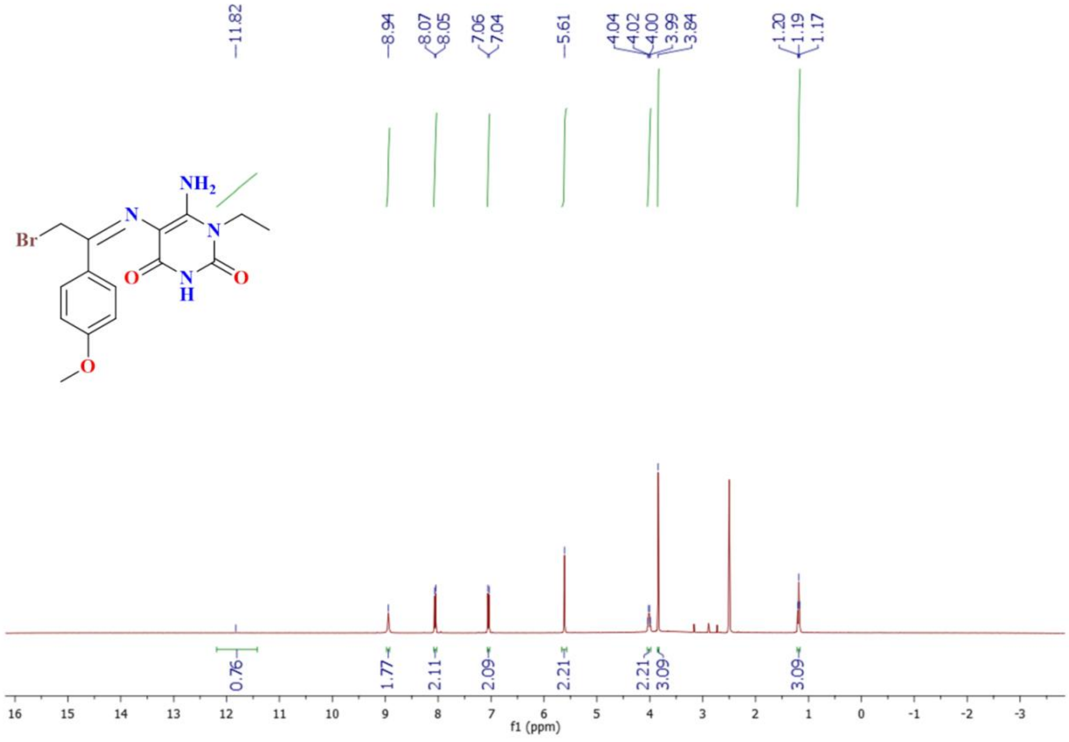

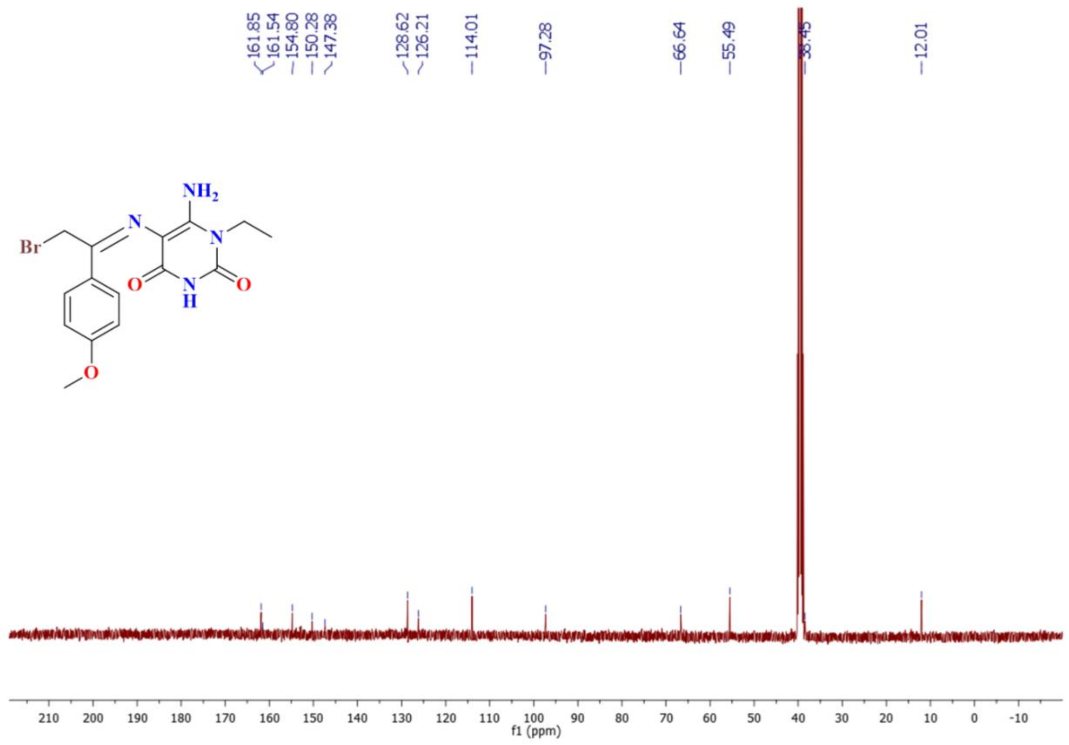

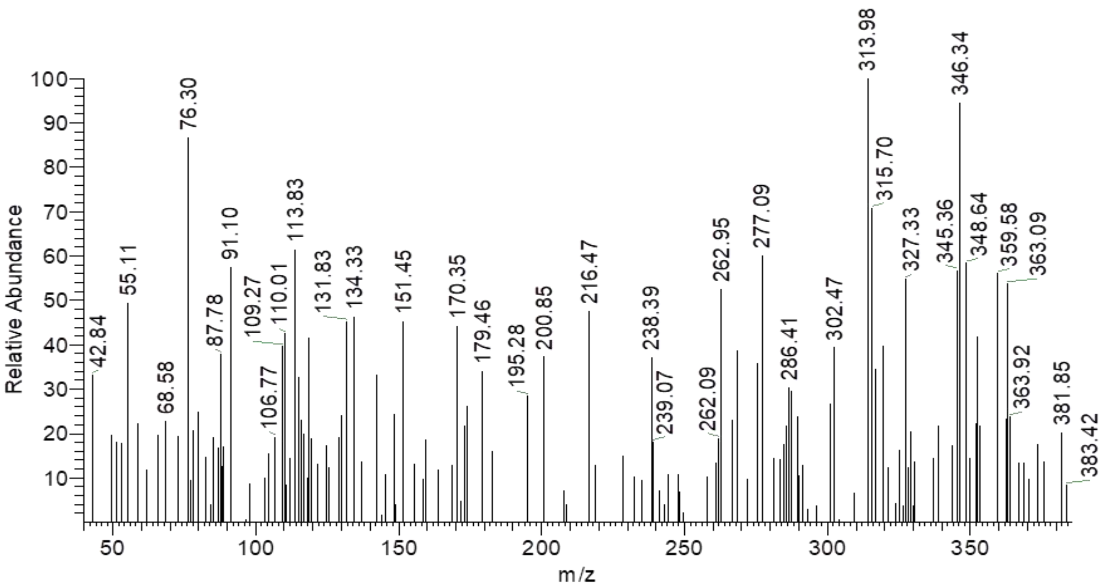

Relative Abundance
m/z
313.98
346.34
76.30
315.70
113.83
277.09
262.95
91.10
55.11
216.47
151.45
170.35
134.33
131.83
109.27
110.01
302.47
87.78
42.84
68.58
106.77
179.46
195.28
200.85
238.39
239.07
262.09
286.41
327.33
345.36
348.64
359.58
363.09
363.92
381.85
383.42

Figure S6: ^{1}H NMR, ^{13}C NMR and Mass spectra of **(*E*)-6-Amino-5-((2-bromo-1-(4-methoxyphenyl)ethylidene)amino)-1-(2-chlorobenzyl)pyrimidine-2,4(1*H*,3*H*)-dione (6b)**

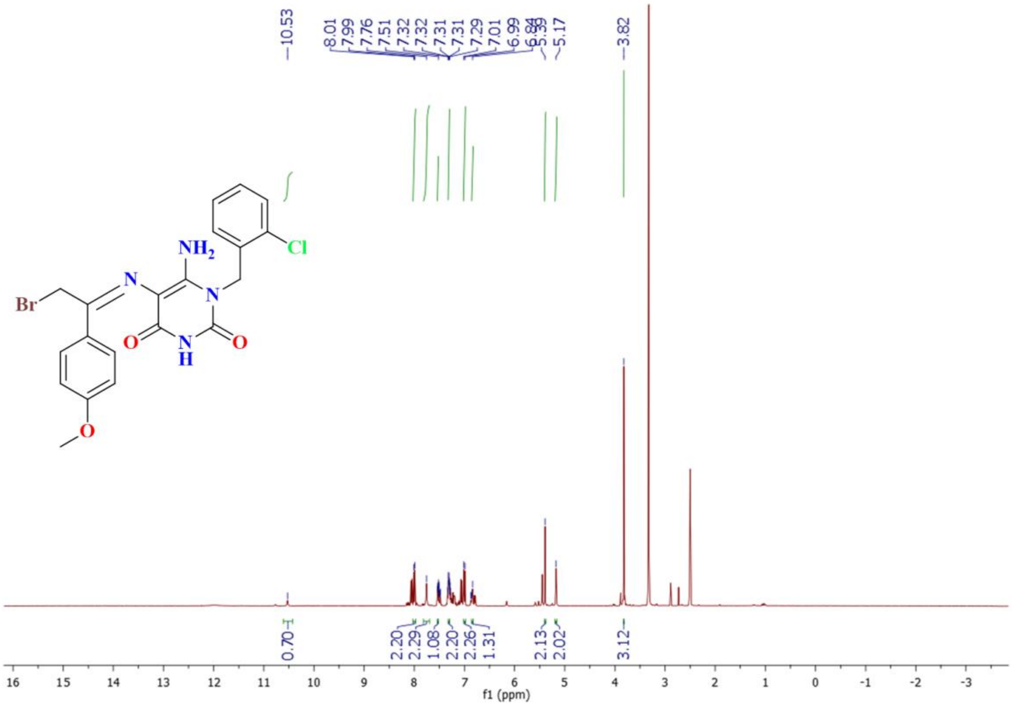

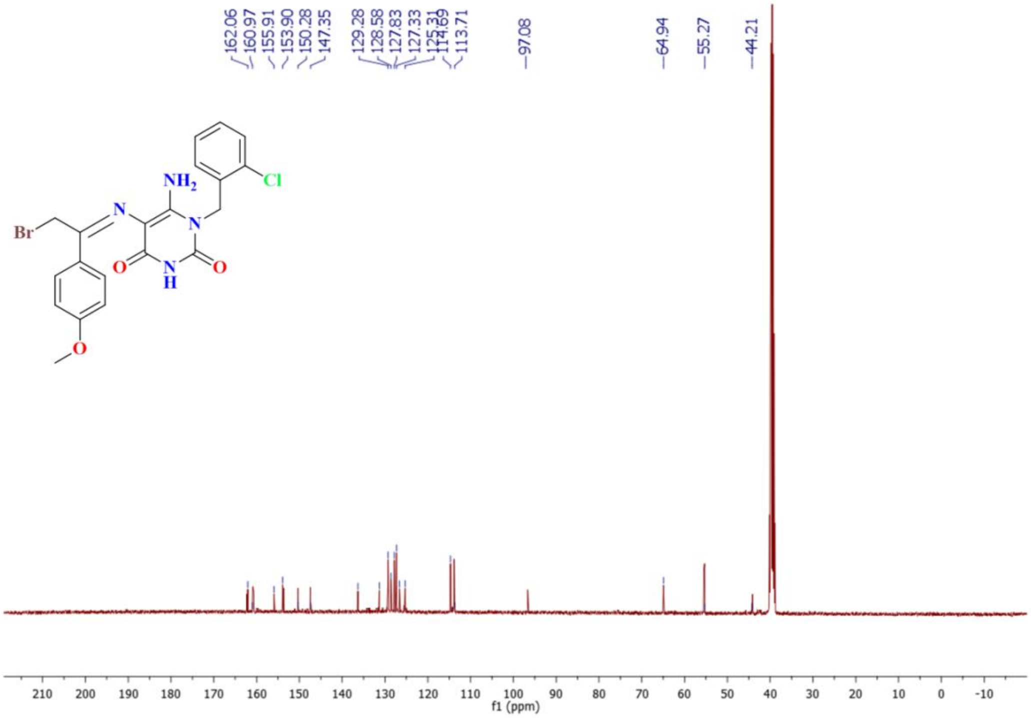

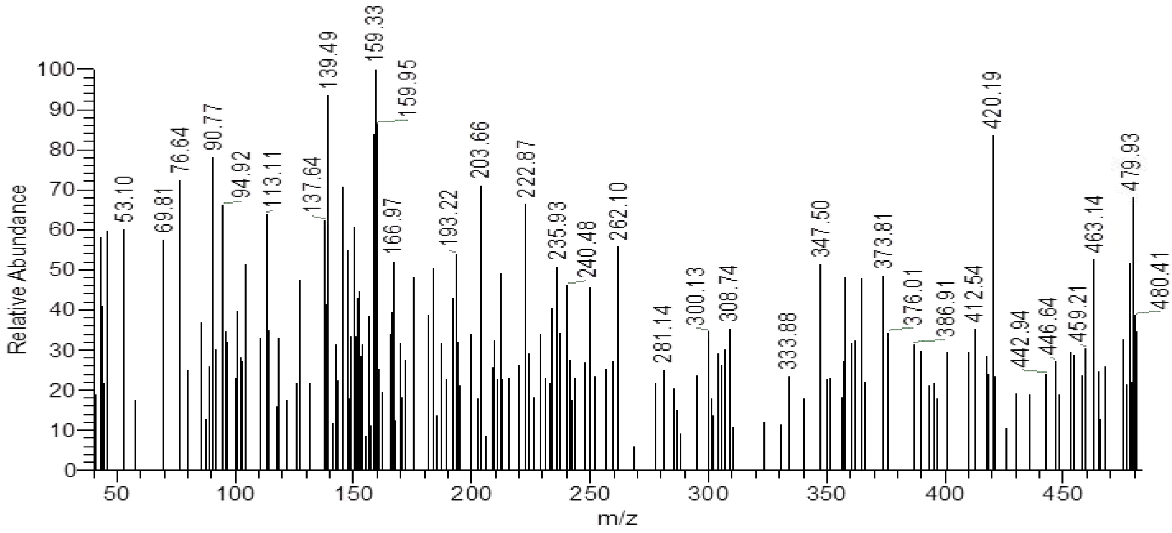

Relative Abundance
m/z
53.10
69.81
76.64
90.77
94.92
113.11
137.64
139.49
159.33
159.95
166.97
193.22
203.66
222.87
235.93
240.48
262.10
281.14
300.13
308.74
333.88
347.50
373.81
376.01
386.91
412.54
420.19
442.94
446.64
459.21
463.14
479.93
480.41

Figure S7: ^{1}H NMR, ^{13}C NMR and Mass spectra of **(*E*)-6-Amino-5-((2-bromo-1-phenylethylidene)amino)-1-methyl-2-((2-oxo-2-phenylethyl)thio)pyrimidin-4(1*H*)-one (7)**

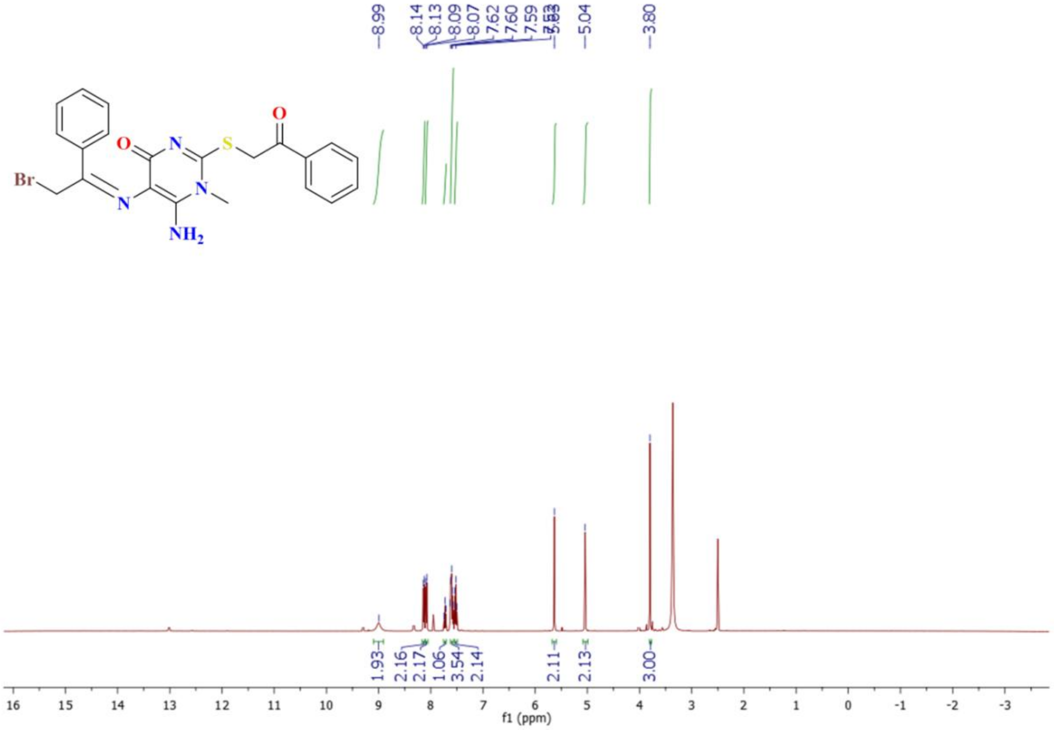

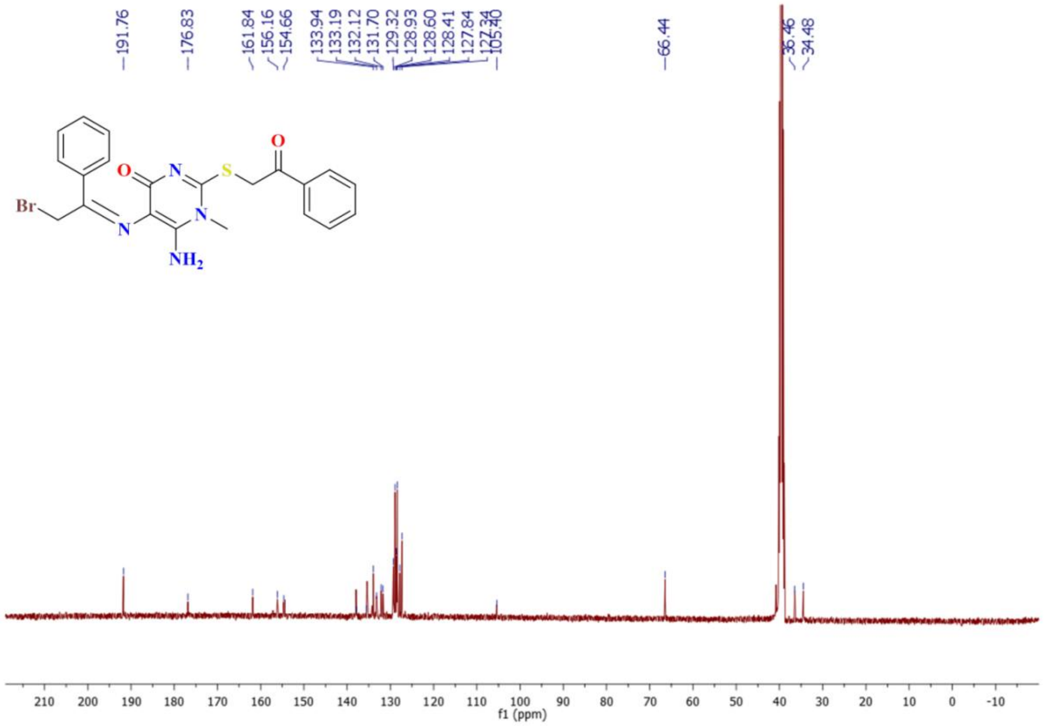

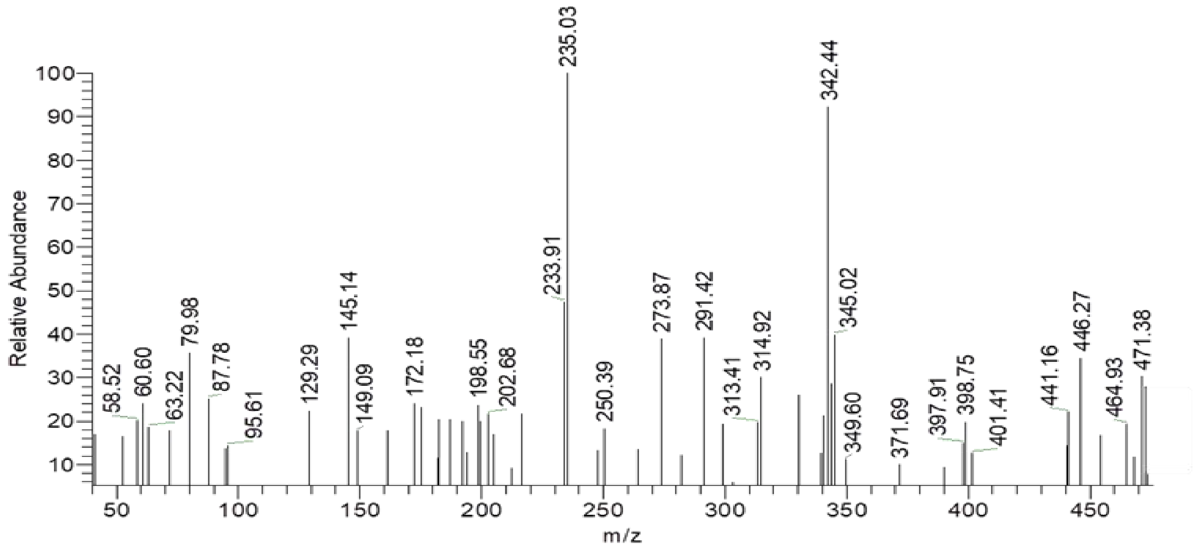

Relative Abundance
m/z
235.03
342.44
233.91
273.87
291.42
345.02
145.14
314.92
446.27
471.38
79.98
87.78
129.29
172.18
198.55
202.68
250.39
313.41
58.52
60.60
63.22
95.61
149.09
349.60
371.69
397.91
398.75
401.41
441.16
464.93

Figure S8: ^{1}H NMR, ^{13}C NMR and Mass spectra of **(*E*)-6-Amino-5-((2-bromo-1-(4-nitrophenyl)ethylidene)amino)-1-methyl-2-((2-(4-nitrophenyl)-2-oxoethyl)thio)pyrimidin-4(1*H*)-one (8)**

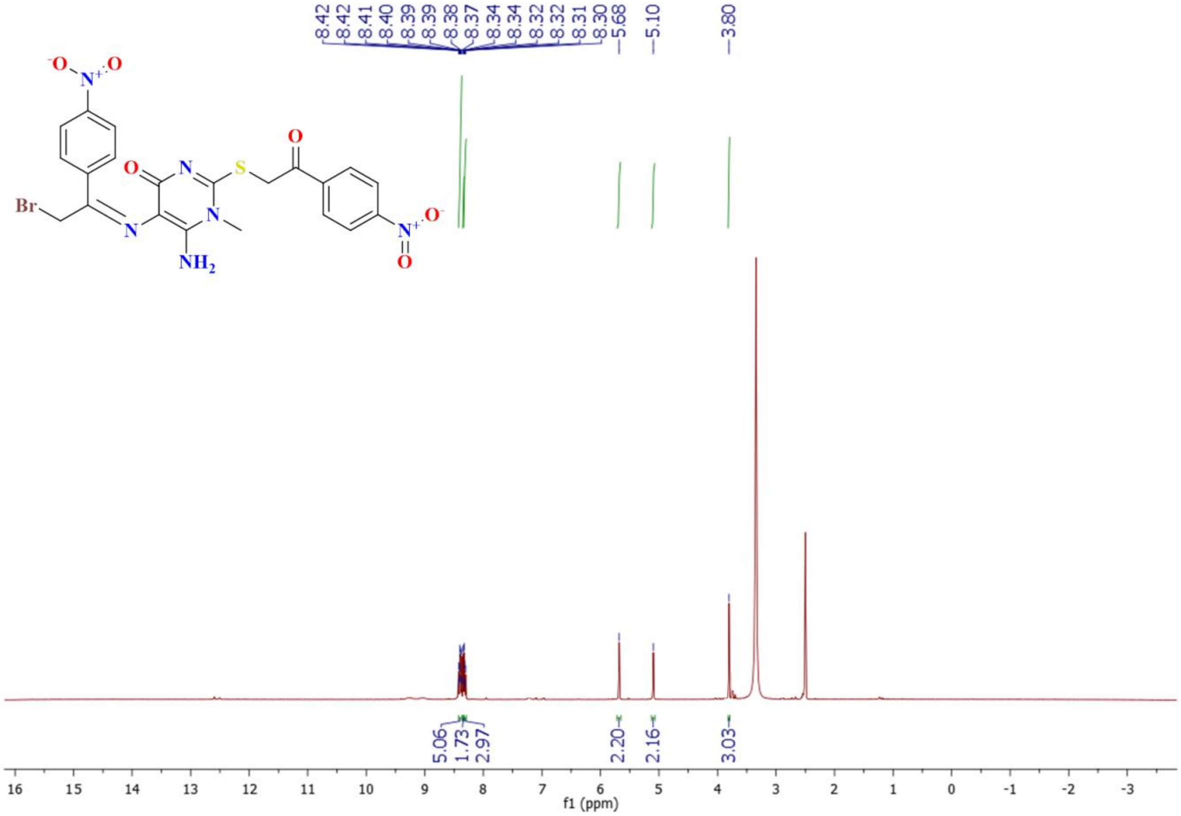

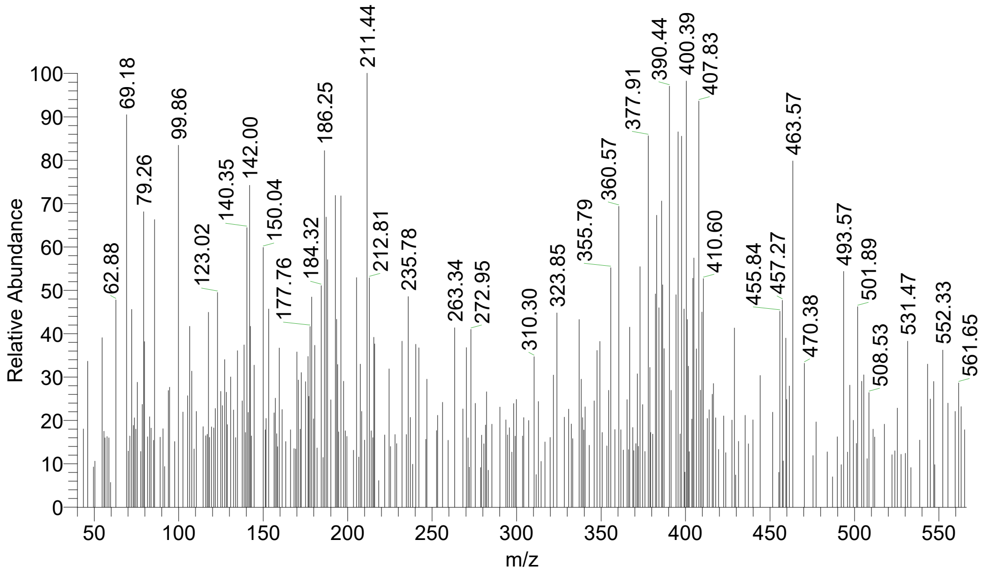

Figure S9: ^{1}H NMR, ^{13}C NMR and Mass spectra of **1-Benzyl-6-(4-methoxyphenyl)pteridine-2,4(1*H*,3*H*)-dione (9)**

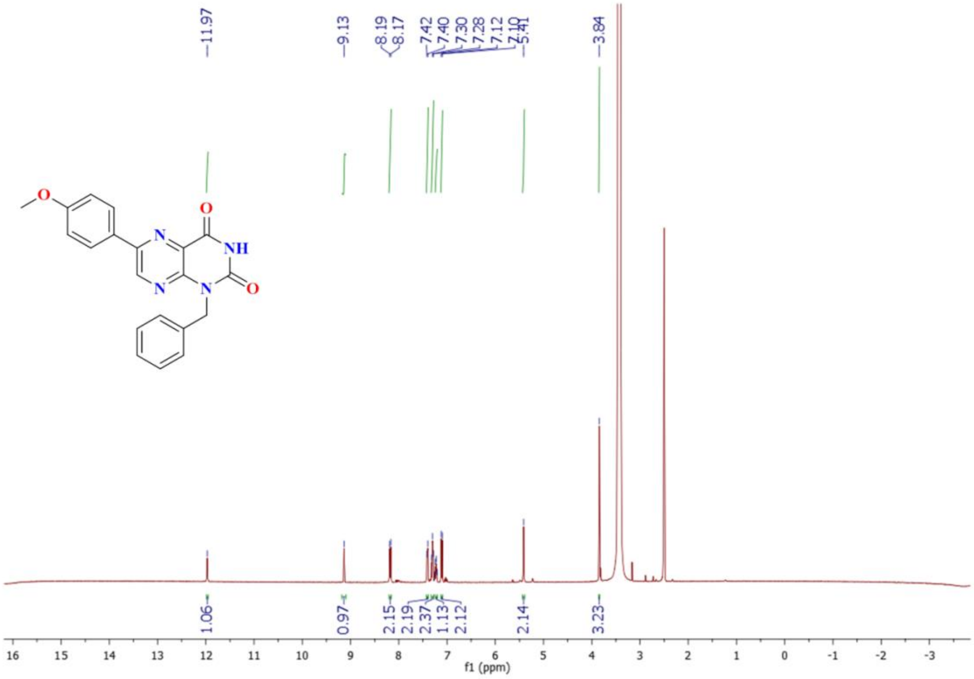

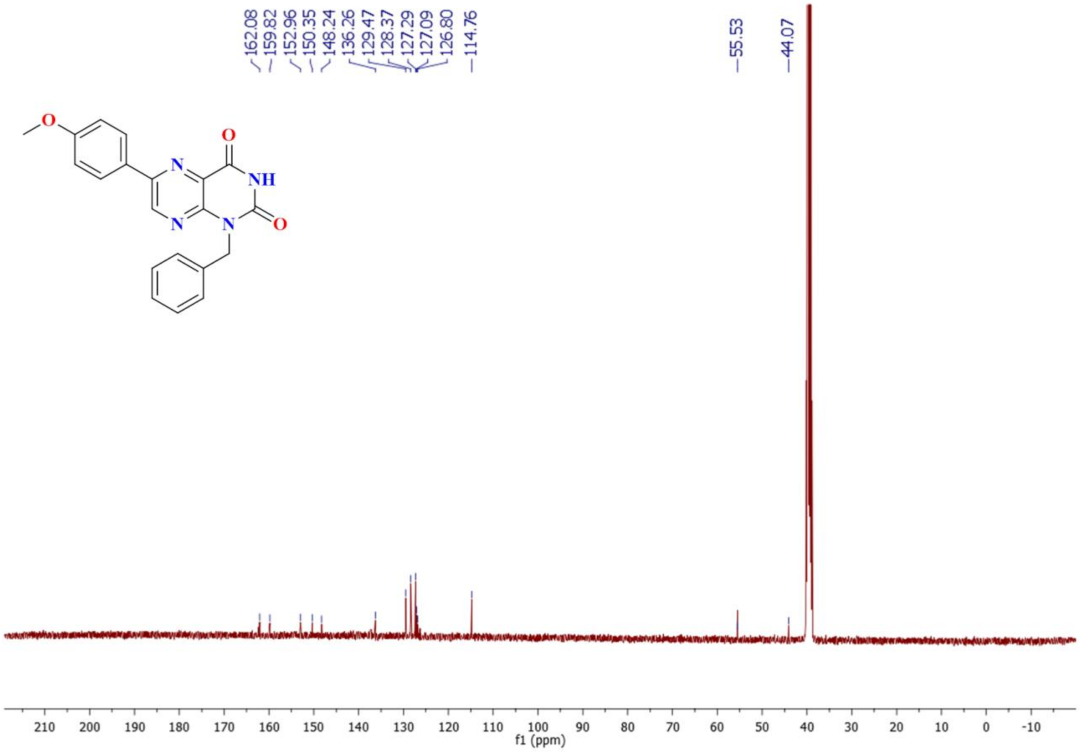

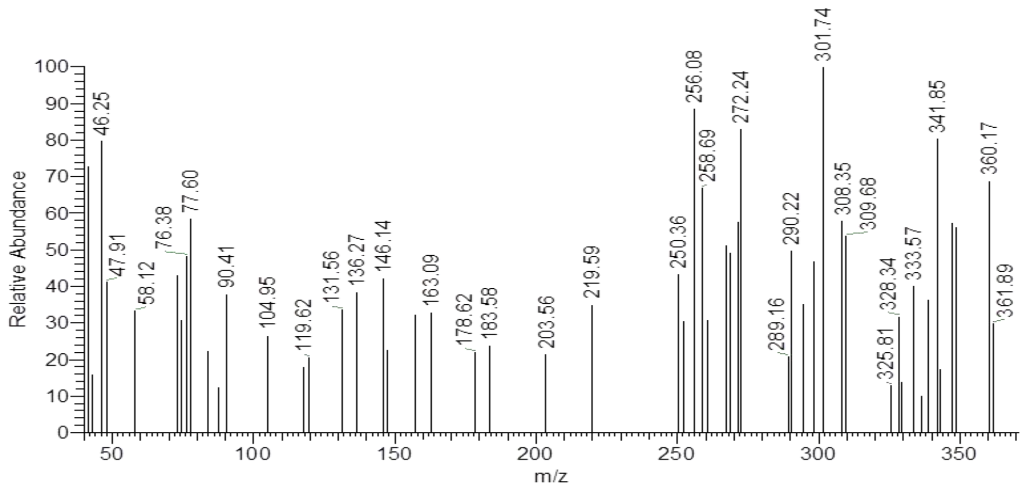

Relative Abundance
m/z
46.25
47.91
58.12
76.38
77.60
90.41
104.95
119.62
131.56
136.27
146.14
163.09
178.62
183.58
203.56
219.59
250.36
256.08
258.69
272.24
289.16
290.22
301.74
308.35
309.68
325.81
328.34
333.57
341.85
360.17
361.89

Figure S10: ^{1}H NMR, ^{13}C NMR and Mass spectra of **1-Methyl-6,7-diphenyl-2-thioxo-2,3-dihydropteridin-4(1*H*)-one (10)**

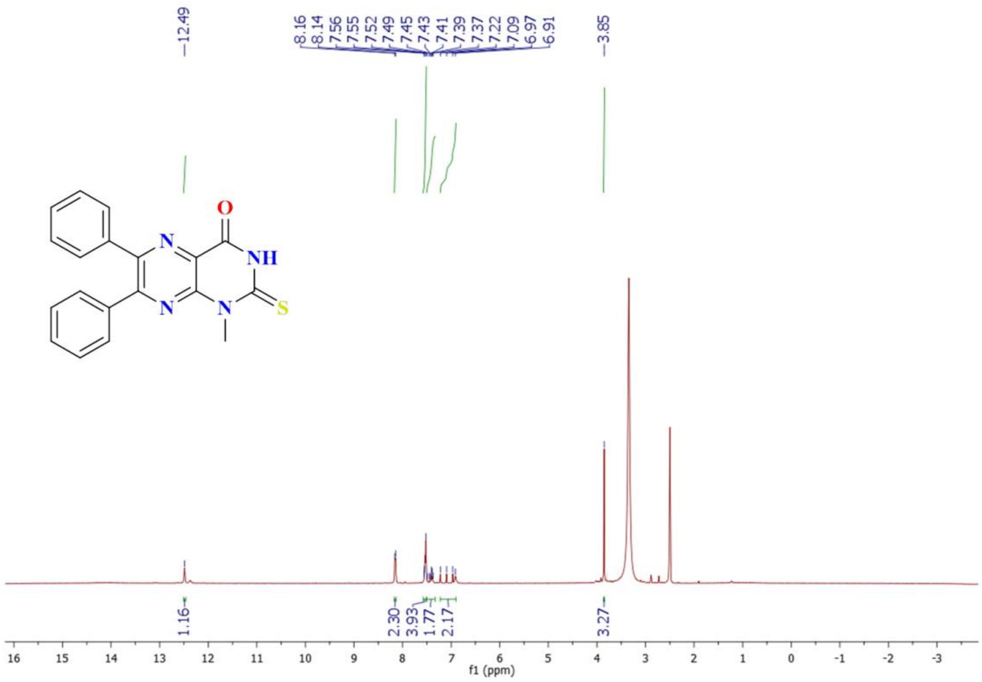

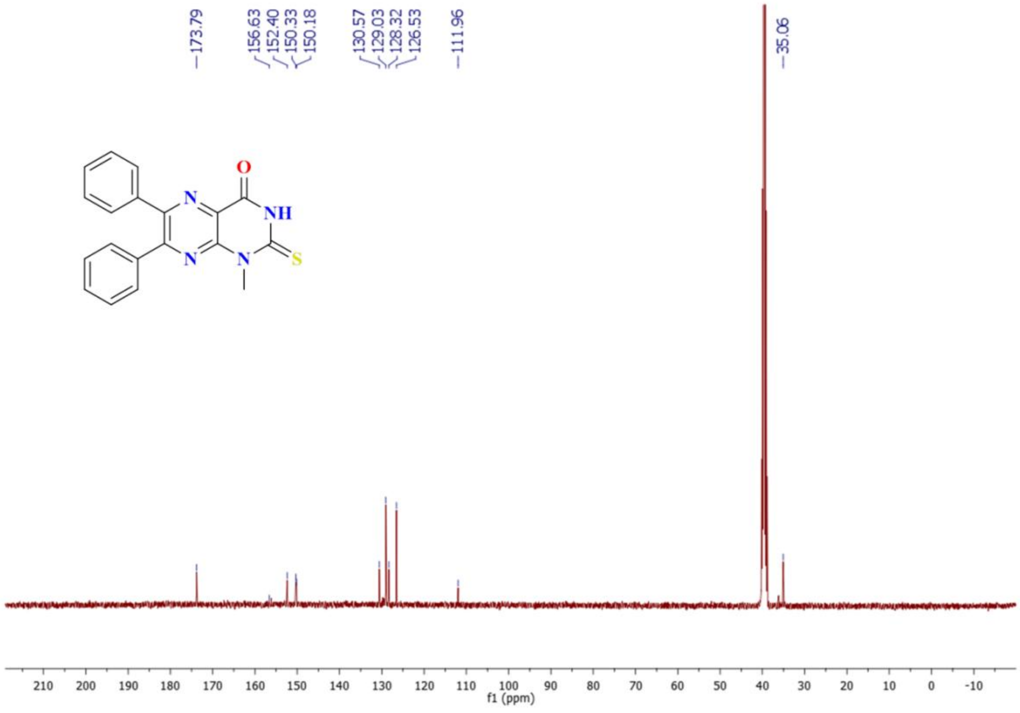

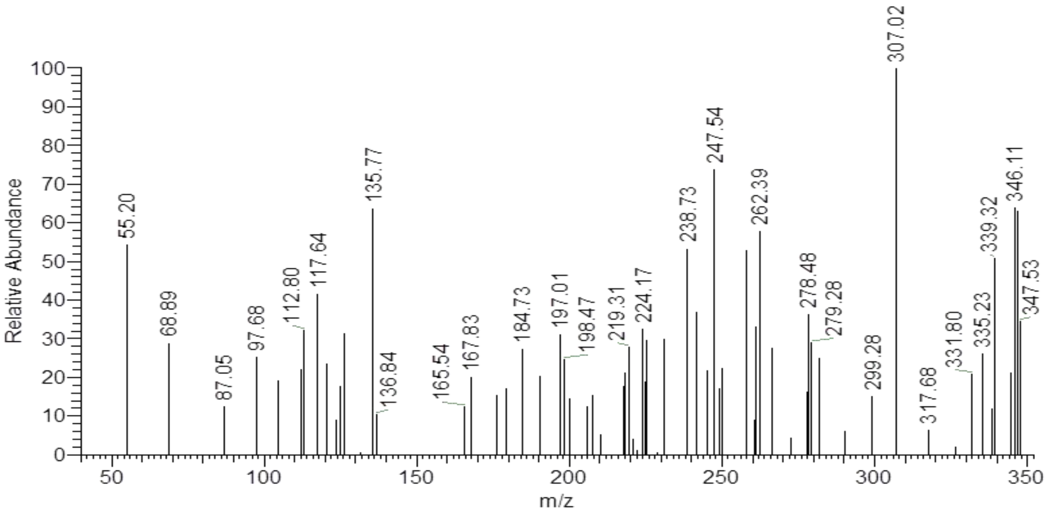

Relative Abundance
m/z
307.02
247.54
135.77
346.11
262.39
339.32
238.73
55.20
117.64
278.48
347.53
112.80
97.68
68.89
197.01
224.17
184.73
219.31
279.28
198.47
167.83
335.23
331.80
165.54
299.28
136.84
87.05
317.68

Viability assay

Effect of **4, 5a-c, 6a,b, 7, 8, 9,** and **10** on MDA-MB-231, HT-29, and U-937cells

Test code: T-2-023-16

ID	uM/ ml	O.D			Mean O.D	±SE	Viability %	Toxicity %	IC50 ± SD
MDA-MB-231	-------	0.742	0.763	0.751	0.752	0.006083	100	0	uM
1 (5b)	10	0.062	0.061	0.059	0.060667	0.000882	8.067375887	91.93262411	2.37 ± 0.06
	5	0.163	0.179	0.182	0.174667	0.005897	23.22695035	76.77304965	
	2.5	0.341	0.333	0.359	0.344333	0.007688	45.78900709	54.21099291	
	1.25	0.63	0.629	0.651	0.636667	0.007172	84.66312057	15.33687943	
	0.625	0.744	0.75	0.749	0.747667	0.001856	99.42375887	0.576241135	
	0.312	0.751	0.744	0.747	0.747333	0.002028	99.37943262	0.620567376	
2 (5c)	10	0.042	0.033	0.05	0.041667	0.00491	5.540780142	94.45921986	3.28 ± 0.04
	5	0.117	0.093	0.127	0.112333	0.010088	14.93794326	85.06205674	
	2.5	0.452	0.489	0.466	0.469	0.010786	62.36702128	37.63297872	
	1.25	0.714	0.72	0.734	0.722667	0.005925	96.09929078	3.90070922	
	0.625	0.755	0.732	0.734	0.740333	0.007356	98.44858156	1.55141844	
	0.312	0.758	0.744	0.74	0.747333	0.005457	99.37943262	0.620567376	
3 (6b)	10	0.044	0.032	0.03	0.035333	0.004372	4.69858156	95.30141844	0.63 ± 0.01
	5	0.054	0.069	0.07	0.064333	0.005175	8.554964539	91.44503546	
	2.5	0.072	0.053	0.066	0.063667	0.005608	8.466312057	91.53368794	
	1.25	0.072	0.078	0.079	0.076333	0.002186	10.15070922	89.84929078	
	0.625	0.183	0.199	0.217	0.199667	0.009821	26.55141844	73.44858156	
	0.312	0.634	0.629	0.653	0.638667	0.007311	84.92907801	15.07092199	
6 (8)	10	0.042	0.057	0.7	0.266333	0.216877	35.41666667	64.58333333	2.31 ± 0.06
	5	0.251	0.278	0.281	0.27	0.009539	35.90425532	64.09574468	
	2.5	0.341	0.317	0.328	0.328667	0.006936	43.70567376	56.29432624	
	1.25	0.644	0.629	0.618	0.630333	0.007535	83.82092199	16.17907801	
	0.625	0.719	0.708	0.722	0.716333	0.004256	95.2570922	4.742907801	
	0.312	0.752	0.718	0.732	0.734	0.009866	97.60638298	2.393617021	
7 (9)	10	0.044	0.047	0.032	0.041	0.004583	5.45212766	94.54787234	2.07 ± 0.03
	5	0.091	0.113	0.101	0.101667	0.00636	13.51950355	86.48049645	
	2.5	0.251	0.239	0.255	0.248333	0.004807	33.02304965	66.97695035	
	1.25	0.641	0.638	0.666	0.648333	0.008876	86.21453901	13.78546099	
	0.625	0.746	0.752	0.74	0.746	0.003464	99.20212766	0.79787234	
	0.312	0.753	0.736	0.761	0.75	0.007371	99.73404255	0.265957447	

ID	uM/ ml	O.D			Mean O.D	±SE	Viability %	Toxicity %	IC50 ± SD
MDA-MB-231	-------	0.742	0.763	0.751	0.752	0.006083	100	0	uM
8 (7)	10	0.033	0.042	0.036	0.037	0.002646	4.920212766	95.07978723	0.4 ± 0.01
	5	0.081	0.069	0.099	0.083	0.008718	11.03723404	88.96276596	
	2.5	0.092	0.074	0.099	0.088333	0.007446	11.7464539	88.2535461	
	1.25	0.152	0.117	0.103	0.124	0.014572	16.4893617	83.5106383	
	0.625	0.198	0.237	0.216	0.217	0.011269	28.85638298	71.14361702	
	0.312	0.418	0.439	0.43	0.429	0.006083	57.04787234	42.95212766	
9 (4)	10	0.028	0.041	0.037	0.035333	0.003844	4.69858156	95.30141844	0.78 ± 0.01
	5	0.044	0.028	0.043	0.038333	0.005175	5.09751773	94.90248227	
	2.5	0.053	0.067	0.061	0.060333	0.004055	8.023049645	91.97695035	
	1.25	0.054	0.048	0.059	0.053667	0.00318	7.136524823	92.86347518	
	0.625	0.426	0.419	0.458	0.434333	0.012005	57.7570922	42.2429078	
	0.312	0.753	0.76	0.736	0.749667	0.007126	99.68971631	0.310283688	
12 (10)	10	0.07	0.053	0.056	0.059667	0.005239	7.934397163	92.06560284	3.4 ± 0.02
	5	0.213	0.184	0.197	0.198	0.008386	26.32978723	73.67021277	
	2.5	0.398	0.427	0.401	0.408667	0.009207	54.34397163	45.65602837	
	1.25	0.731	0.748	0.75	0.743	0.006028	98.80319149	1.196808511	
	0.625	0.762	0.741	0.753	0.752	0.006083	100	0	
	0.312	0.752	0.755	0.749	0.752	0.001732	100	0	
13 (5a)	10	0.042	0.028	0.025	0.031667	0.005239	4.210992908	95.78900709	3.24 ± 0.03
	5	0.166	0.193	0.178	0.179	0.00781	23.80319149	76.19680851	
	2.5	0.415	0.382	0.399	0.398667	0.009528	53.0141844	46.9858156	
	1.25	0.682	0.701	0.674	0.685667	0.008007	91.17907801	8.820921986	
	0.625	0.755	0.742	0.749	0.748667	0.003756	99.55673759	0.443262411	
	0.312	0.76	0.744	0.746	0.75	0.005033	99.73404255	0.265957447	
14 (6a)	10	0.03	0.028	0.033	0.030333	0.001453	4.033687943	95.96631206	1 ± 0.02
	5	0.027	0.024	0.025	0.025333	0.000882	3.368794326	96.63120567	
	2.5	0.051	0.038	0.046	0.045	0.003786	5.984042553	94.01595745	
	1.25	0.215	0.185	0.211	0.203667	0.009404	27.08333333	72.91666667	
	0.625	0.745	0.732	0.755	0.744	0.006658	98.93617021	1.063829787	
	0.312	0.758	0.746	0.751	0.751667	0.00348	99.95567376	0.044326241	

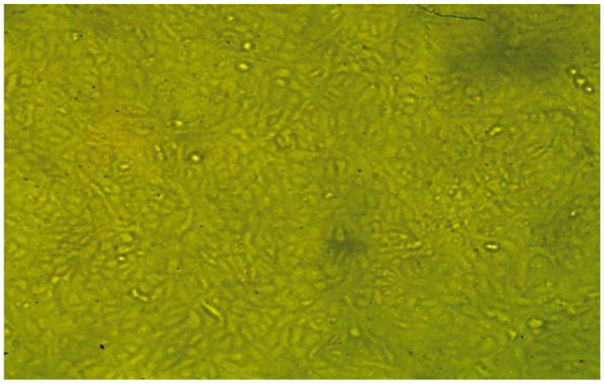

control
MDA-MB 231

Organism : *Homo sapiens*, human
Tissue : Breast; Mammary gland
Cell Type : epithelial
Culture Properties : adherent
Disease : adenocarcinoma
ATCC : HTB-26

Effect of sample 1 on MDA cells at different concentration

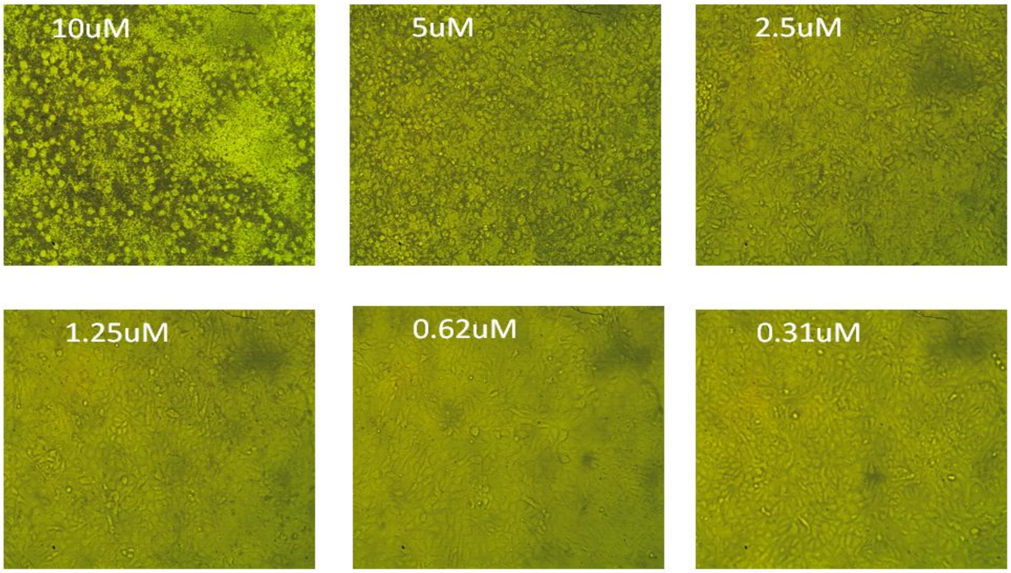

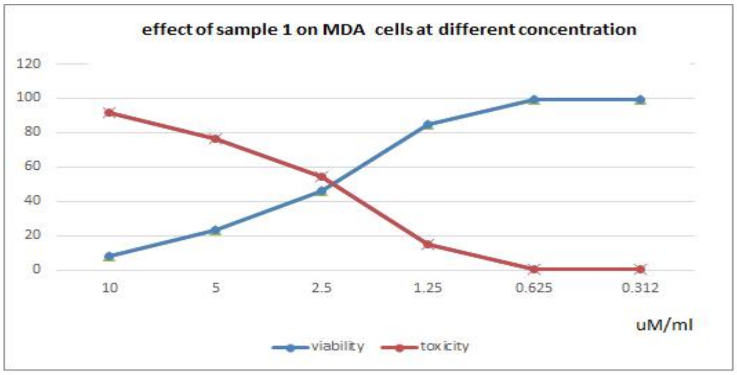

Effect of sample 2 on MDA cells at different concentration

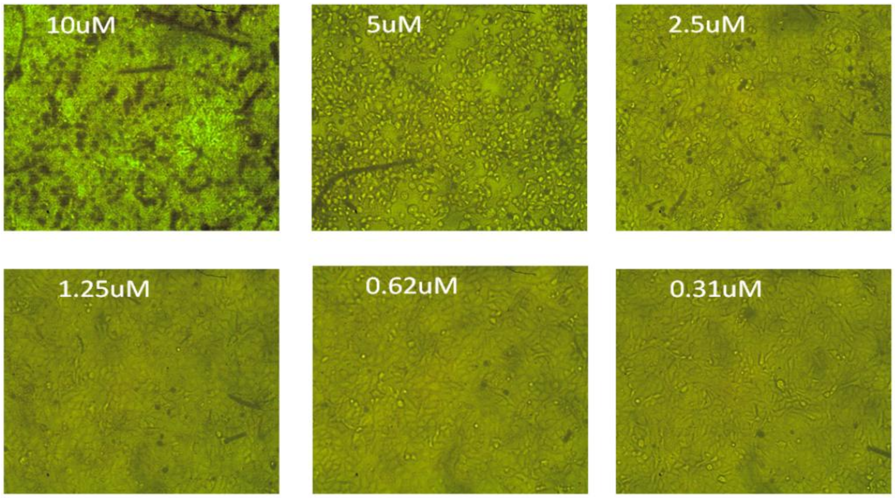

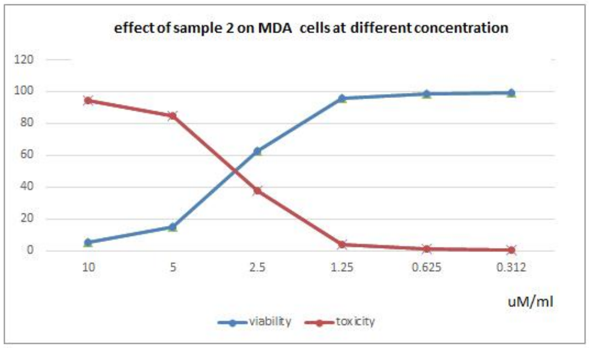

Effect of sample 3 on MDA cells at different concentration

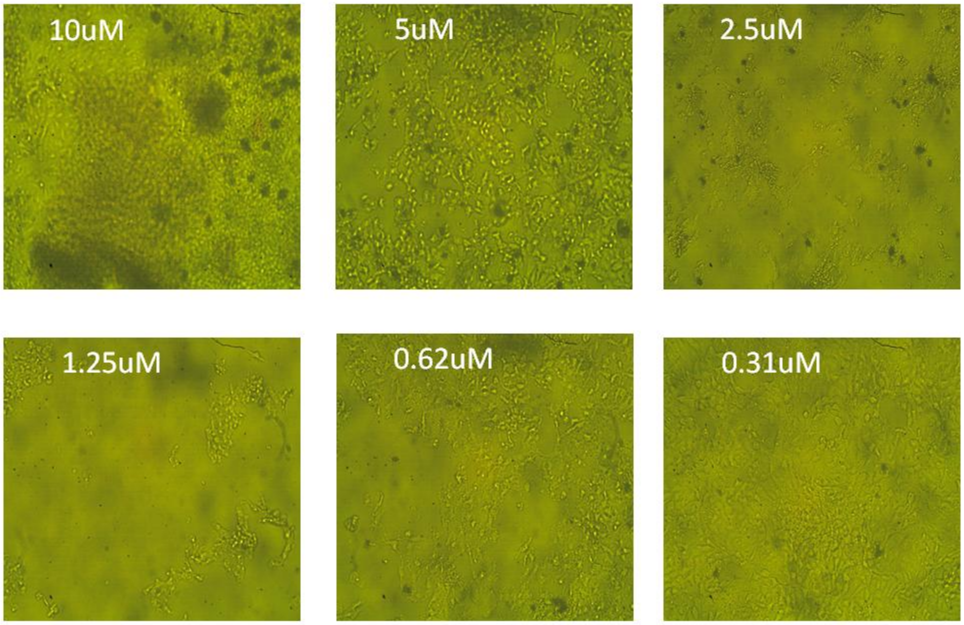

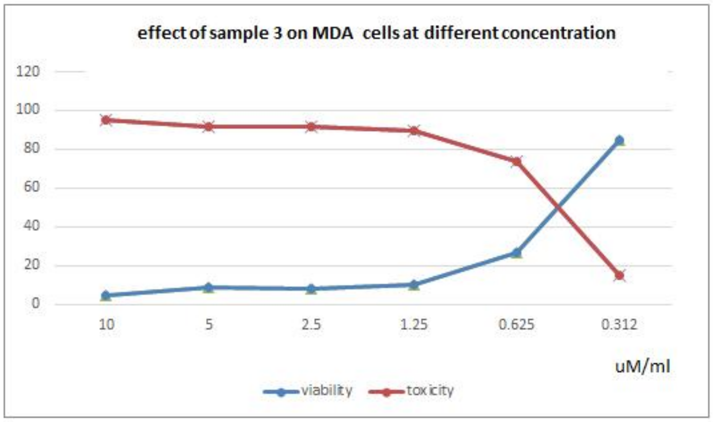

Effect of sample 6 on MDA cells at different concentration

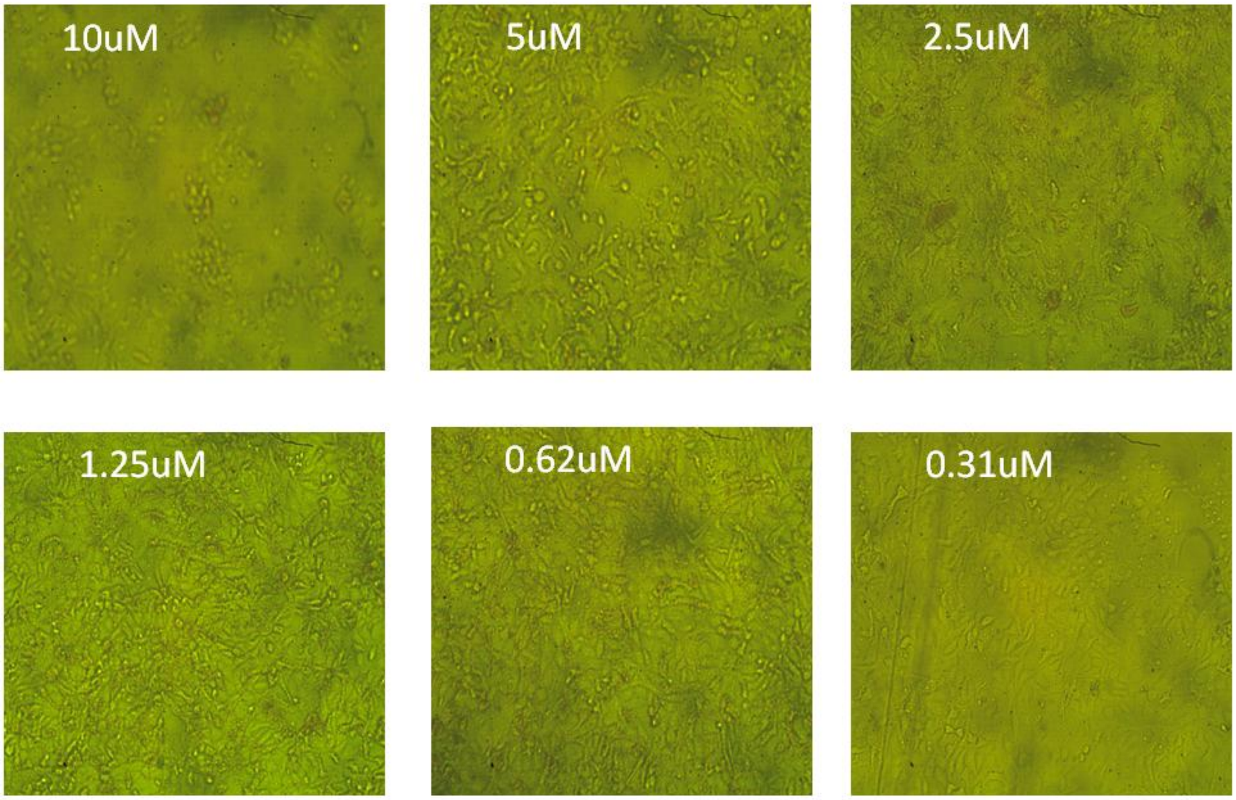

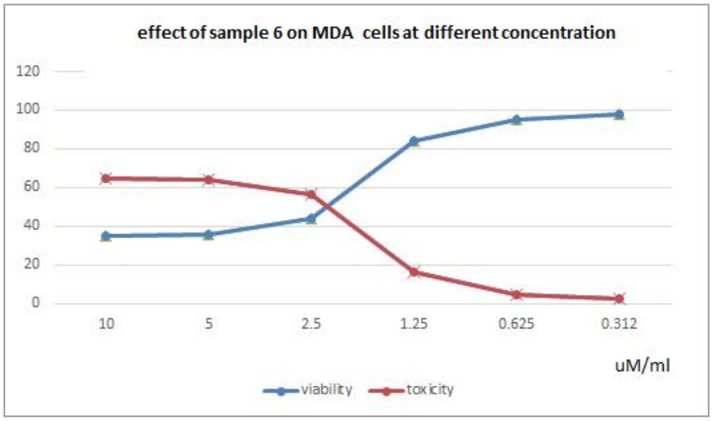

Effect of sample 7 on MDA cells at different concentration

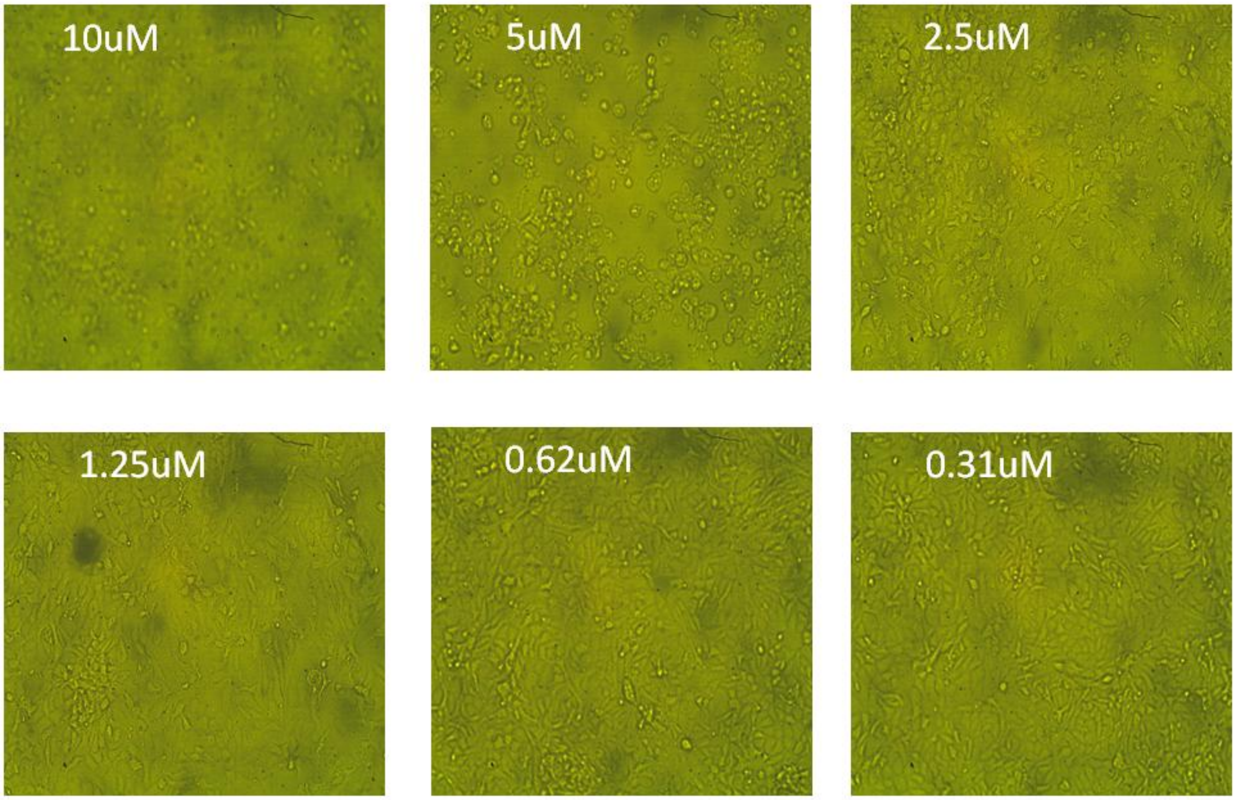

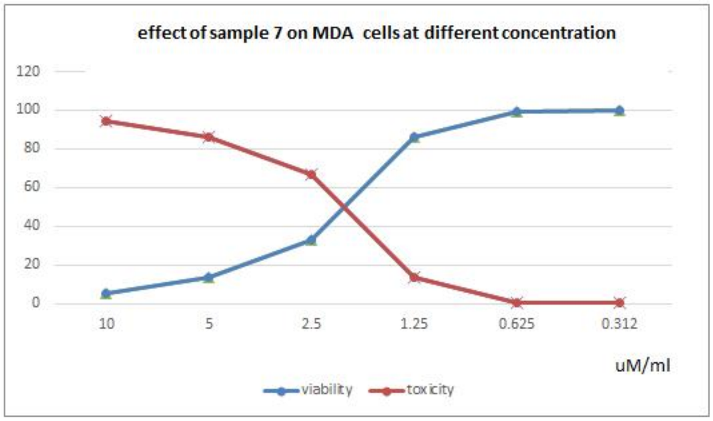

Effect of sample 8 on MDA cells at different concentration

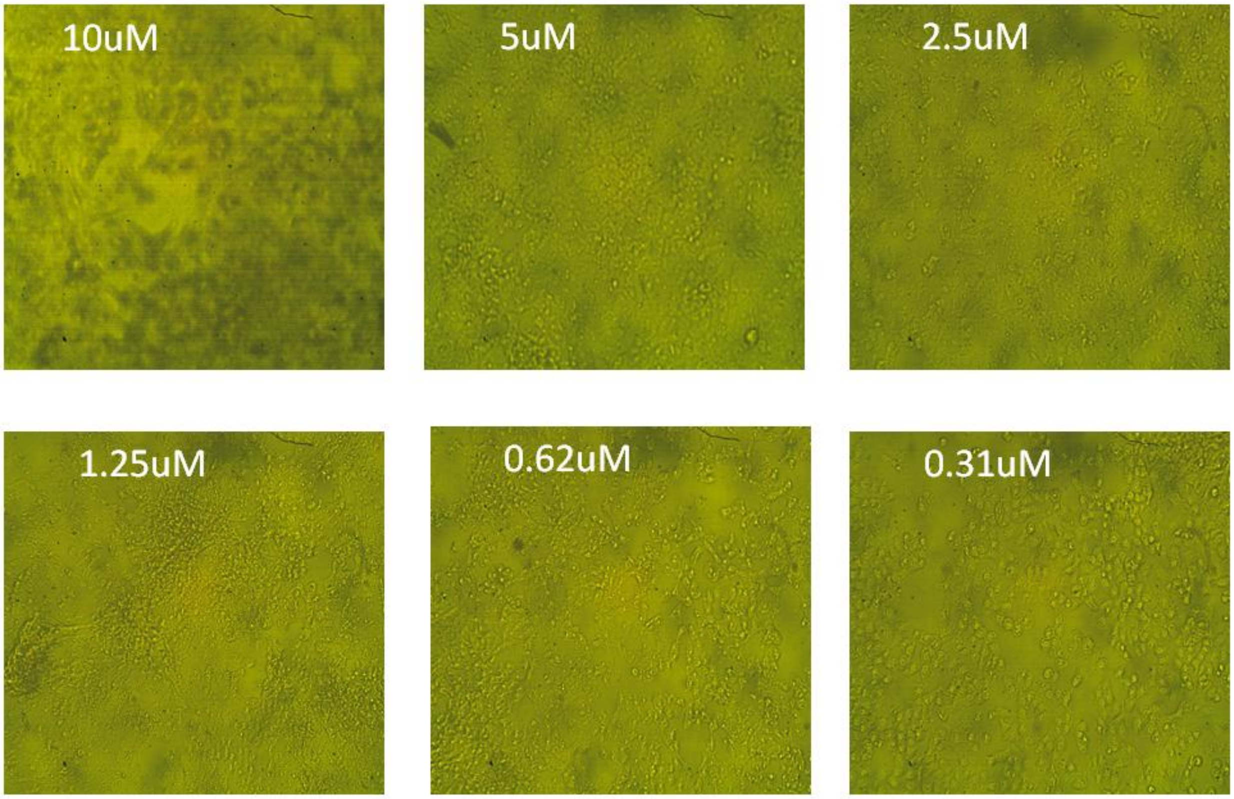

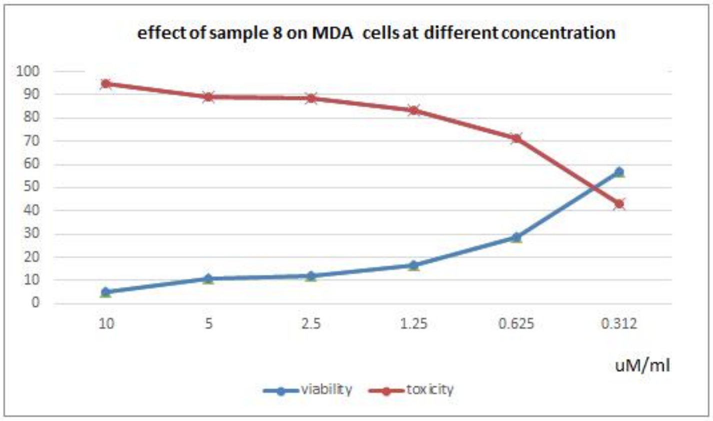

Effect of sample 9 on MDA cells at different concentration

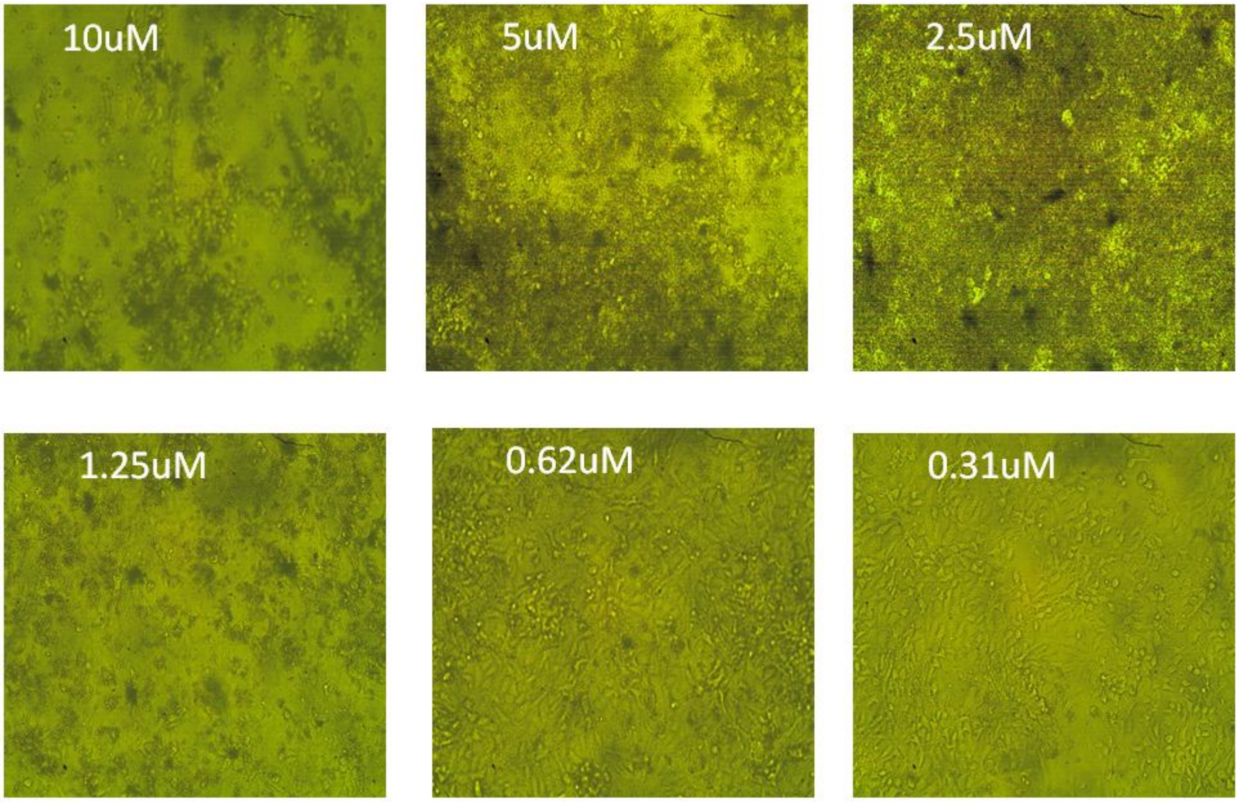

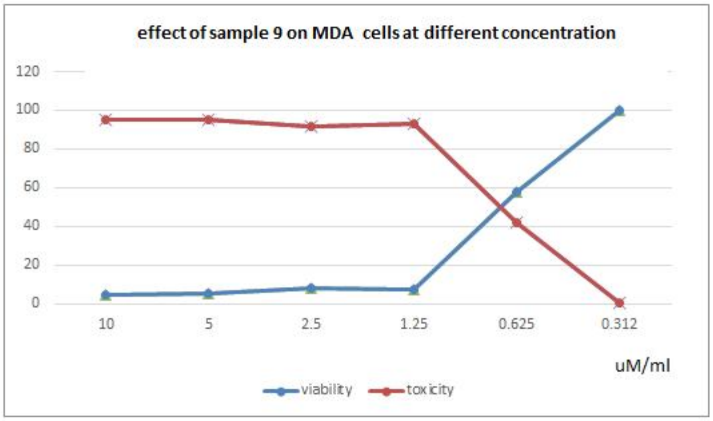

Effect of sample 12 on MDA cells at different concentration

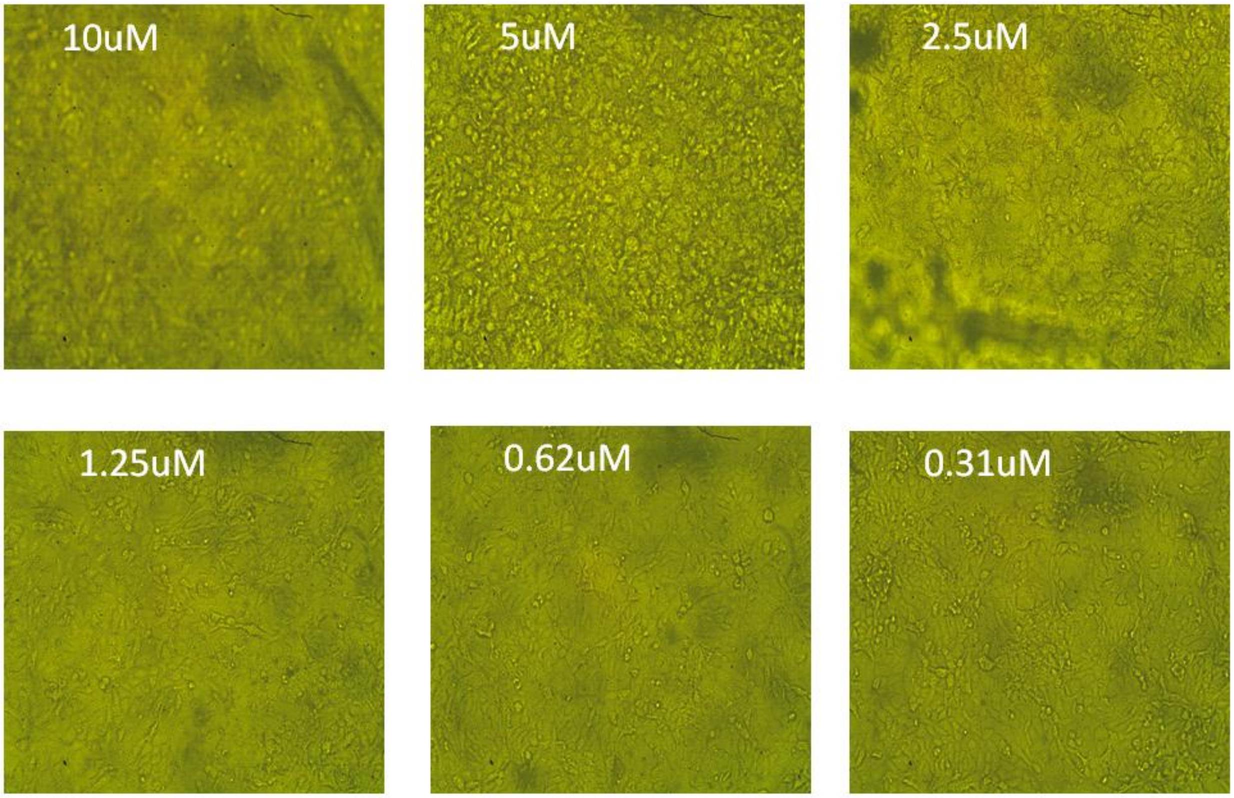

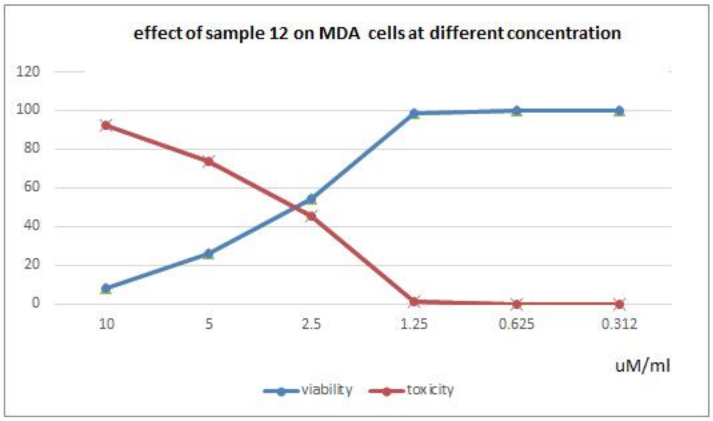

Effect of sample 13 on MDA cells at different concentration

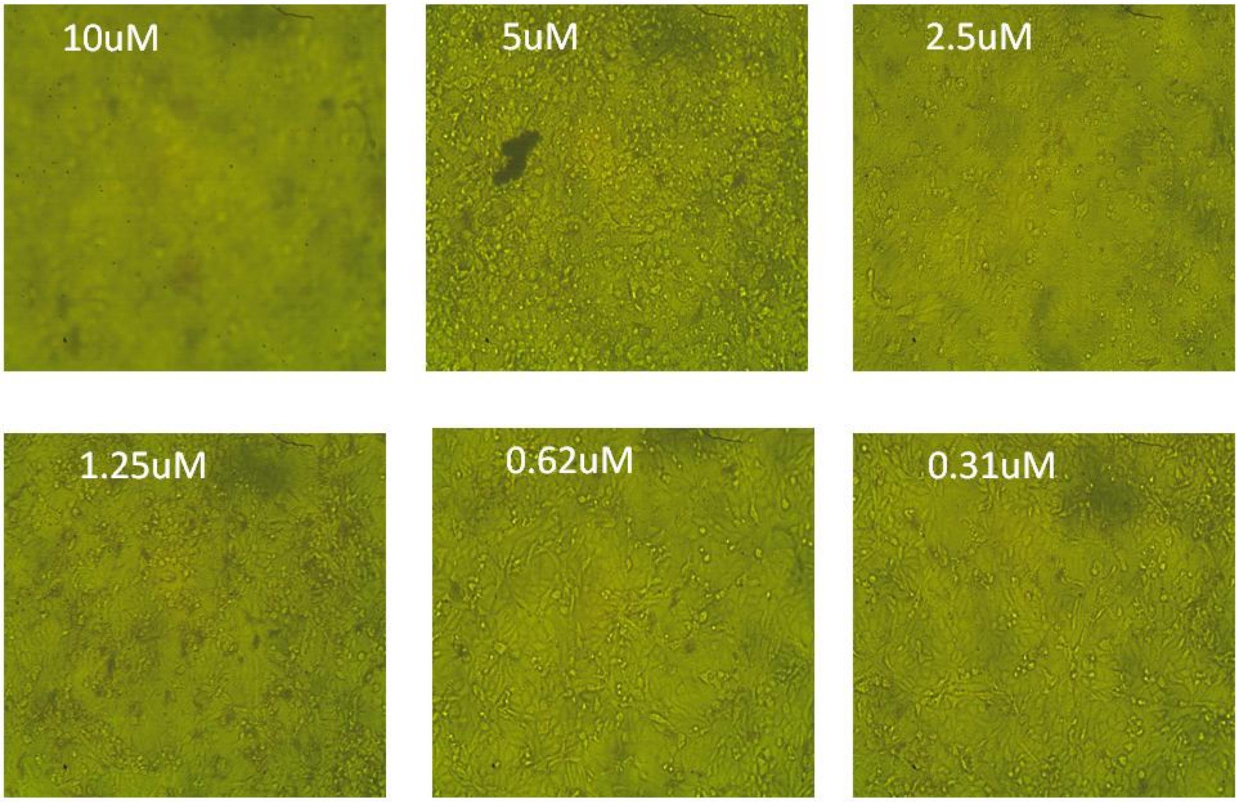

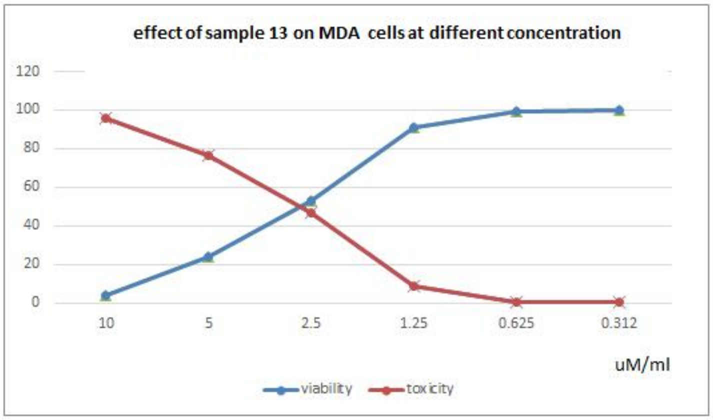

Effect of sample 14 on MDA cells at different concentration

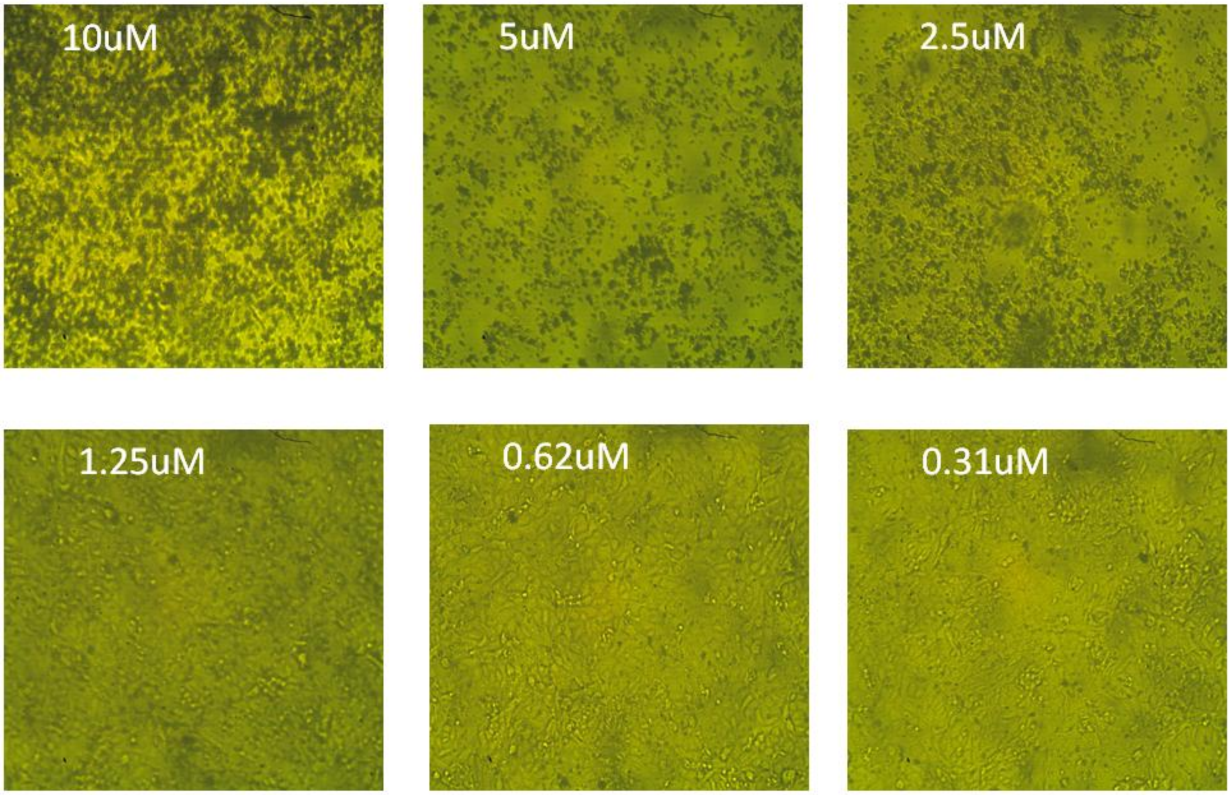

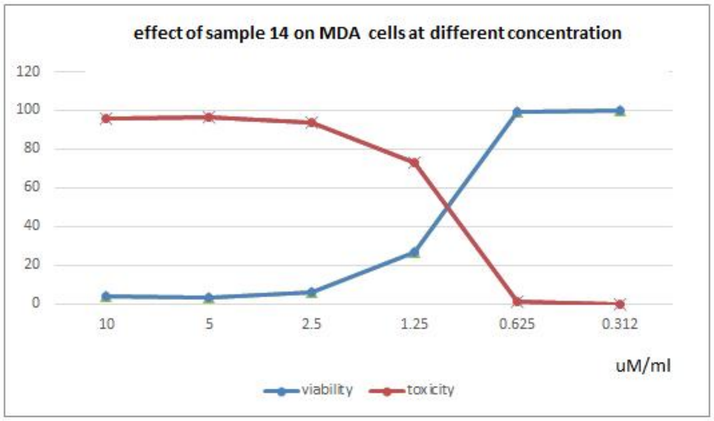

ID	uM/ ml	O.D			Mean O.D	±SE	Viability %	Toxicity %	IC50 ± SD
**HT29**	-------	0.763	0.777	0.782	0.774	0.005686	100	0	uM
1 (5b)	10	0.094	0.091	0.088	0.091	0.001732	11.75710594	88.24289406	3.66 ± 0.05
	5	0.173	0.199	0.216	0.196	0.012503	25.32299742	74.67700258	
	2.5	0.573	0.562	0.551	0.562	0.006351	72.60981912	27.39018088	
	1.25	0.743	0.769	0.65	0.720667	0.036122	93.10938846	6.890611542	
	0.625	0.781	0.772	0.766	0.773	0.004359	99.87080103	0.129198966	
	0.312	0.768	0.774	0.78	0.774	0.003464	100	0	
2 (5c)	10	0.042	0.038	0.051	0.043667	0.003844	5.6416882	94.3583118	2.24 ± 0.04
	5	0.044	0.057	0.042	0.047667	0.004702	6.158484065	93.84151593	
	2.5	0.286	0.317	0.326	0.309667	0.012115	40.00861326	59.99138674	
	1.25	0.713	0.739	0.721	0.724333	0.007688	93.583118	6.416881998	
	0.625	0.768	0.77	0.779	0.772333	0.003383	99.78466839	0.215331611	
	0.312	0.772	0.779	0.768	0.773	0.003215	99.87080103	0.129198966	
3 (6b)	10	0.048	0.055	0.043	0.048667	0.00348	6.287683032	93.71231697	0.75 ± 0.01
	5	0.042	0.033	0.027	0.034	0.004359	4.392764858	95.60723514	
	2.5	0.027	0.031	0.036	0.031333	0.002603	4.048234281	95.95176572	
	1.25	0.03	0.035	0.032	0.032333	0.001453	4.177433247	95.82256675	
	0.625	0.423	0.399	0.401	0.407667	0.007688	52.67011197	47.32988803	
	0.312	0.763	0.772	0.778	0.771	0.004359	99.6124031	0.387596899	
6 (8)	10	0.163	0.168	0.182	0.171	0.005686	22.09302326	77.90697674	6.48 ± 0.08
	5	0.401	0.372	0.38	0.384333	0.008647	49.65546942	50.34453058	
	2.5	0.77	0.765	0.778	0.771	0.003786	99.6124031	0.387596899	
	1.25	0.774	0.771	0.764	0.769667	0.002963	99.44013781	0.559862188	
	0.625	0.768	0.779	0.772	0.773	0.003215	99.87080103	0.129198966	
	0.312	0.771	0.767	0.782	0.773333	0.004485	99.91386736	0.086132644	
7 (9)	10	0.073	0.091	0.099	0.087667	0.007688	11.32644272	88.67355728	4.11 ± 0.05
	5	0.241	0.238	0.259	0.246	0.006557	31.78294574	68.21705426	
	2.5	0.652	0.673	0.71	0.678333	0.016954	87.63996555	12.36003445	
	1.25	0.78	0.771	0.769	0.773333	0.003383	99.91386736	0.086132644	
	0.625	0.776	0.772	0.772	0.773333	0.001333	99.91386736	0.086132644	
	0.312	0.767	0.773	0.776	0.772	0.002646	99.74160207	0.258397933	

ID	uM/ ml	O.D			Mean O.D	±SE	Viability %	Toxicity %	IC50 ± SD
HT29	-------	0.763	0.777	0.782	0.774	0.005686	100	0	uM
8 (7)	10	0.036	0.052	0.058	0.048667	0.006566	6.287683032	93.71231697	0.79 ± 0.01
	5	0.048	0.055	0.053	0.052	0.002082	6.718346253	93.28165375	
	2.5	0.1	0.074	0.089	0.087667	0.007535	11.32644272	88.67355728	
	1.25	0.118	0.094	0.104	0.105333	0.00696	13.6089578	86.3910422	
	0.625	0.425	0.401	0.438	0.421333	0.010837	54.43583118	45.56416882	
	0.312	0.762	0.777	0.782	0.773667	0.006009	99.95693368	0.043066322	
9 (4)	10	0.047	0.052	0.044	0.047667	0.002333	6.158484065	93.84151593	0.76 ± 0
	5	0.057	0.063	0.048	0.056	0.004359	7.235142119	92.76485788	
	2.5	0.052	0.066	0.057	0.058333	0.004096	7.536606374	92.46339363	
	1.25	0.067	0.063	0.053	0.061	0.004163	7.881136951	92.11886305	
	0.625	0.399	0.382	0.425	0.402	0.012503	51.9379845	48.0620155	
	0.312	0.765	0.784	0.77	0.773	0.005686	99.87080103	0.129198966	
12 (10)	10	0.027	0.025	0.034	0.028667	0.002728	3.703703704	96.2962963	5.63 ± 0.13
	5	0.463	0.428	0.477	0.456	0.014572	58.91472868	41.08527132	
	2.5	0.624	0.619	0.638	0.627	0.005686	81.00775194	18.99224806	
	1.25	0.763	0.749	0.758	0.756667	0.004096	97.76055125	2.239448751	
	0.625	0.773	0.778	0.771	0.774	0.002082	100	0	
	0.312	0.765	0.772	0.785	0.774	0.005859	100	0	
13 (5a)	10	0.021	0.018	0.02	0.019667	0.000882	2.540913006	97.45908699	3.09 ± 0.01
	5	0.147	0.115	0.126	0.129333	0.009387	16.70973299	83.29026701	
	2.5	0.372	0.389	0.393	0.384667	0.006438	49.69853575	50.30146425	
	1.25	0.716	0.745	0.732	0.731	0.008386	94.44444444	5.555555556	
	0.625	0.776	0.768	0.772	0.772	0.002309	99.74160207	0.258397933	
	0.312	0.769	0.777	0.773	0.773	0.002309	99.87080103	0.129198966	
14 (6a)	10	0.034	0.032	0.033	0.033	0.000577	4.263565891	95.73643411	1.69 ± 0.01
	5	0.027	0.031	0.036	0.031333	0.002603	4.048234281	95.95176572	
	2.5	0.084	0.072	0.079	0.078333	0.00348	10.1205857	89.8794143	
	1.25	0.537	0.595	0.573	0.568333	0.016905	73.42807924	26.57192076	
	0.625	0.773	0.777	0.771	0.773667	0.001764	99.95693368	0.043066322	
	0.312	0.764	0.772	0.777	0.771	0.003786	99.6124031	0.387596899	

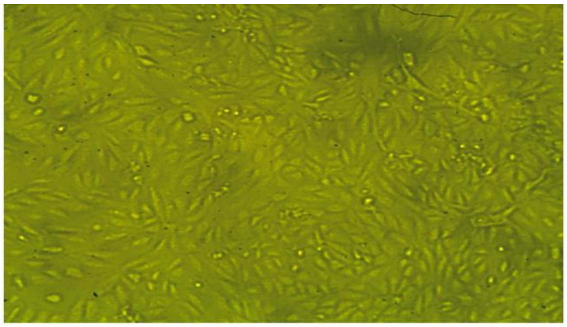

control
HT29 cells

Organism :	*Homo sapiens*, human
Tissue :	colon
Cell Type :	epithelial
Culture Properties :	adherent
Disease :	Adenocarcinoma; Colorectal
ATCC :	HTB-38

Effect of sample 1 on HT29 cells at different concentration

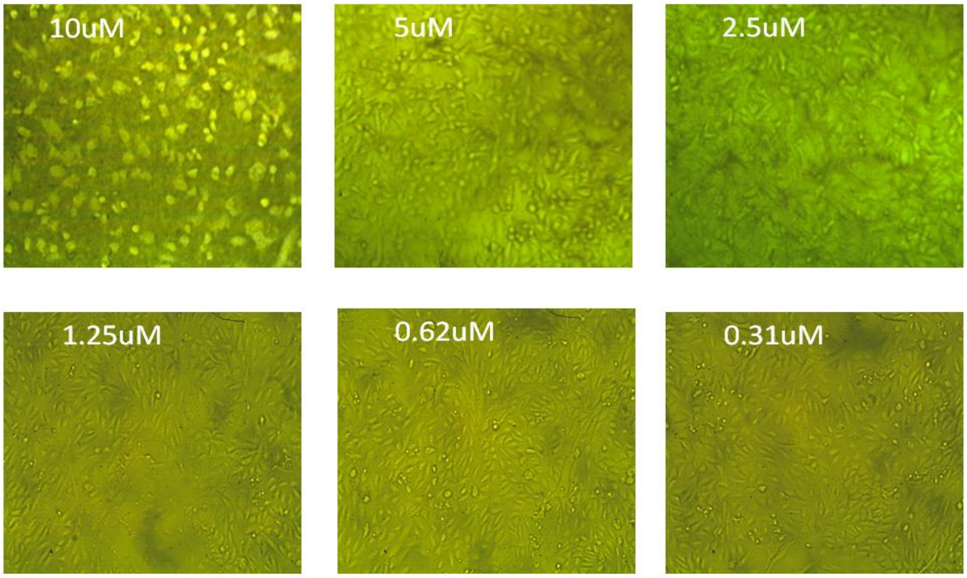

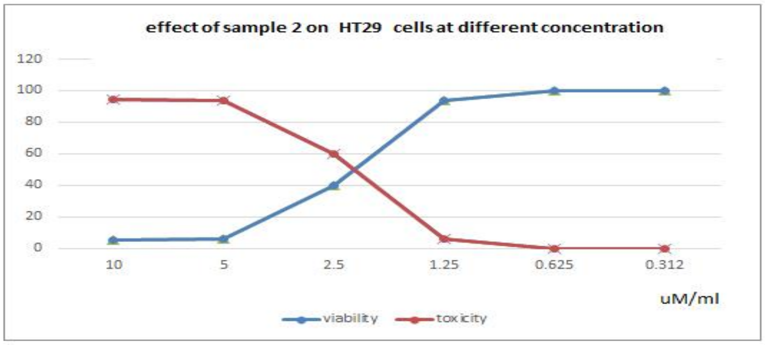

Effect of sample 2 on HT29 cells at different concentration

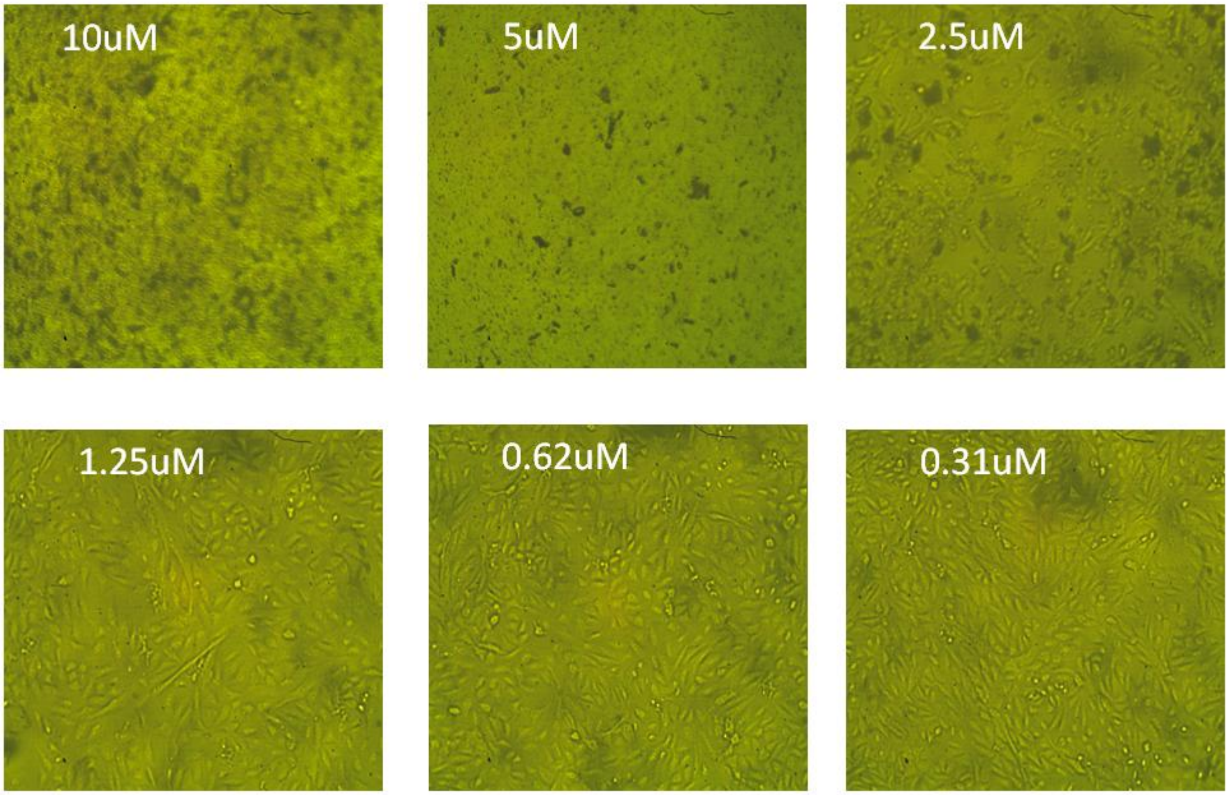

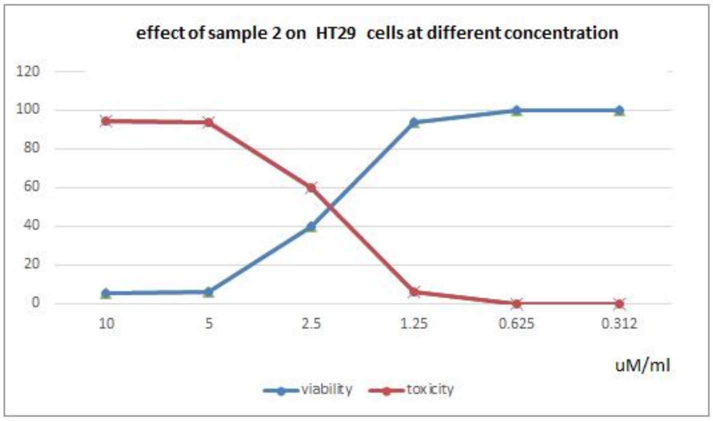

Effect of sample 3 on HT29 cells at different concentration

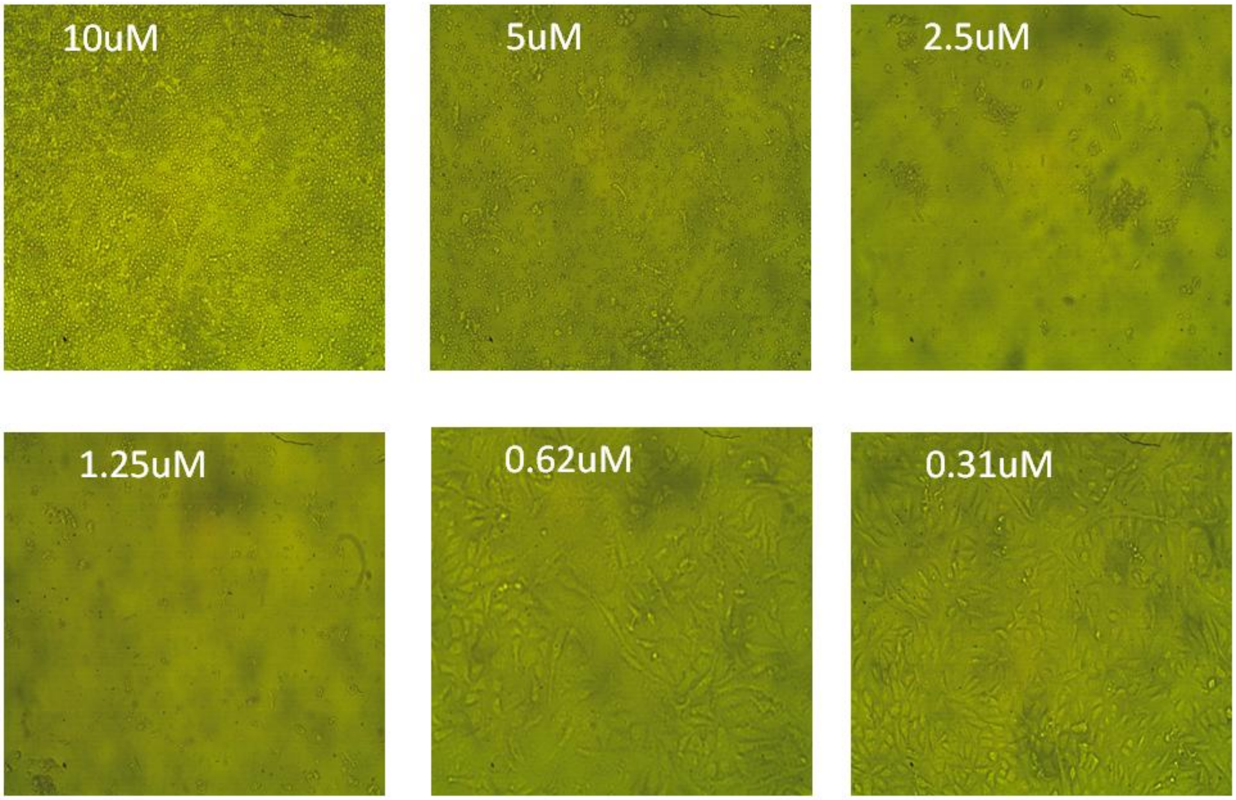

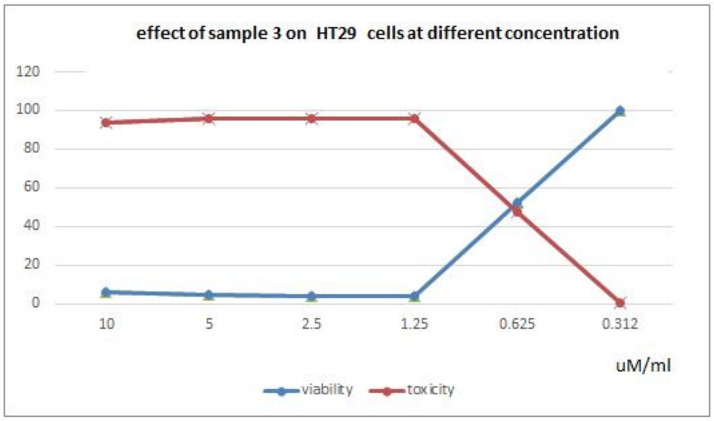

Effect of sample 6 on HT29 cells at different concentration

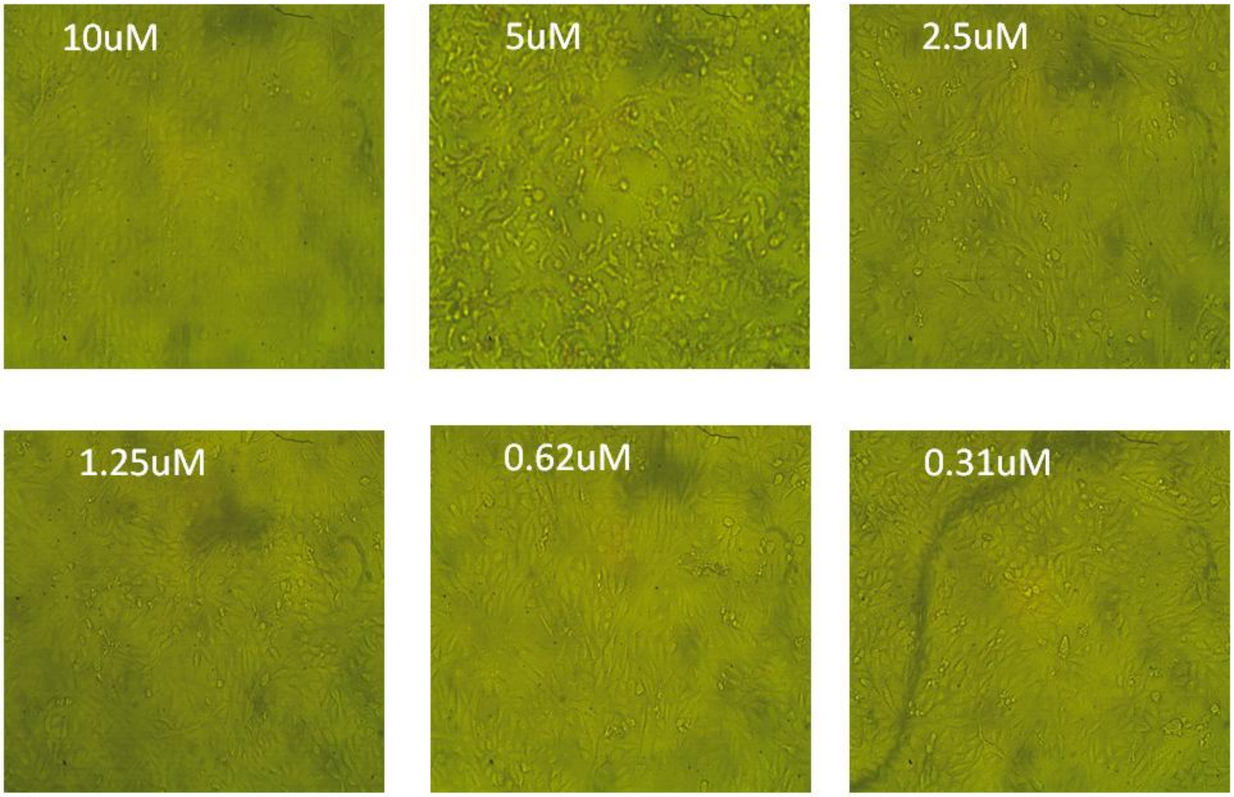

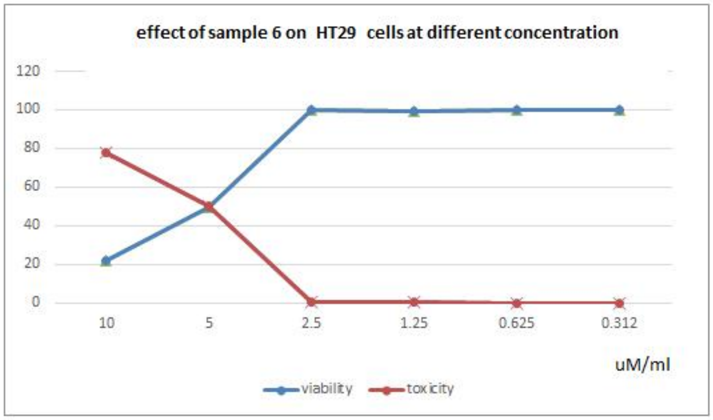

Effect of sample 7 on HT29 cells at different concentration

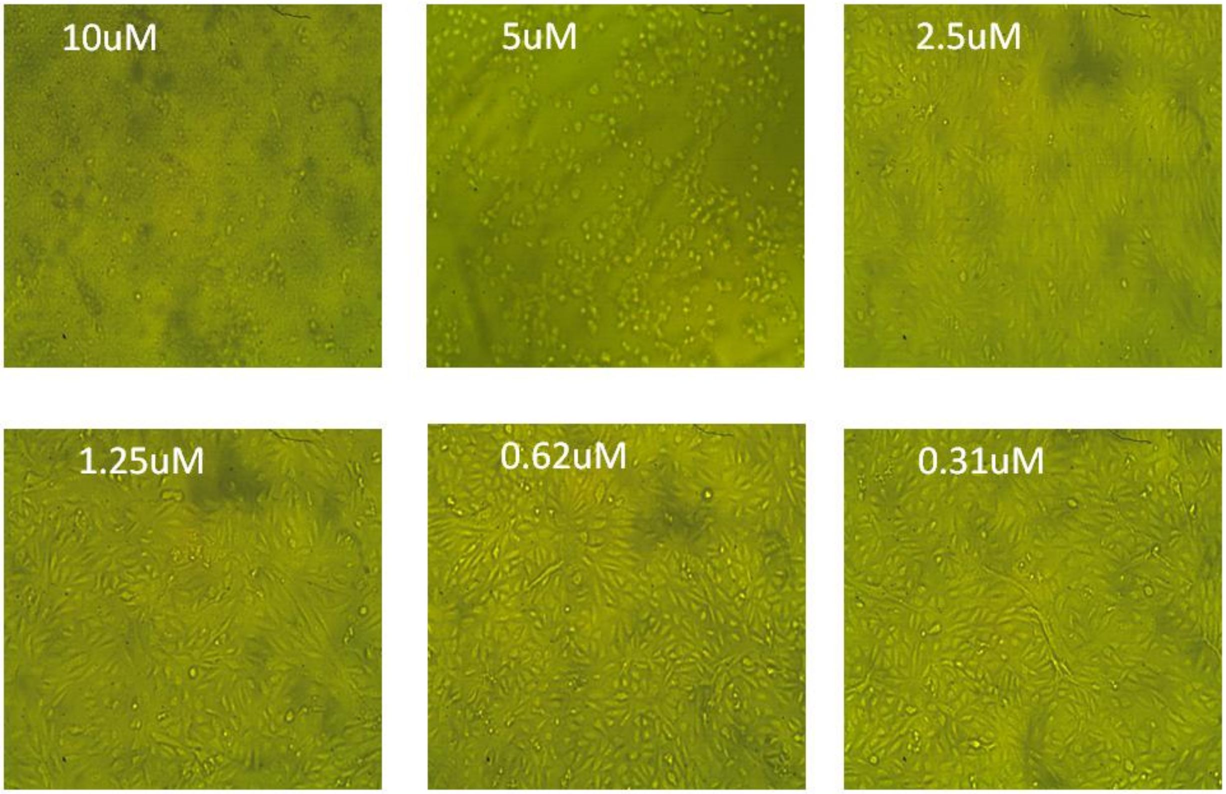

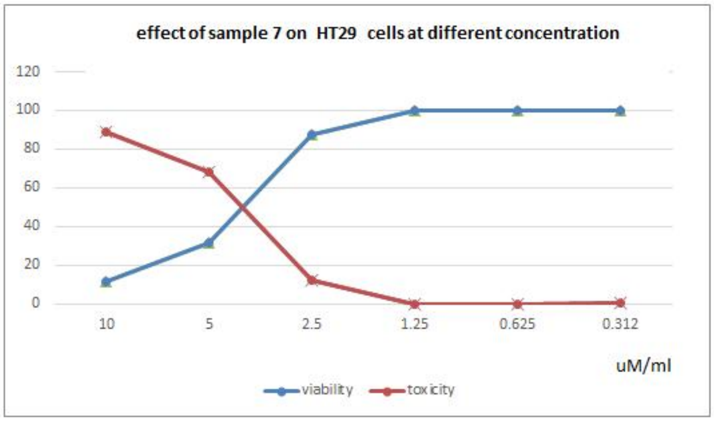

Effect of sample 8 on HT29 cells at different concentration

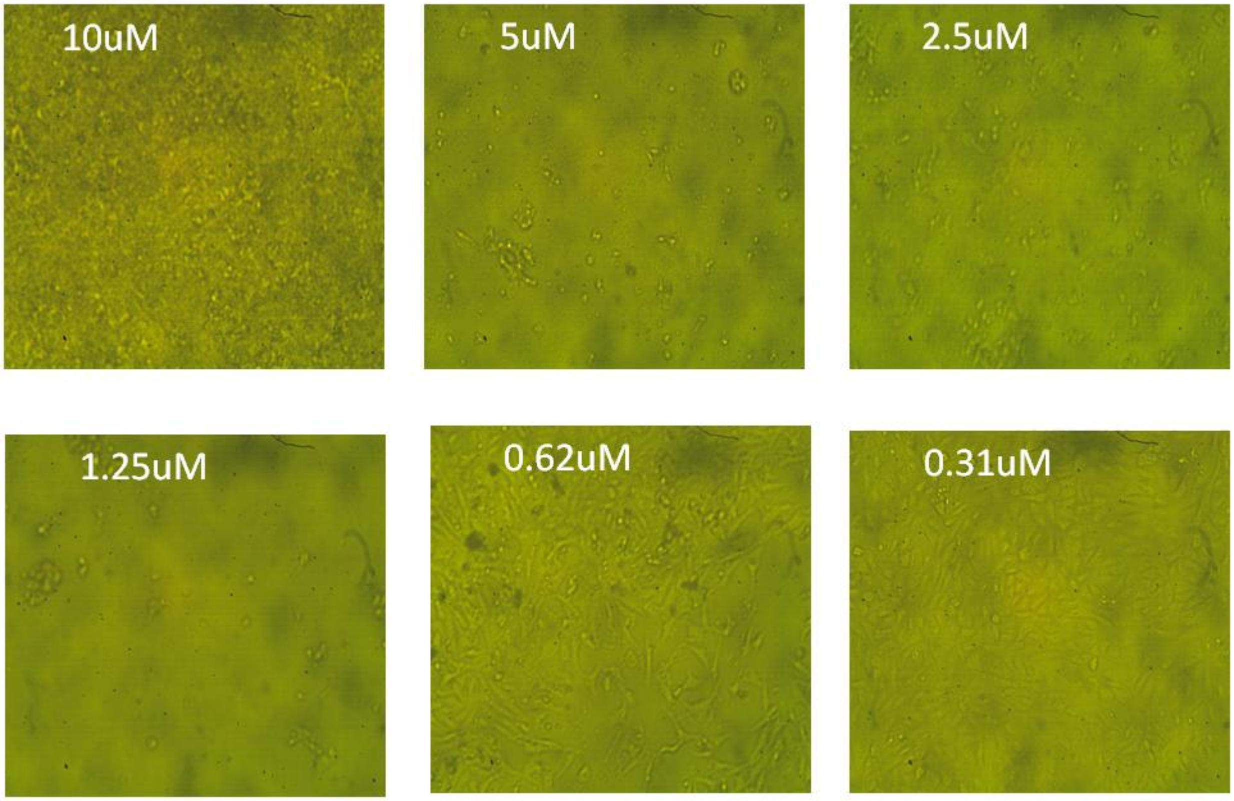

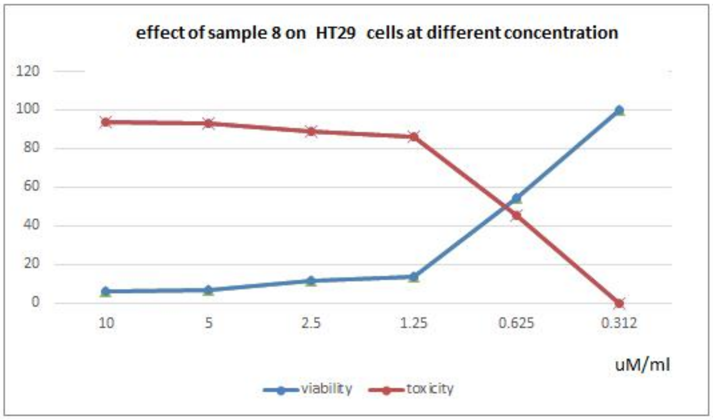

Effect of sample 9 on HT29 cells at different concentration

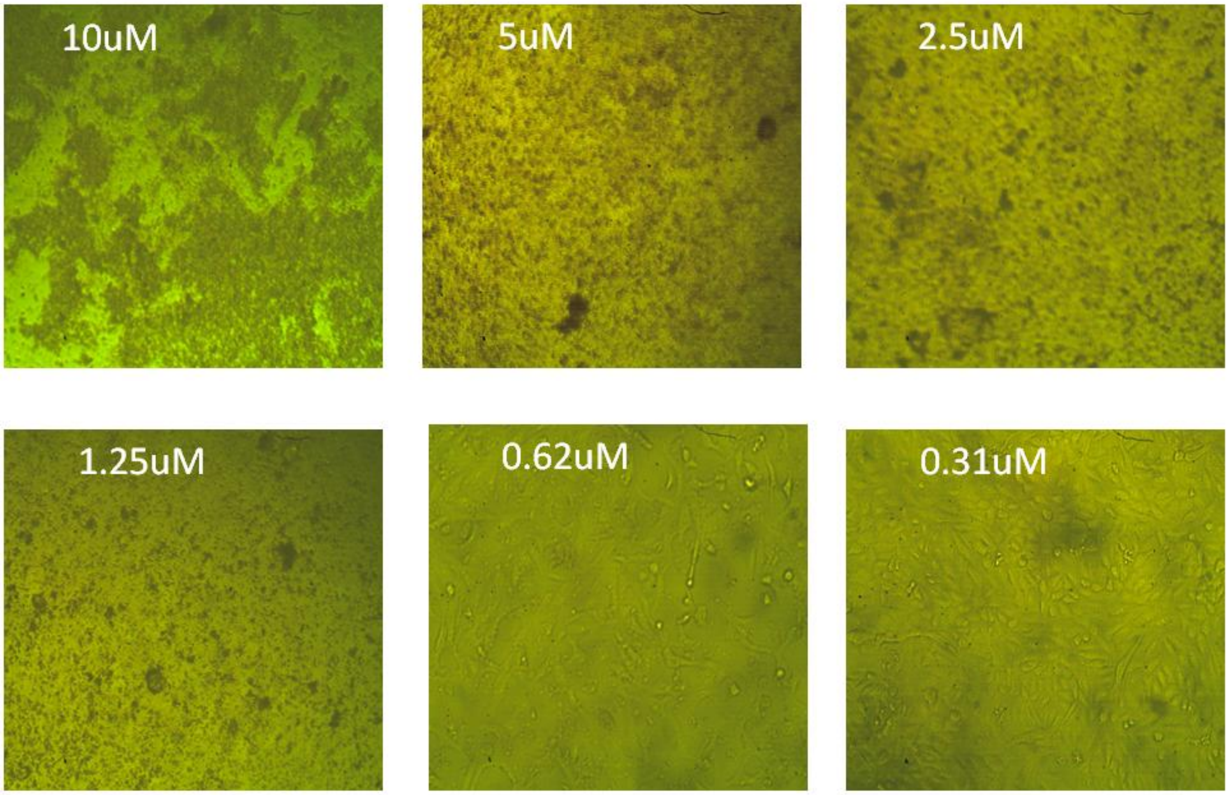

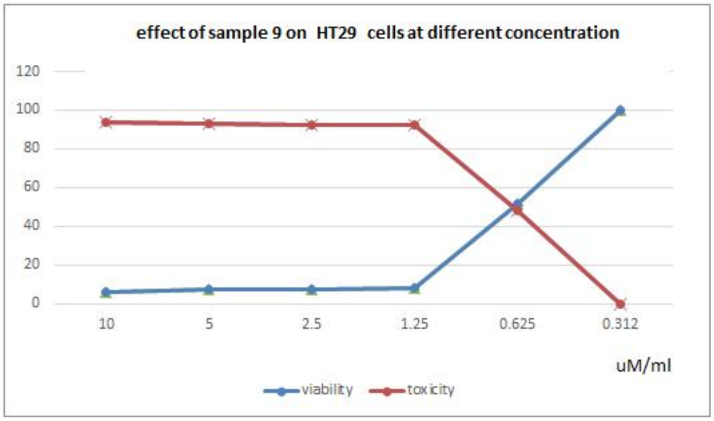

Effect of sample 12 on HT29 cells at different concentration

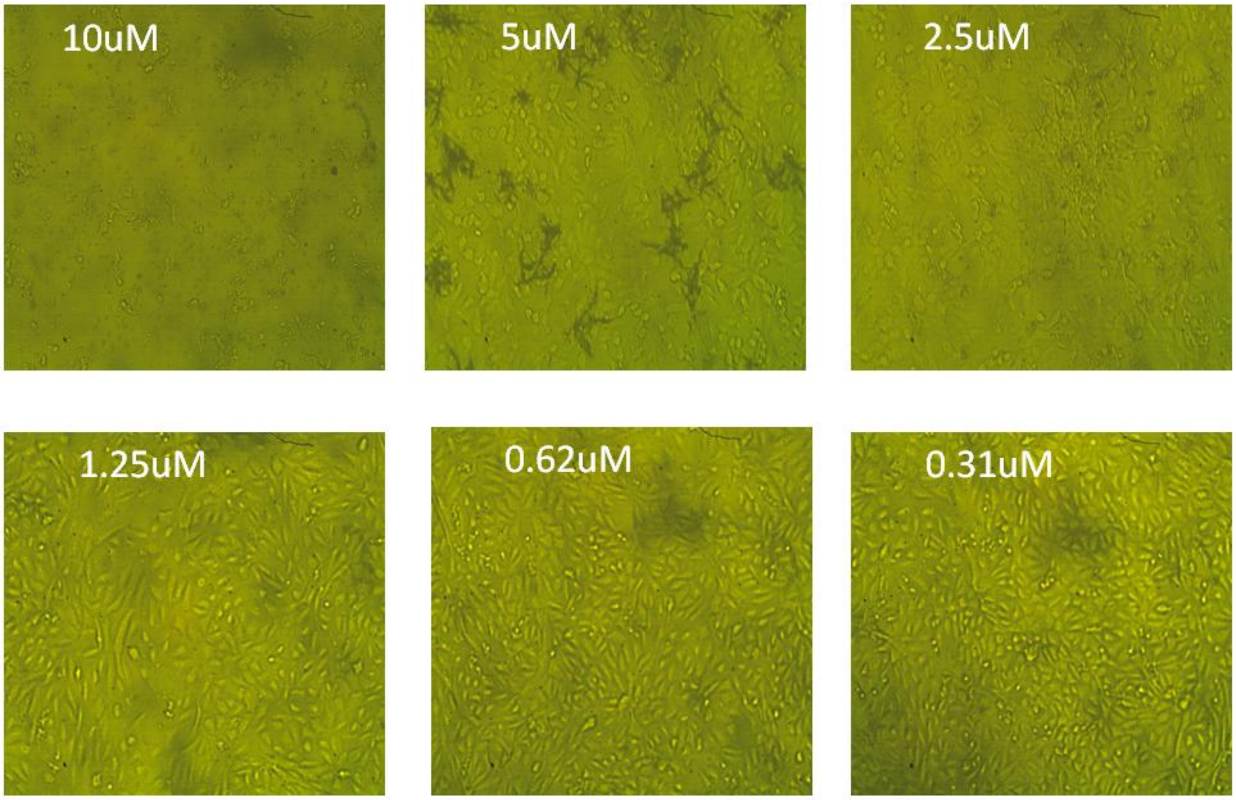

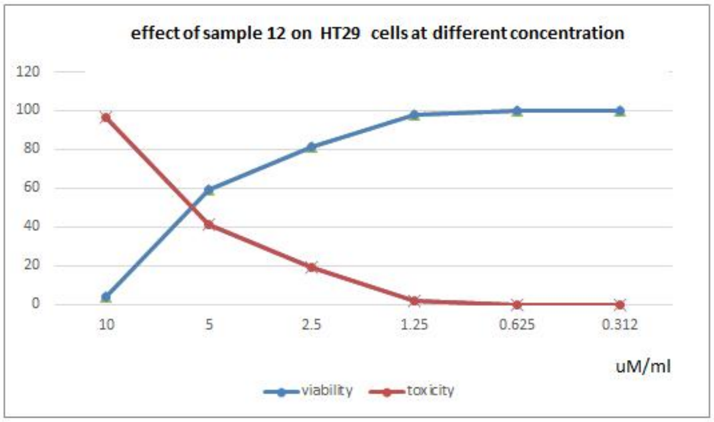

Effect of sample 13 on HT29 cells at different concentration

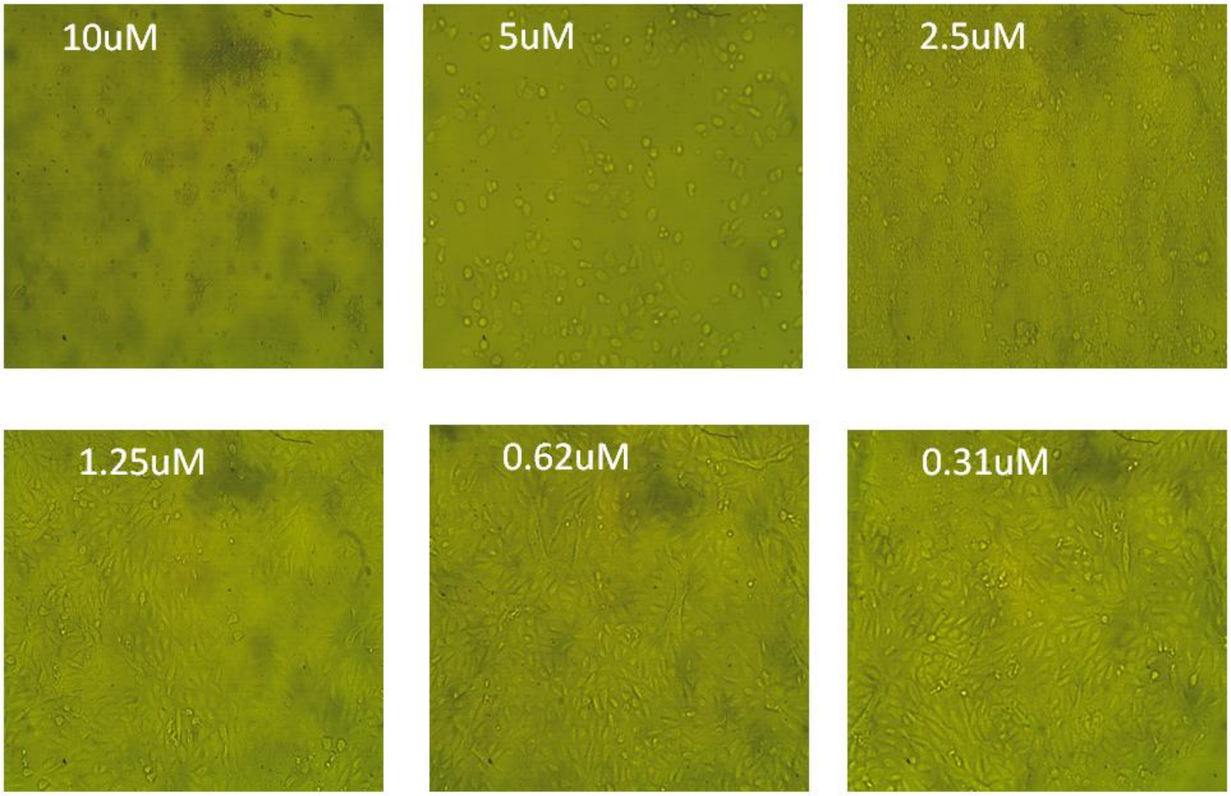

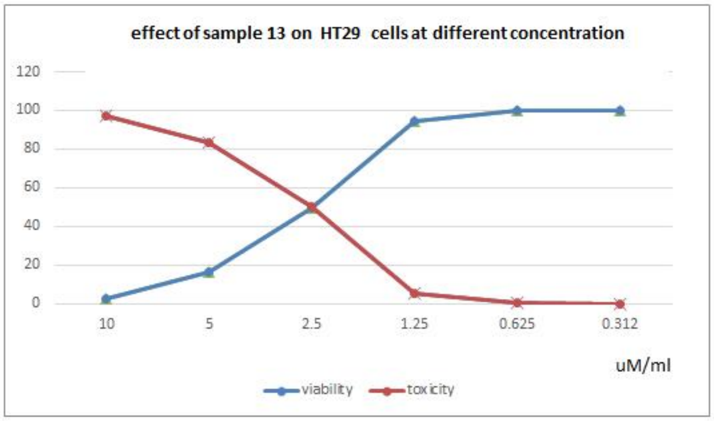

Effect of sample 14 on HT29 cells at different concentration

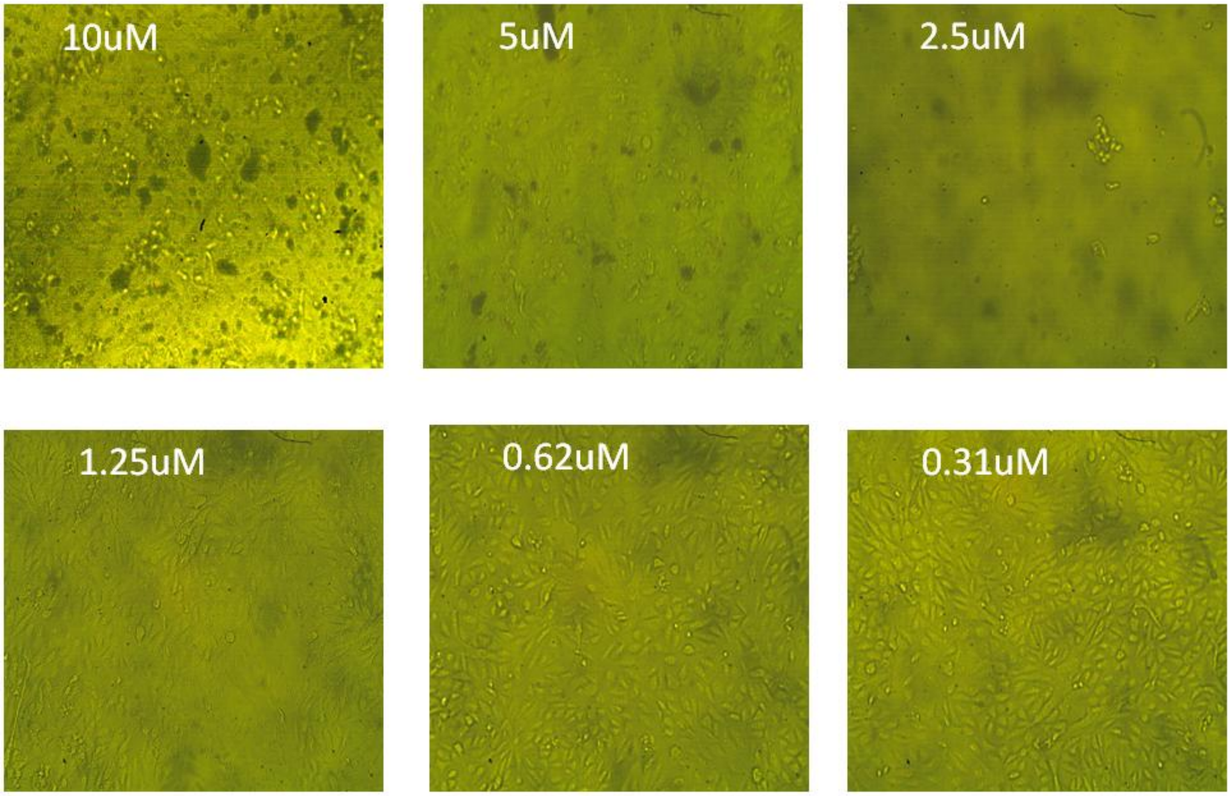

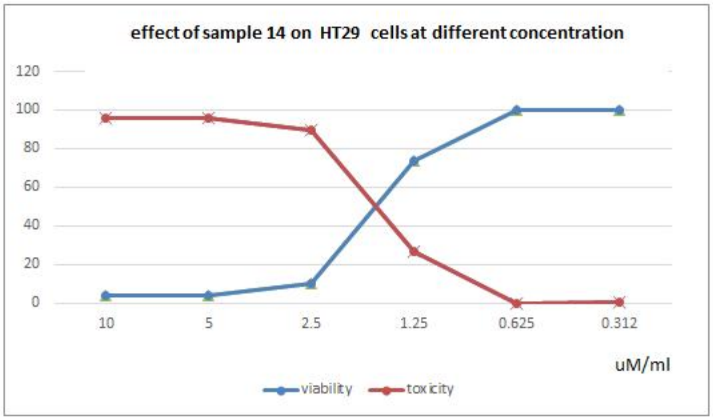

ID	uM/ ml	O.D			Mean O.D	±SE	Viability %	Toxicity %	IC50 ± SD
U937	-------	0.599	0.625	0.612	0.612	0.007506	100	0	uM
1 (5b)	10	0.1	0.084	0.115	0.099667	0.00895	16.28540305	83.71459695	6.29 ± 0.26
	5	0.326	0.286	0.311	0.307667	0.011667	50.27233115	49.72766885	
	2.5	0.61	0.587	0.623	0.606667	0.010525	99.12854031	0.871459695	
	1.25	0.607	0.62	0.602	0.609667	0.005364	99.61873638	0.381263617	
	0.625	0.617	0.611	0.606	0.611333	0.00318	99.89106754	0.108932462	
	0.312	0.6	0.621	0.614	0.611667	0.006173	99.94553377	0.054466231	
2 (5c)	10	0.088	0.091	0.083	0.087333	0.002333	14.27015251	85.72984749	6.17 ± 0.15
	5	0.313	0.283	0.289	0.295	0.009165	48.20261438	51.79738562	
	2.5	0.612	0.597	0.615	0.608	0.005568	99.34640523	0.653594771	
	1.25	0.62	0.603	0.608	0.610333	0.005044	99.72766885	0.272331155	
	0.625	0.593	0.622	0.612	0.609	0.008505	99.50980392	0.490196078	
	0.312	0.611	0.609	0.61	0.61	0.000577	99.67320261	0.326797386	
3 (6b)	10	0.05	0.062	0.066	0.059333	0.004807	9.694989107	90.30501089	0.97 ± 0.01
	5	0.067	0.064	0.06	0.063667	0.002028	10.40305011	89.59694989	
	2.5	0.072	0.069	0.083	0.074667	0.004256	12.20043573	87.79956427	
	1.25	0.153	0.178	0.183	0.171333	0.00928	27.9956427	72.0043573	
	0.625	0.463	0.489	0.471	0.474333	0.007688	77.50544662	22.49455338	
	0.312	0.599	0.605	0.615	0.606333	0.004667	99.07407407	0.925925926	
6 (8)	10	0.157	0.182	0.168	0.169	0.007234	27.61437908	72.38562092	7.77 ± 0.04
	5	0.488	0.472	0.501	0.487	0.008386	79.5751634	20.4248366	
	2.5	0.62	0.604	0.608	0.610667	0.004807	99.78213508	0.217864924	
	1.25	0.611	0.617	0.607	0.611667	0.002906	99.94553377	0.054466231	
	0.625	0.615	0.603	0.606	0.608	0.003606	99.34640523	0.653594771	
	0.312	0.601	0.615	0.613	0.609667	0.004372	99.61873638	0.381263617	
7 (9)	10	0.095	0.136	0.124	0.118333	0.01217	19.33551198	80.66448802	4.42 ± 0.18
	5	0.261	0.237	0.273	0.257	0.010583	41.99346405	58.00653595	
	2.5	0.473	0.462	0.478	0.471	0.004726	76.96078431	23.03921569	
	1.25	0.603	0.589	0.618	0.603333	0.008373	98.583878	1.416122004	
	0.625	0.613	0.616	0.607	0.612	0.002646	100	0	
	0.312	0.609	0.609	0.614	0.610667	0.001667	99.78213508	0.217864924	

ID	uM/ ml	O.D			Mean O.D	±SE	Viability %	Toxicity %	IC50 ± SD
U937	-------	0.599	0.625	0.612	0.612	0.007506	100	0	uM
8 (7)	10	0.061	0.078	0.071	0.07	0.004933	11.4379085	88.5620915	1.85 ± 0.04
	5	0.165	0.196	0.183	0.181333	0.008988	29.62962963	70.37037037	
	2.5	0.244	0.217	0.267	0.242667	0.014449	39.65141612	60.34858388	
	1.25	0.317	0.365	0.336	0.339333	0.013956	55.44662309	44.55337691	
	0.625	0.513	0.567	0.542	0.540667	0.015603	88.34422658	11.65577342	
	0.312	0.574	0.569	0.583	0.575333	0.004096	94.0087146	5.991285403	
9 (4)	10	0.046	0.052	0.047	0.048333	0.001856	7.897603486	92.10239651	0.95 ± 0
	5	0.05	0.043	0.044	0.045667	0.002186	7.461873638	92.53812636	
	2.5	0.056	0.043	0.048	0.049	0.003786	8.006535948	91.99346405	
	1.25	0.137	0.163	0.155	0.151667	0.007688	24.78213508	75.21786492	
	0.625	0.493	0.521	0.51	0.508	0.008145	83.00653595	16.99346405	
	0.312	0.61	0.584	0.593	0.595667	0.007623	97.33115468	2.668845316	
12 (10)	10	0.188	0.23	0.222	0.213333	0.012875	34.8583878	65.1416122	8.37 ± 0.23
	5	0.495	0.524	0.541	0.52	0.013429	84.96732026	15.03267974	
	2.5	0.572	0.599	0.587	0.586	0.00781	95.75163399	4.248366013	
	1.25	0.613	0.611	0.611	0.611667	0.000667	99.94553377	0.054466231	
	0.625	0.608	0.618	0.607	0.611	0.003512	99.83660131	0.163398693	
	0.312	0.598	0.616	0.612	0.608667	0.005457	99.45533769	0.544662309	
13 (5a)	10	0.073	0.068	0.092	0.077667	0.007311	12.69063181	87.30936819	6.08 ± 0.11
	5	0.352	0.333	0.326	0.337	0.007767	55.06535948	44.93464052	
	2.5	0.548	0.561	0.538	0.549	0.006658	89.70588235	10.29411765	
	1.25	0.593	0.623	0.612	0.609333	0.008762	99.56427015	0.435729847	
	0.625	0.6	0.604	0.611	0.605	0.003215	98.85620915	1.14379085	
	0.312	0.616	0.604	0.608	0.609333	0.003528	99.56427015	0.435729847	
14 (6a)	10	0.053	0.062	0.067	0.060667	0.004096	9.912854031	90.08714597	1.12 ± 0.02
	5	0.073	0.088	0.082	0.081	0.004359	13.23529412	86.76470588	
	2.5	0.083	0.106	0.078	0.089	0.008622	14.54248366	85.45751634	
	1.25	0.241	0.238	0.261	0.246667	0.007219	40.30501089	59.69498911	
	0.625	0.583	0.579	0.603	0.588333	0.007424	96.1328976	3.867102397	
	0.312	0.614	0.602	0.604	0.606667	0.003712	99.12854031	0.871459695	

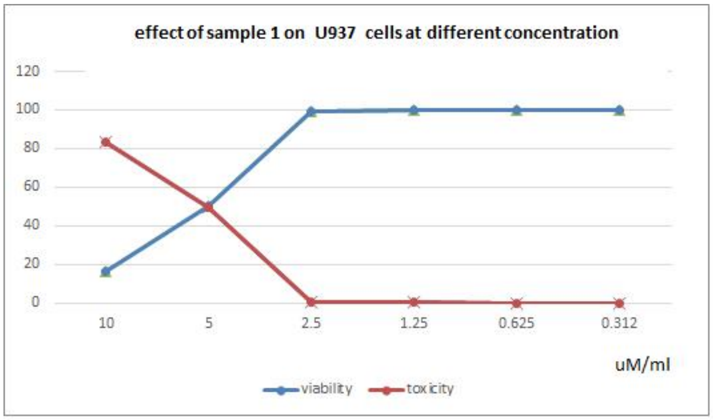
effect of sample 1 on U937 cells at different concentration
120
100
80
60
40
20
0
10
5
2.5
1.25
0.625
0.312
uM/ml
viability
toxicity

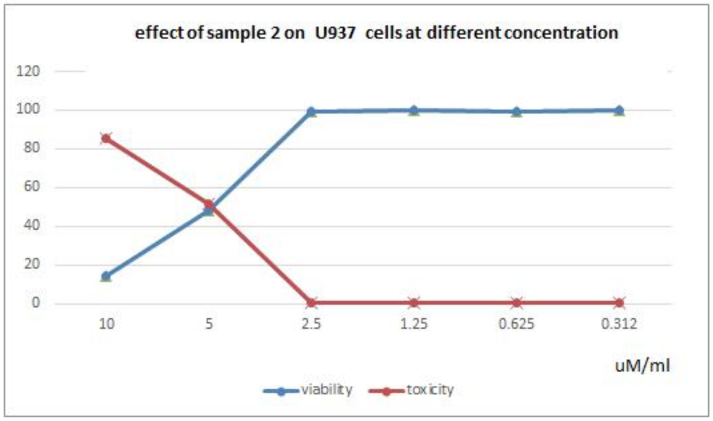
effect of sample 2 on U937 cells at different concentration
120
100
80
60
40
20
0
10
5
2.5
1.25
0.625
0.312
uM/ml
viability
toxicity

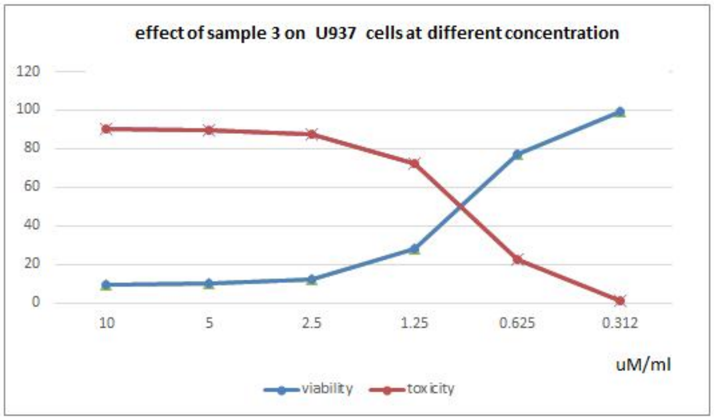
effect of sample 3 on U937 cells at different concentration
120
100
80
60
40
20
0
10
5
2.5
1.25
0.625
0.312
uM/ml
viability
toxicity

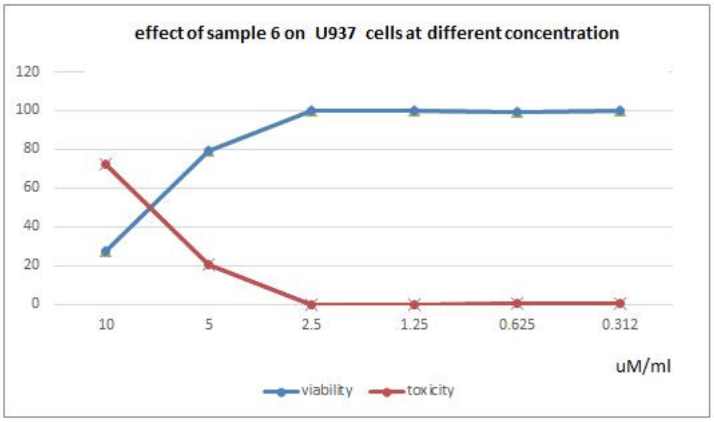
effect of sample 6 on U937 cells at different concentration
120
100
80
60
40
20
0
10
5
2.5
1.25
0.625
0.312
uM/ml
viability
toxicity

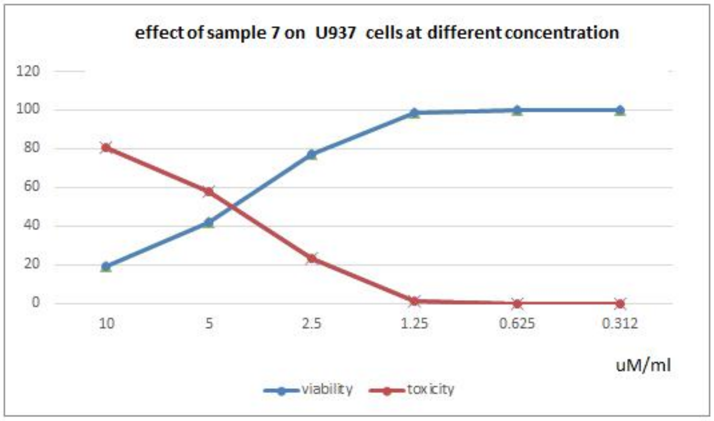
effect of sample 7 on U937 cells at different concentration
120
100
80
60
40
20
0
10
5
2.5
1.25
0.625
0.312
uM/ml
viability
toxicity

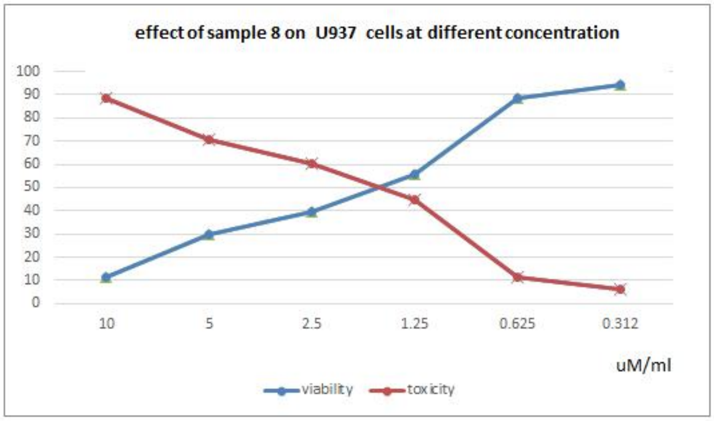
effect of sample 8 on U937 cells at different concentration
100
90
80
70
60
50
40
30
20
10
0
10
5
2.5
1.25
0.625
0.312
uM/ml
viability
toxicity

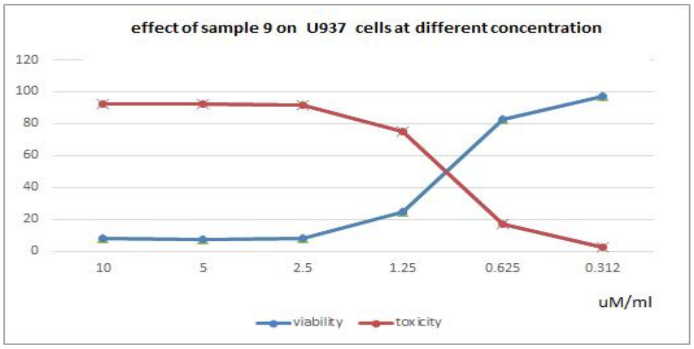
effect of sample 9 on U937 cells at different concentration
120
100
80
60
40
20
0
10
5
2.5
1.25
0.625
0.312
uM/ml
viability
toxicity

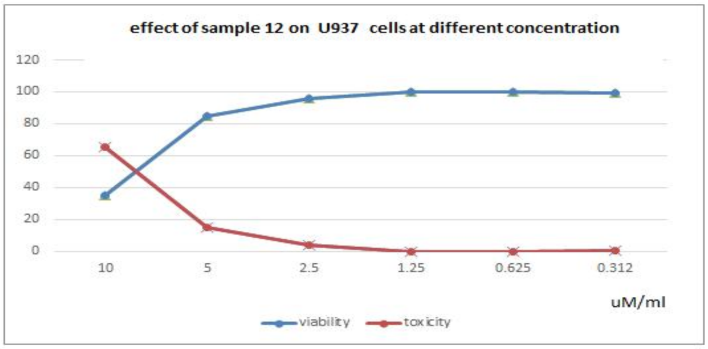
effect of sample 12 on U937 cells at different concentration
120
100
80
60
40
20
0
10
5
2.5
1.25
0.625
0.312
uM/ml
viability
toxicity

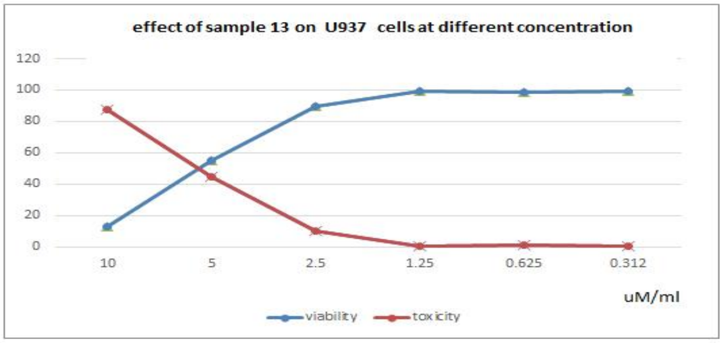
effect of sample 13 on U937 cells at different concentration
120
100
80
60
40
20
0
10
5
2.5
1.25
0.625
0.312
uM/ml
viability
toxicity

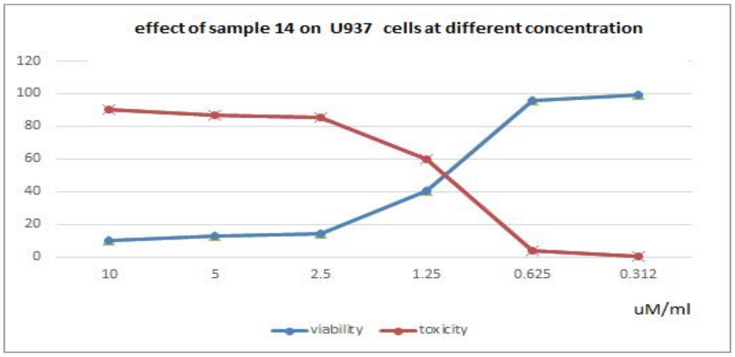
effect of sample 14 on U937 cells at different concentration
120
100
80
60
40
20
0
10
5
2.5
1.25
0.625
0.312
uM/ml
viability
toxicity

Effect of Methotrexate against MDA-MB-231, HT29 and u937 cells

ID	Conc. uM	O.D			Mean O.D	ST.E	Viability %	Toxicity %	IC50
MDA-M**B-231**	--------	0.768	0.745	0.752	0.755	0.006807	100	0	uM
methotrexate.	10	0.086	0.094	0.088	0.089333	0.002404	11.83222958	88.16777042	2.79 ± 0.06
	5	0.142	0.156	0.143	0.147	0.004509	19.47019868	80.52980132	
	2.5	0.32	0.326	0.314	0.32	0.003464	42.38410596	57.61589404	
	1.25	0.621	0.617	0.609	0.615667	0.003528	81.54525386	18.45474614	
	0.625	0.759	0.751	0.75	0.753333	0.002848	99.77924945	0.220750552	
	0.312	0.766	0.743	0.748	0.752333	0.006984	99.64679912	0.353200883	
	0.156	0.756	0.751	0.752	0.753	0.001528	99.73509934	0.264900662	
	0.078	0.759	0.748	0.756	0.754333	0.003283	99.91169978	0.088300221	

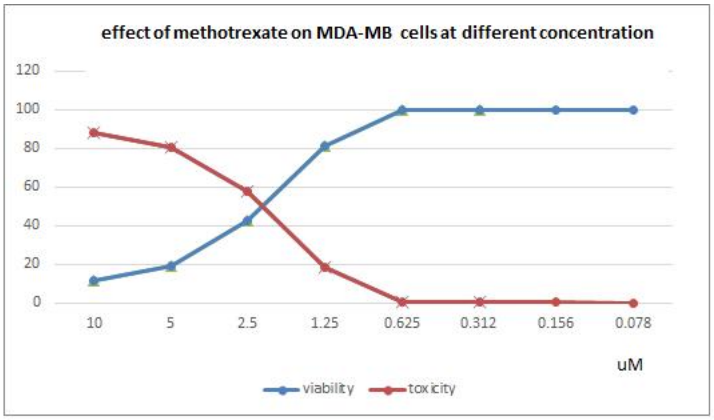

ID	Conc. uM	O.D			Mean O.D	ST.E	Viability %	Toxicity %	IC50
HT29	--------	0.744	0.728	0.721	0.731	0.006807	100	0	uM
methotrexate.	10	0.017	0.019	0.017	0.017667	0.000667	2.416780666	97.58321933	0.99 ± 0.02
	5	0.019	0.018	0.019	0.018667	0.000333	2.553579571	97.44642043	
	2.5	0.074	0.072	0.077	0.074333	0.001453	10.16871865	89.83128135	
	1.25	0.231	0.256	0.238	0.241667	0.007446	33.05973552	66.94026448	
	0.625	0.534	0.527	0.541	0.534	0.004041	73.0506156	26.9493844	
	0.312	0.728	0.731	0.729	0.729333	0.000882	99.77200182	0.227998176	
	0.156	0.719	0.722	0.732	0.724333	0.00393	99.0880073	0.911992704	
	0.078	0.734	0.72	0.728	0.727333	0.004055	99.49840401	0.501595987	

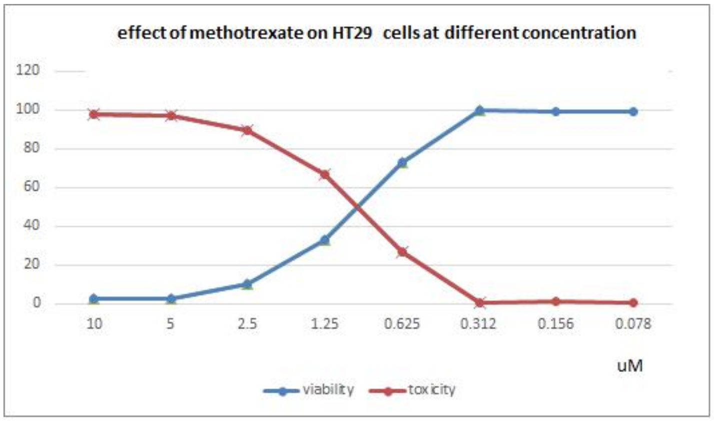

ID	Conc. uM	O.D			Mean O.D	ST.E	Viability %	Toxicity %	IC50
U937	--------	0.657	0.666	0.669	0.664	0.003606	100	0	uM
methotrexate.	10	0.045	0.052	0.055	0.050667	0.002963	7.630522088	92.36947791	1.22 ± 0.02
	5	0.056	0.063	0.048	0.055667	0.004333	8.383534137	91.61646586	
	2.5	0.216	0.214	0.198	0.209333	0.005696	31.52610442	68.47389558	
	1.25	0.324	0.309	0.314	0.315667	0.00441	47.54016064	52.45983936	
	0.625	0.643	0.647	0.64	0.643333	0.002028	96.8875502	3.112449799	
	0.312	0.653	0.66	0.657	0.656667	0.002028	98.89558233	1.104417671	
	0.156	0.663	0.667	0.661	0.663667	0.001764	99.9497992	0.050200803	
	0.078	0.665	0.666	0.659	0.663333	0.002186	99.89959839	0.100401606	

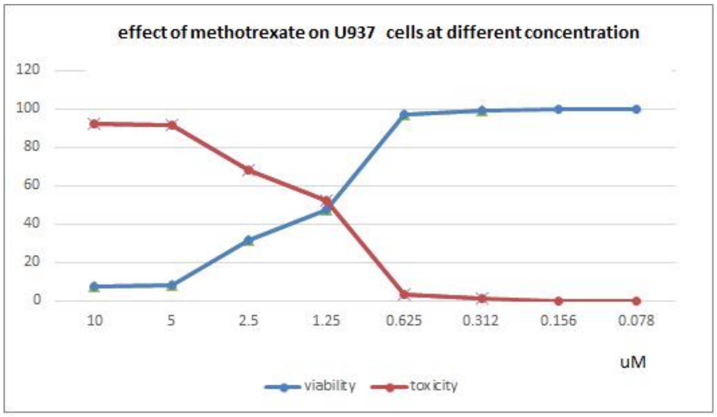

Viability assay

(Effect of compounds 4, 6a,b, and 7 against normal Vero cells)

Test code: T-2-023-16

ID	uM/ ml	O.D			Mean O.D	±SE	Viability %	Toxicity %	IC50 ± SD
Vero	-------	0.844	0.835	0.826	0.835	0.005196	100	0	uM
3 (6b)	10	0.053	0.046	0.055	0.051333	0.002728	6.147704591	93.85229541	2.06 ± 0.03
	5	0.109	0.094	0.118	0.107	0.007	12.81437126	87.18562874	
	2.5	0.32	0.301	0.341	0.320667	0.011552	38.40319361	61.59680639	
	1.25	0.599	0.589	0.572	0.586667	0.007881	70.25948104	29.74051896	
	0.625	0.821	0.843	0.819	0.827667	0.007688	99.12175649	0.878243513	
	0.312	0.84	0.823	0.819	0.827333	0.006438	99.08183633	0.918163673	
8 (7)	10	0.073	0.097	0.094	0.088	0.00755	10.53892216	89.46107784	3.92 ± 0.08
	5	0.243	0.288	0.261	0.264	0.013077	31.61676647	68.38323353	
	2.5	0.618	0.598	0.624	0.613333	0.00786	73.45309381	26.54690619	
	1.25	0.811	0.809	0.825	0.815	0.005033	97.60479042	2.395209581	
	0.625	0.832	0.841	0.827	0.833333	0.004096	99.8003992	0.199600798	
	0.312	0.83	0.844	0.821	0.831667	0.006692	99.6007984	0.399201597	
9 (4)	10	0.018	0.019	0.02	0.019	0.000577	2.275449102	97.7245509	1.73 ± 0.03
	5	0.065	0.049	0.052	0.055333	0.00491	6.626746507	93.37325349	
	2.5	0.218	0.183	0.191	0.197333	0.010588	23.63273453	76.36726547	
	1.25	0.533	0.529	0.531	0.531	0.001155	63.59281437	36.40718563	
	0.625	0.785	0.759	0.783	0.775667	0.008353	92.89421158	7.105788423	
	0.312	0.842	0.835	0.828	0.835	0.004041	100	0	
14 (6a)	10	0.037	0.041	0.037	0.038333	0.001333	4.590818363	95.40918164	1.95 ± 0.05
	5	0.083	0.1	0.108	0.097	0.007371	11.61676647	88.38323353	
	2.5	0.227	0.193	0.251	0.223667	0.016826	26.78642715	73.21357285	
	1.25	0.684	0.718	0.693	0.698333	0.010171	83.63273453	16.36726547	
	0.625	0.839	0.821	0.826	0.828667	0.005364	99.24151697	0.758483034	
	0.312	0.847	0.822	0.831	0.833333	0.007311	99.8003992	0.199600798	

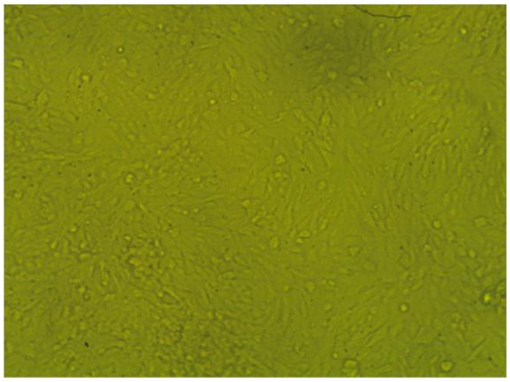

control
vero cells

Organism : *Cercopithecus aethiops*
Tissue : kidney
Cell Type : epithelial
Culture Properties : adherent
Disease : normal
ATCC : CCL-81

Effect of sample 3 on vero cells at different concentration

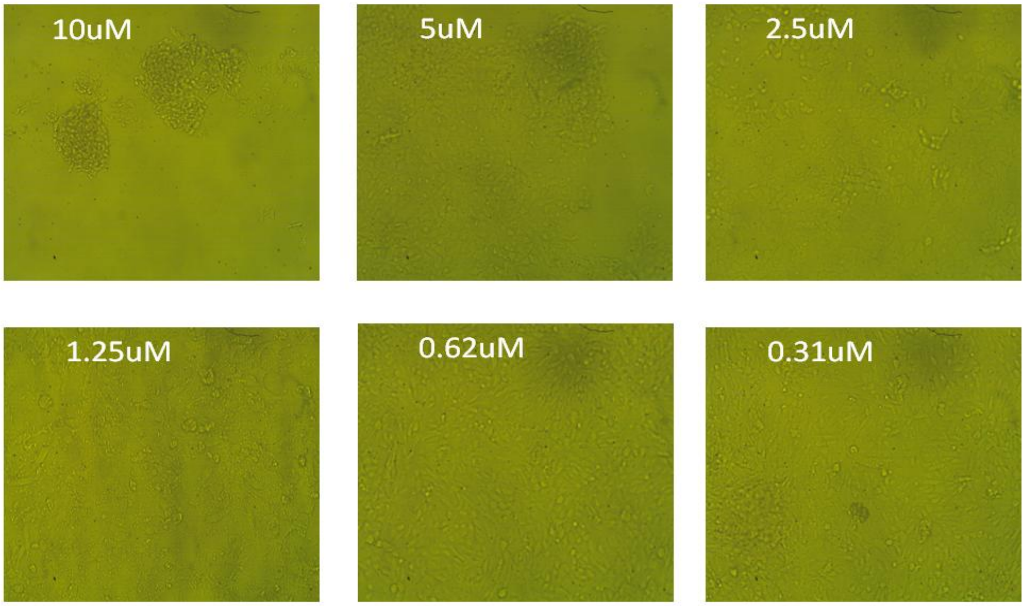

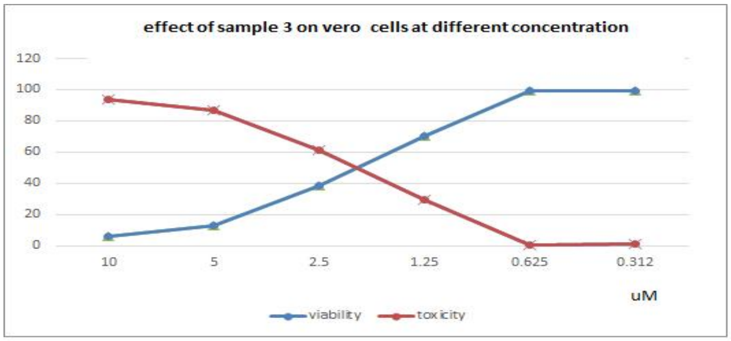

Effect of sample 8 on vero cells at different concentration

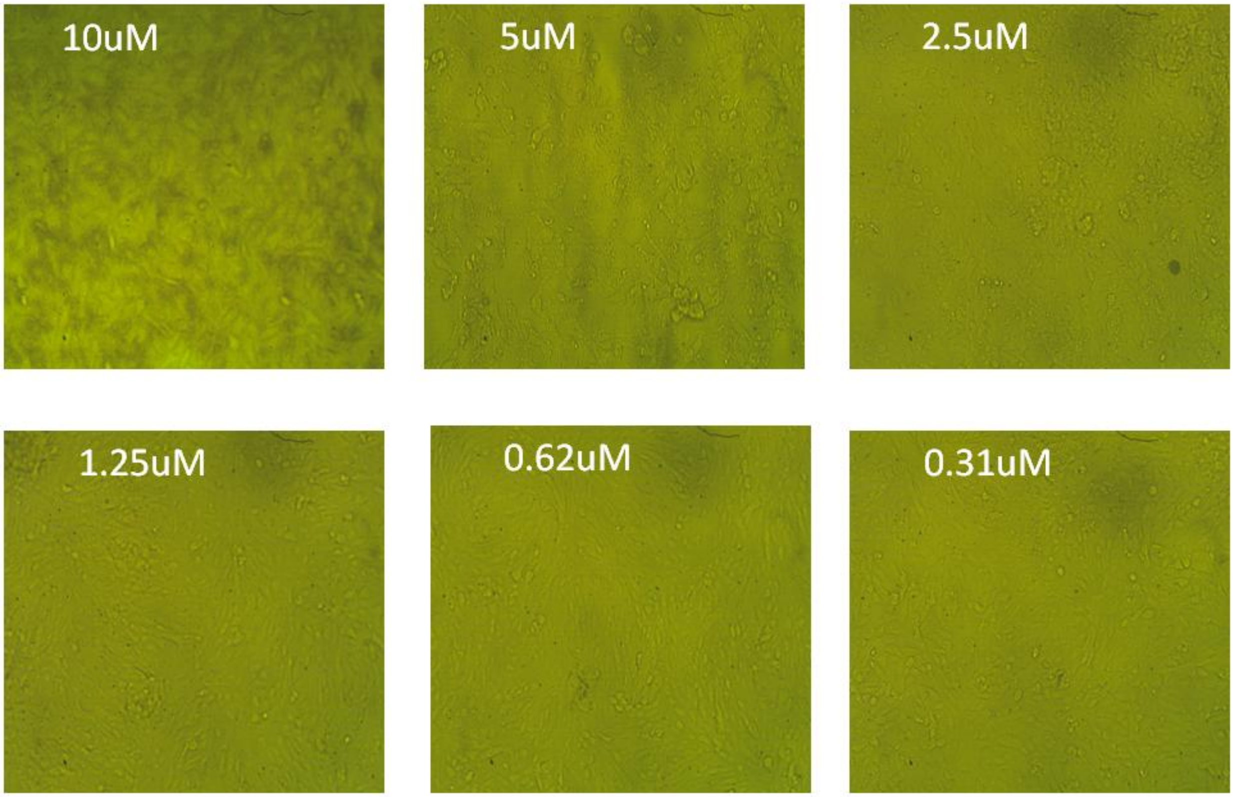

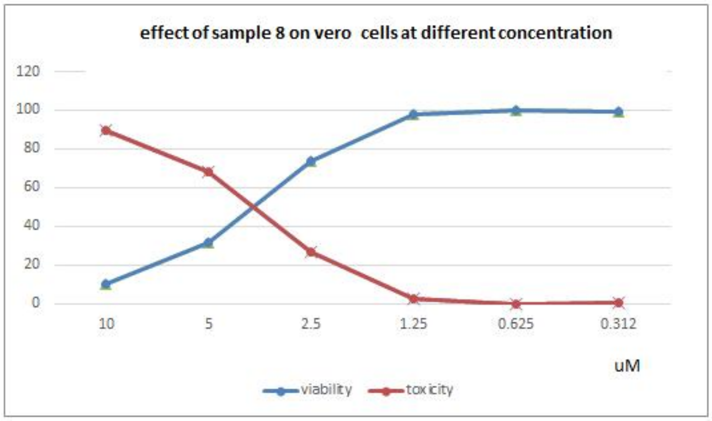

Effect of sample 9 on vero cells at different concentration

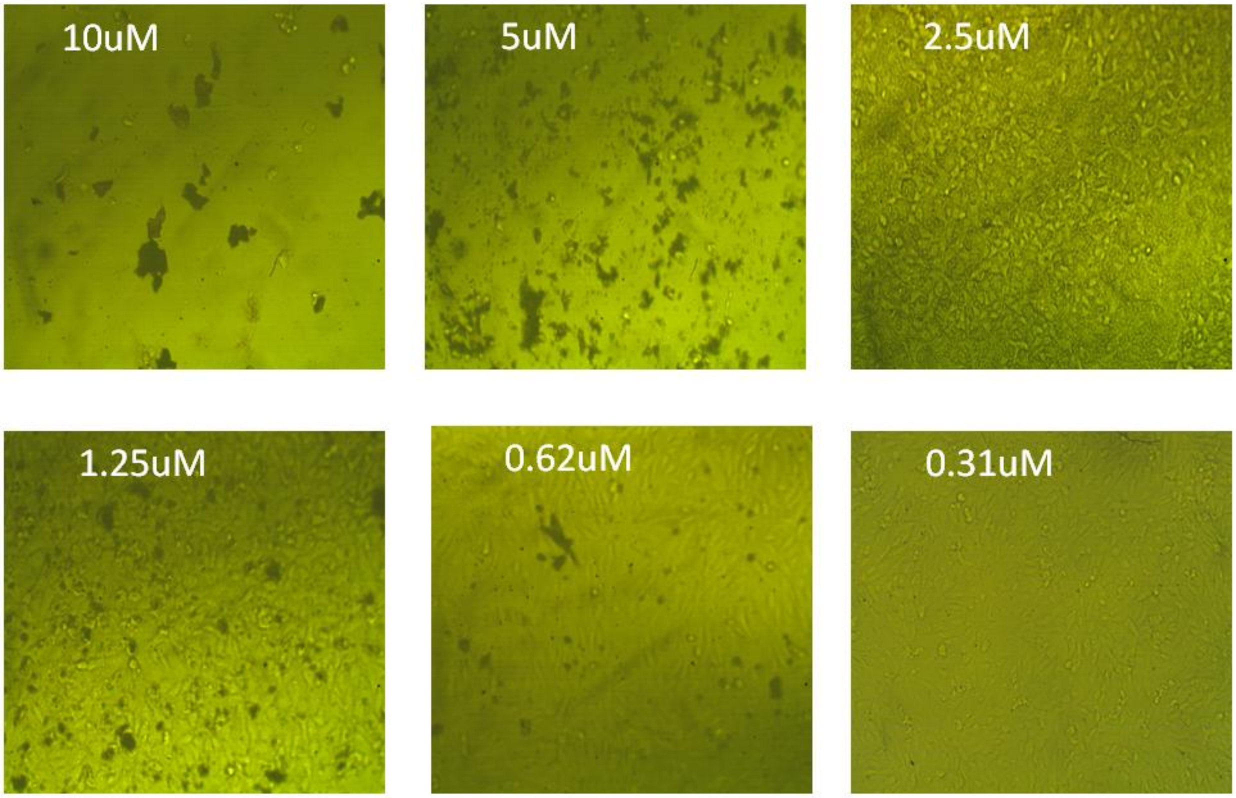

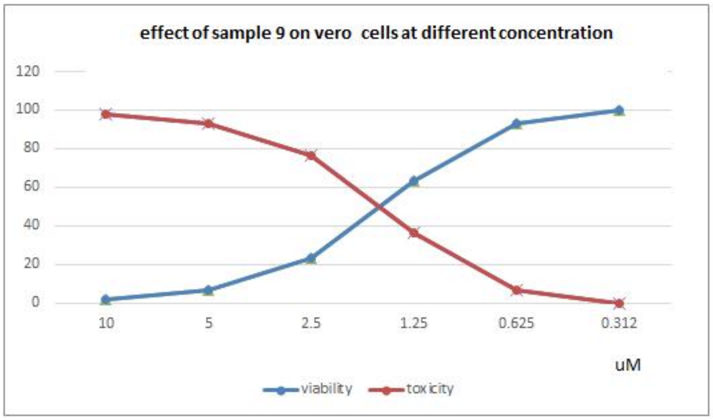

Effect of sample 14 on vero cells at different concentration

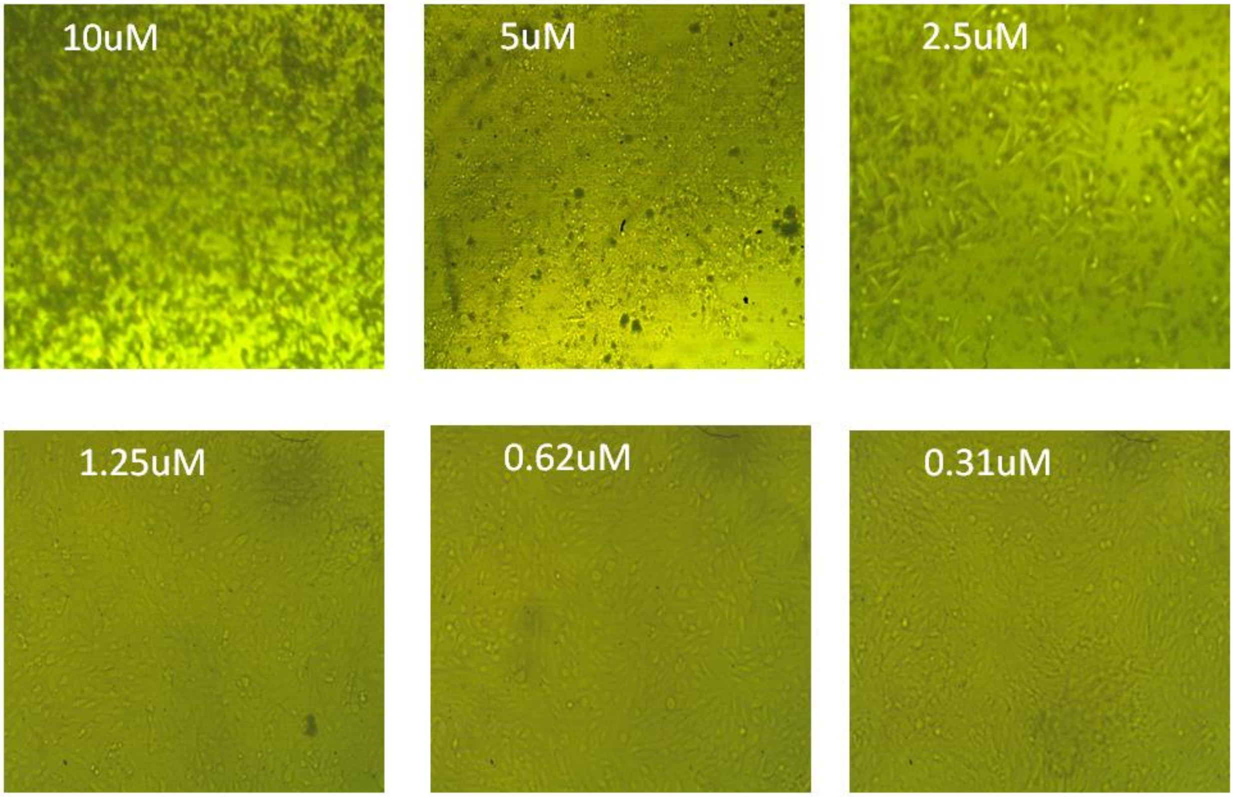

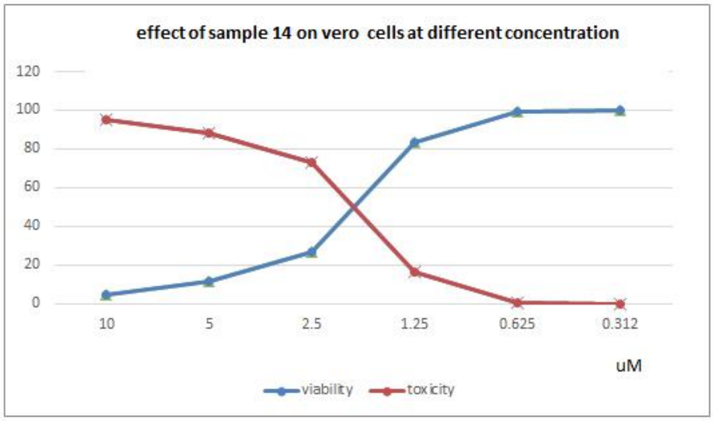

Effect of Methotrexate against Vero cells

Test code: T- methotrexate

ID	Conc. uM	O.D			Mean O.D	ST.E	Viability %	Toxicity %	IC50
Vero	--------	0.761	0.779	0.758	0.766	0.006557	100	0	uM
methotrexate.	10	0.401	0.376	0.377	0.384667	0.008172	50.2175805	49.7824195	10.16 ± 0.3
	5	0.669	0.668	0.675	0.670667	0.002186	87.55439513	12.44560487	
	2.5	0.729	0.752	0.755	0.745333	0.008212	97.30200174	2.697998259	
	1.25	0.777	0.748	0.756	0.760333	0.008647	99.26022628	0.739773716	
	0.625	0.761	0.774	0.762	0.765667	0.004177	99.9564839	0.043516101	
	0.312	0.77	0.759	0.762	0.763667	0.003283	99.69538729	0.304612707	

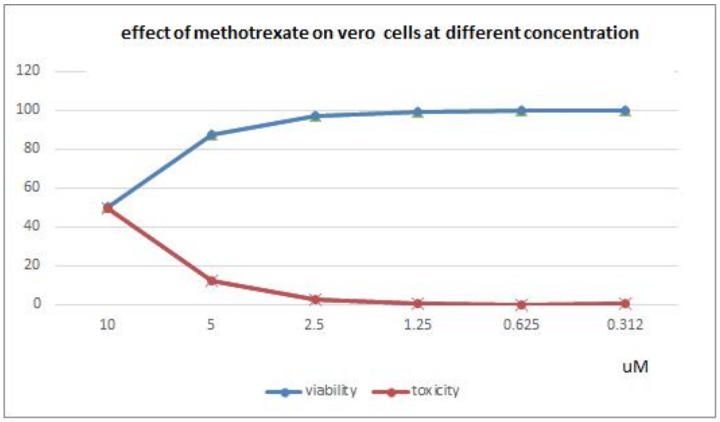

BRD4 Lab Report

BDR4

code	IC50	conc	log	%inh	T2	T1	ΔT	RFU2	RFU1	ΔRFU	slope	K.Activity
s3 (6b)		100	2	94	30	0	30	5.88	0	5.88	3.33333	7.056007
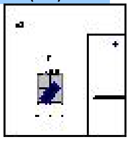												
		10	1	83	30	0	30	16.71	0	16.71	3.33333	20.05202
		1	0	67	30	0	30	33.02	0	33.02	3.33333	39.62404
		0.1	-1	46	30	0	30	54.16	0	54.16	3.33333	64.99206
		0.01	-2	30	30	0	30	69.88	0	69.88	3.33333	83.85608
EC				0	30	0	30	100	0	100	3.33333	120
		[redacted]		[redacted]	[redacted]		[redacted]		[redacted]		[redacted]	

code	IC50	conc	log	%inh	T2	T1	ΔT	RFU2	RFU1	ΔRFU	slope	K.Activity
s8 (7)		100	2	95	30	0	30	4.59	0	4.59	3.33333	5.508006
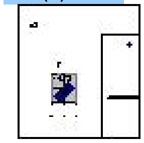												
		10	1	89	30	0	30	11.21	0	11.21	3.33333	13.45201
		1	0	74	30	0	30	26.42	0	26.42	3.33333	31.70403
		0.1	-1	56	30	0	30	44.13	0	44.13	3.33333	52.95605
		0.01	-2	38	30	0	30	62.03	0	62.03	3.33333	74.43607
EC				0	30	0	30	100	0	100	3.33333	120
		[redacted]		[redacted]	[redacted]		[redacted]		[redacted]		[redacted]	

code	IC50	conc	log	%inh	T2	T1	ΔT	RFU2	RFU1	ΔRFU	slope	K.Activity
s9 (4)		100	2	95	30	0	30	4.51	0	4.51	3.33333	5.412005
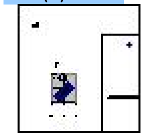												
		10	1	91	30	0	30	8.97	0	8.97	3.33333	10.76401
		1	0	75	30	0	30	24.57	0	24.57	3.33333	29.48403
		0.1	-1	58	30	0	30	42.46	0	42.46	3.33333	50.95205
		0.01	-2	41	30	0	30	59.48	0	59.48	3.33333	71.37607
EC				0	30	0	30	100	0	100	3.33333	120
		[redacted]		[redacted]	[redacted]		[redacted]		[redacted]		[redacted]	

code	IC50	conc	log	%inh	T2	T1	ΔT	RFU2	RFU1	ΔRFU	slope	K.Activity
s14 (6a)		100	2	95	30	0	30	5.42	0	5.42	3.33333	6.504007
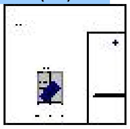												
		10	1	88	30	0	30	12.37	0	12.37	3.33333	14.84401
		1	0	73	30	0	30	26.61	0	26.61	3.33333	31.93203
		0.1	-1	49	30	0	30	51.46	0	51.46	3.33333	61.75206
		0.01	-2	34	30	0	30	65.58	0	65.58	3.33333	78.69608
EC				0	30	0	30	100	0	100	3.33333	120
		[redacted]		[redacted]	[redacted]		[redacted]		[redacted]		[redacted]	

code	IC50	conc	log	%inh	T2	T1	ΔT	RFU2	RFU1	ΔRFU	slope	K.Activity
Volasertib 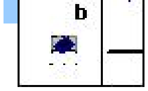		100	2	96	30	0	30	3.86	0	3.86	3.33333	4.632005
		10	1	91	30	0	30	8.76	0	8.76	3.33333	10.51201
		1	0	77	30	0	30	22.51	0	22.51	3.33333	27.01203
		0.1	-1	62	30	0	30	38.16	0	38.16	3.33333	45.79205
		0.01	-2	43	30	0	30	56.59	0	56.59	3.33333	67.90807
EC				0	30	0	30	100	0	100	3.33333	120
		[redacted]		[redacted]	[redacted]		[redacted]		[redacted]		[redacted]	

PLK1 Lab Report

PLK1

code	IC50	conc	log	%inh	T2	T1	ΔT	RFU2	RFU1	ΔRFU	slope	K.Activity
s3 (6b)		100	2	94	30	0	30	6.48	0	6.48	3.33333	7.776008
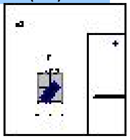		10	1	80	30	0	30	19.59	0	19.59	3.33333	23.50802
		1	0	65	30	0	30	34.66	0	34.66	3.33333	41.59204
		0.1	-1	40	30	0	30	59.82	0	59.82	3.33333	71.78407
		0.01	-2	28	30	0	30	72.43	0	72.43	3.33333	86.91609
EC				0	30	0	30	100	0	100	3.33333	120

code	IC50	conc	log	%inh	T2	T1	ΔT	RFU2	RFU1	ΔRFU	slope	K.Activity
s8 (7)		100	2	95	30	0	30	5.03	0	5.03	3.33333	6.036006
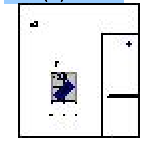		10	1	90	30	0	30	9.88	0	9.88	3.33333	11.85601
		1	0	76	30	0	30	23.56	0	23.56	3.33333	28.27203
		0.1	-1	60	30	0	30	39.89	0	39.89	3.33333	47.86805
		0.01	-2	43	30	0	30	57.46	0	57.46	3.33333	68.95207
EC				0	30	0	30	100	0	100	3.33333	120

code	IC50	conc	log	%inh	T2	T1	ΔT	RFU2	RFU1	ΔRFU	slope	K.Activity
s9 (4)		100	2	95	30	0	30	4.63	0	4.63	3.33333	5.556006
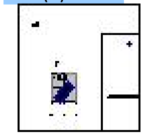		10	1	88	30	0	30	11.53	0	11.53	3.33333	13.83601
		1	0	70	30	0	30	29.72	0	29.72	3.33333	35.66404
		0.1	-1	46	30	0	30	53.61	0	53.61	3.33333	64.33206
		0.01	-2	34	30	0	30	65.88	0	65.88	3.33333	79.05608
EC				0	30	0	30	100	0	100	3.33333	120

code	IC50	conc	log	%inh	T2	T1	ΔT	RFU2	RFU1	ΔRFU	slope	K.Activity
s14 (6a)		100	2	94	30	0	30	5.71	0	5.71	3.33333	6.852007
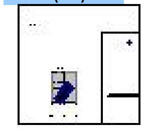		10	1	87	30	0	30	13.22	0	13.22	3.33333	15.86402
		1	0	65	30	0	30	35.42	0	35.42	3.33333	42.50404
		0.1	-1	42	30	0	30	58.11	0	58.11	3.33333	69.73207
		0.01	-2	25	30	0	30	74.54	0	74.54	3.33333	89.44809
EC				0	30	0	30	100	0	100	3.33333	120

code	IC50	conc	log	%inh	T2	T1	ΔT	RFU2	RFU1	ΔRFU	slope	K.Activity
Volasertib		100	2	96	30	0	30	4.33	0	4.33	3.33333	5.196005
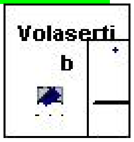		10	1	91	30	0	30	9.26	0	9.26	3.33333	11.11201
		1	0	76	30	0	30	23.53	0	23.53	3.33333	28.23603
		0.1	-1	58	30	0	30	42.08	0	42.08	3.33333	50.49605
		0.01	-2	42	30	0	30	58.17	0	58.17	3.33333	69.80407
EC				0	30	0	30	100	0	100	3.33333	120

Gene expression on MDA-MB-231 of 7

[redacted]

Sample			Bax							
			Control cells			Test cells				FLD
Ser	code	Conc	GAPDH	Bax	ΔCTC	GAPDH	Bax	ΔCTE	ΔΔ CT	2^ ΔΔCT
			HC	TC	TC-HC	HE	TE	TE-HE	ΔCTE-ΔCTC	E=1.842
1	s8 (7)		21.42	32.85	11.43	21.77	29.94	8.17	-3.26	7.3256
2	volasertib		21.42	32.85	11.43	21.59	30.28	8.69	-2.74	5.332
3	Control		21.42	32.85	11.43	21.42	32.85	11.43	0	1

Sample			Bcl2							
			Control cells			Test cells				FLD
Ser	code	Conc	GAPDH	Bcl2	ΔCTC	GAPDH	Bcl2	ΔCTE	ΔΔ CT	2^ ΔΔCT
			HC	TC	TC-HC	HE	TE	TE-HE	ΔCTE-ΔCTC	E=1.842
1	s8 (7)		21.42	27.29	5.87	21.77	29.61	7.84	1.97	0.3002
2	volasertib		21.42	27.29	5.87	21.59	29.17	7.58	1.71	0.3518
3	Control		21.42	27.29	5.87	21.42	27.29	5.87	0	1

Sample			Casp3							
			Control cells			Test cells				FLD
Ser	code	Conc	GAPDH	Casp3	ΔCTC	GAPDH	Casp3	ΔCTE	ΔΔ CT	2^ ΔΔCT
			HC	TC	TC-HC	HE	TE	TE-HE	ΔCTE-ΔCTC	E=1.842
1	s8 (7)		21.42	33.04	11.62	21.77	31.16	9.39	-2.23	3.9048
2	volasertib		21.42	33.04	11.62	21.59	30.41	8.82	-2.8	5.5311
3	Control		21.42	33.04	11.62	21.42	33.04	11.62	0	1
